# Sodium-based donor–acceptor assemblies featuring thermally activated delayed fluorescence enabled by highly efficient through-space charge transfer

**DOI:** 10.1039/d5sc08432f

**Published:** 2025-12-29

**Authors:** Ondřej Mrózek, Tabea Heil, Lukáš Hanzl, Andrey Belyaev, Indranil Sen, Patrick Pilch, Zhe Wang, Andreas Steffen

**Affiliations:** a Department of Chemistry and Chemical Biology, TU Dortmund University Otto-Hahn-Str. 6 44227 Dortmund Germany mrozek@iic.cas.cz andreas.steffen@tu-dortmund.de; b Department of General and Inorganic Chemistry, Faculty of Chemical Technology, University of Pardubice Studentská 573 532 10 Pardubice Czech Republic; c Department of Physics, TU Dortmund University Otto-Hahn-Str. 4a 44227 Dortmund Germany

## Abstract

Exploring more economical and innovative alternatives to precious 4d and 5d metal complexes used in photocatalysis, OLEDs, or energy conversion has primarily focused on 3d metals, leaving highly abundant alkali metals underexplored. We show that, under stoichiometry control of 1,10-phenanthroline-5-carbonitrile (^CN^phen) and sodium 2,6-bis(trimethylsilyl)benzenethiolate Na(^TMS^BT), sodium complexes can be obtained that either form the neutral linear assembly [Na(THF)(^CN^phen)(^TMS^BT)]_N_ (Na1D) or {[Na(^CN^phen)_4_](^TMS^BT)}_N_ (Na2D) as a 2D-polymeric arrangement with octahedrally coordinated Na, similar to 18 valence electron transition metal complexes, and the thiolate acting as a counter anion. Na1D and Na2D feature bright visible light absorption and thermally activated delayed fluorescence (TADF) from intra- and intermolecular through-space charge-transfer (^1/3^TSCT) excited states, respectively, with high radiative rates *k*_TADF_ up to 4.5 × 10^5^ s^−1^, unprecedented for alkali metal-based luminophores. Solution studies revealed extensive dynamic behavior, including reversible metal–ligand bond dissociation. However, the Na assemblies show ^1/3^TSCT emission (*λ*_em,max_ = 555 nm) in solution and for the first time we have successfully employed Na-based compounds as visible light photosensitizers in Dexter energy transfer catalysis. This study demonstrates the critical role of TSCT states in constructing photoactive coordination complexes and indicates underexplored, yet significant, potential of sodium-based luminophores as TADF emitters and earth-abundant photocatalysts.

## Abstract 2

Through-space charge-transfer in Na-based luminophores leads to efficient TADF, and the triplet states can be harvested for Dexter energy transfer.

## Graphical abstract

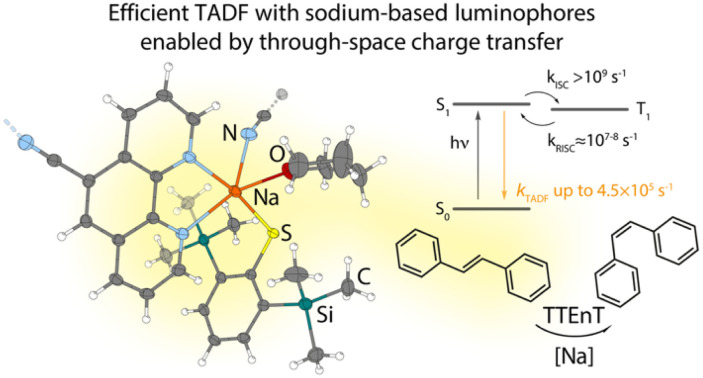

## Introduction

The photoluminescent (PL) phenomenon is essential in many application fields, including organic light-emitting diodes,^1,2^ photocatalysis,^3,4^ sensing,^5–7^ and molecular imaging.^8,9^ Particular emphasis of current research has been laid on developing new photocatalysts and emitters with high radiative rate constants *k*_r_ for electroluminescent devices, applications for which the efficient generation of electronically excited triplet states is highly beneficial. For example, while the formally spin-forbidden intersystem-crossing (ISC) S_*n*_ → T_1_ promotes photoreactivity due to an extended excited state lifetime in comparison to the fluorescent S_1_ state, triplet state emission allows for unitary exciton utilization in devices for maximum efficiency.^10^ An interesting emission mechanism for many of the abovementioned applications is thermally activated delayed fluorescence (TADF),^11^ which involves thermally-driven reverse ISC (rISC) T_1_ → S_1_, ultimately leading to fast, spin-allowed emission from the higher-lying singlet excited state. Thus, spin-forbidden phosphorescence is bypassed, but triplet excitons can still be harvested. TADF is only enabled for systems with a small singlet–triplet excited state energy gap (Δ*E*_ST_), typically below 200 meV.^12^ This can be achieved by constructing a donor–acceptor (D–A) motif that leads to charge transfer (CT) excited states with a spatially well-separated electron–hole pair, reducing their exchange-interaction integral that is proportional to Δ*E*_ST_.

Recent efforts to explore new PL complexes based on earth-abundant 3d metals led to the discovery of several groundbreaking examples that can compete with traditionally employed compounds based on precious heavy 4d and 5d transition metals, either due to their high luminescent performance or photo(redox)catalysis-relevant properties. Although complexes of 3d metals with d^1^–d^9^ electron configurations currently do not offer photophysical properties suitable for the application in electroluminescent devices due to the fast non-radiative decay *via* metal-centered (MC) states,^13^ a handful of examples based on, *e.g.*, Cr, Co or Fe metal cores have been engaged in relevant photochemistry, including photoredox catalysis and photo-induced energy transfer catalysis, displaying, in some instances, activity competitive with heavy metal-based photosensitizers.^14–16^ A d^10^ configuration, on the other hand, allows reaching highly emissive complexes with *k*_r_ that might overcome the second generation of phosphorescent OLED emitters. Cu^I^ compounds with archetypal carbene–copper–amide (CCA) structural motifs are well-explored in this field. A TADF mechanism was observed for CCA complexes with highly efficient emission featuring near-unity quantum yield.^17^ Notably, extensive tunability of photophysical properties is characteristic of this type of emitters. For instance, the emission wavelength can be manipulated by modulating the energy of the LUMO,^18,19^ comprising the π* orbital located on the carbene. Additionally, the radiative rate constant might be improved by reducing Δ*E*_ST_.^20,21^ Besides Cu^I^, carbene zinc(ii) thiolate (CZT) TADF emitters are currently on the rise.^22,23^ Very recently we demonstrated that structural control of the relative ligand orientation in trigonal planar CZT compounds enables TADF from ligand-to-ligand/ligand-to-metal ^1/3^(LL/LM)CT excited states with *k*_r_ in the range of 10^6^ s^−1^, faster than commercially used Ir-based emitters.^23^ Luminescent 3d metal complexes are experiencing a golden era; however, additional highly abundant metals should also be considered as potential substitutes or complements to expensive heavy transition metal luminophores.

In principle, the alkali metal sodium can be considered a very promising candidate for constructing new photoluminescent complexes due to its very high abundance of 2.36% in the Earth's crust. Sodium complexes feature a flexible coordination sphere and show great structural diversity, which are additional significant benefits for exploring novel photoluminescent motifs. This chemical behavior can be ascribed to the negligible covalent character of the Na^+^–L bonds, leaving coulombic interactions the dominant attractive force responsible for the less energetic and more flexible bonding between the metal ion and ligands. Unfortunately, such high molecular flexibility is often detrimental to photoluminescence due to pronounced vibrational relaxation to the ground state S_0_. Thus, sodium-based molecules have barely been considered as luminescent materials in the literature.

**Fig. 1:**
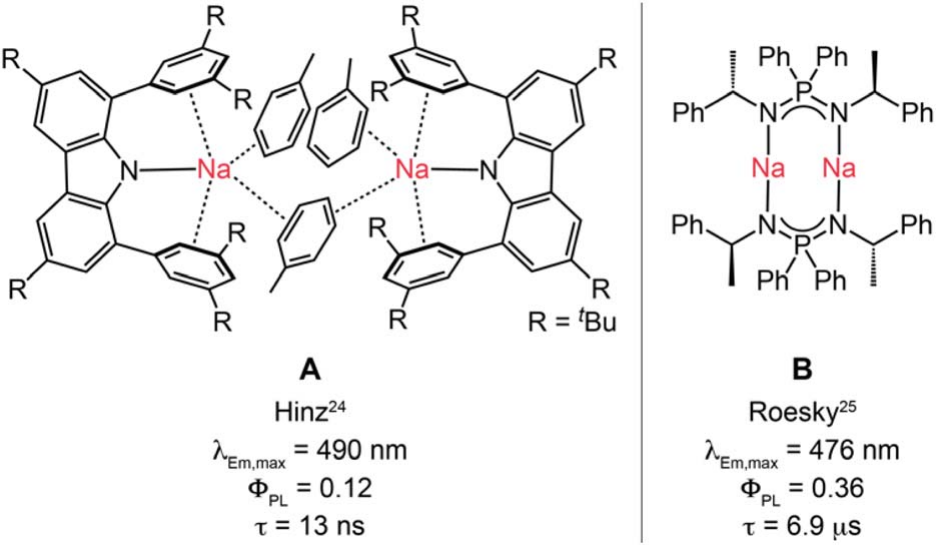
Literature-known Na^+^-based photoluminescence systems. Fluorescent (A) and TADF (B) complexes of sodium featuring intra-ligand CT and selected photophysical data in the solid state at room temperature.

One of the rare examples of a sodium-based luminescent compound was reported recently by Hinz and coworkers, who observed prompt singlet excited state emission from toluene-coordinated Na^+^ carbazolide complexes in the solid state with no evidence for ISC (Fig. 1A).^24^ The latter finding suggests that spin–orbit coupling (SOC) is insufficient in these light metal complexes to foster the formation of triplet states. Roesky and co-workers prepared a dimeric sodium complex of iminophosphonamides (Fig. 1B) featuring TADF, for which triplet formation was attributed to symmetry-breaking intra-ligand CT.^25^ Although this strategy can promote ISC for specific dimeric molecules,^26,27^ it comes at the price of reduced oscillator strength and a long S_1_ lifetime of *τ* = 230 ns (Fig. 1B), resulting in a relatively low *k*_TADF_ of 5 × 10^4^ s^−1^, as well as limited structural flexibility for photophysical modification.

Herein, we focused on a fundamentally different strategy to achieve ISC for triplet excited state formation and emission in sodium complexes, which is based on introducing spatially separated electronic donor and acceptor moieties in the first coordination sphere of the Na^+^ ion. The acceptor unit used in this work comprises 1,10-phenanthroline-5-carbonitrile (^CN^phen), whereas the donor fragment is based on 2,6-bis(trimethylsilyl)benzenethiolate (^TMS^BT). In the case of ^CN^phen, the nitrile function is expected to strengthen π-electrophilicity due to a strong electron-withdrawing effect. In addition, the steric demand of ^TMS^BT should lead to rigidification of the system, hindering detrimental vibrational relaxation. Moreover, TMS groups with high electron-donating properties make ^TMS^BT an exceptionally strong π-donor.

## Results and discussion

### Synthesis and molecular structures

**Fig. 2:**
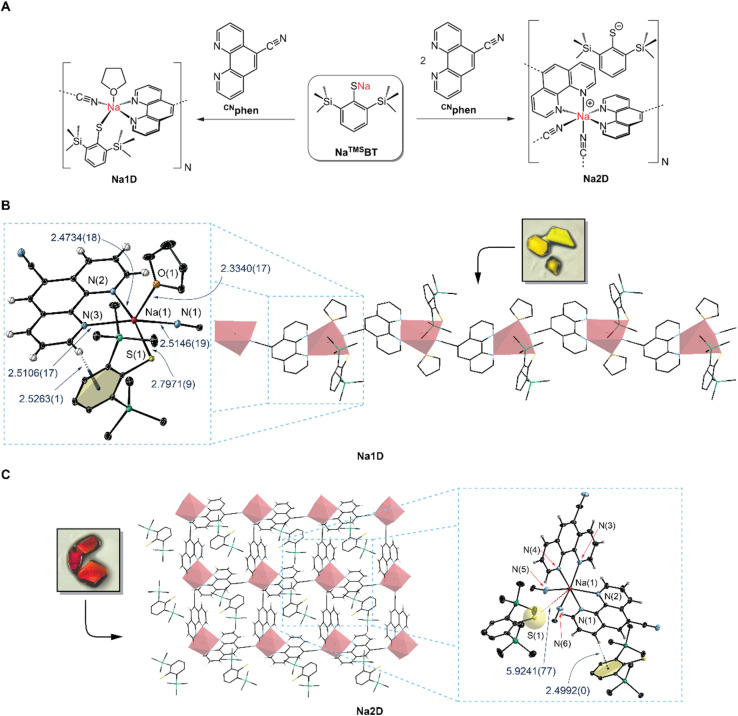
Synthesis, structures, and macromolecular architectures of Na1D and Na2D. (A) Synthesis of the sodium-based assemblies. (B and C) Molecular structures of monomeric units of Na1D and Na2D and their macromolecular architectures. Insets show photographs of single crystals of each assembly. Thermal ellipsoids are drawn at a 30% probability level. Crystal solvents (*n*-pentane for Na1D and THF for Na2D) are omitted for clarity. Selected interatomic distances are given in Å.

Our investigation of sodium-based donor–acceptor systems was commenced by mixing different ratios of ^CN^phen and Na^TMS^BT (Fig. 2A) in tetrahydrofuran (THF), which affords orange to red solutions from initially colorless reagents. Vapor diffusion of *n*-pentane into THF solution containing a 1 : 1 ratio of ^CN^phen : Na^TMS^BT yielded yellow single crystals, whereas, for a 2 : 1 ratio, the formation of a deep-red single-crystalline material was observed. Both types of crystals are sensitive to moisture as decolorization occurs under ambient conditions. X-ray diffraction analysis of the yellow sample revealed an unprecedented linear assembly (Na1D, Fig. 2B). In contrast, the red crystals consist of a two-dimensional coordination assembly denoted as Na2D (Fig. 2C).

For Na1D, each Na^+^ ion is coordinated by one THF molecule, ^TMS^BT, a chelating ^CN^phen ligand, and a bridging –CN group from the neighboring unit, leading to a distorted square-pyramidal geometry around the metal ion. Interatomic distances Na–N(^CN^phen) of 2.5106(17) and 2.4734(18) Å are in the range of values reported for phenanthroline complexes of sodium with various coordination geometries.^28–32^ Likewise, the Na–NC(^CN^phen) bond length of 2.515(2) Å shows minor distinctions compared to values reported for diverse sodium complexes bearing aliphatic^33,34^ or aromatic^35–37^ nitriles, typically falling in the 2.39–2.52 Å range. The Na–S bond length of 2.7971(9) Å is slightly elongated by *ca.* 0.05–0.1 Å in comparison to sodium complexes containing only solely coordinated thiolate ligands, *e.g.*, dimeric [Na^Ar^BT]_2_ (^Ar^BT = 2,6-bis(2,4,6-triisopropylphenyl)benzenethiolate or 2,6-bis(2,4,6-trimethylphenyl)benzenethiolate).^38,39^ In contrast, sodium benzenethiolates bearing additional σ-donors, such as multidentate aliphatic amines, exhibit Na–S distances typically in the range of 2.78–2.84 Å, which are slightly elongated compared to Na1D.^40^ The Na2D assembly is based on a monomeric unit comprising two ^CN^phen coordinated to Na^+^ in a typical chelating fashion, with two additional –CN bridging groups from neighboring units completing the pseudo-octahedral coordination environment. While the Na–N(^CN^phen) and Na–NC(^CN^phen) interatomic distances are comparable to the Na1D assembly, ^TMS^BT acts as an outer sphere counter anion in the voids of the two-dimensional network, as evidenced by the long S–Na interatomic distance of 5.9241(77) Å (Fig. 2B).

### Non-covalent interactions of Na1D and Na2D

We observed that the ^CN^phen and ^TMS^BT ligands in Na1D adopt a close-to-perpendicular orientation with a dihedral angle of 78.6°, presumably as a consequence of a weak non-covalent C–H⋯π interaction, as indicated by the short distance of 2.5263(1) Å between the hydrogen atom in the 2-position of the phenanthroline ligand and the benzene centroid of ^TMS^BT (Fig. 2B). Although the ^TMS^BT anions in the network voids of Na2D do not interact with the sodium ions, their orthogonal orientation relative to the ^CN^phen ligands allows for dense packing with T-shape geometries and a closest spatial separation of 2.4992(0) Å. Such a short distance also suggests significant C–H⋯π non-covalent intermolecular interaction. In addition, the ^CN^phen ligands of Na2D also interact non-covalently with neighboring ^CN^phen, as indicated by the tilted T-shape (TT) arrangements and C–H⋯π separation in the range of 2.95–3.45 Å (for details, see the SI). Within each monomeric unit of Na2D, this type of TT interaction is always maintained for the pair of ^CN^phen ligands: one bound in a bidentate fashion and the second *via* the –CN group to the same Na^+^ cation. We note that the N–Na–N angle defined by the nitrile and heterocyclic nitrogen in the *trans*-position of the nitrile is only 152° because the intermolecular C–H⋯π interactions distort the octahedral coordination geometry of the Na^+^ ion. The realization of ultra-short D–A distances below 2.8 Å for strong through-space electronic communication (TSEC) is challenging, especially for organic systems, where donor and acceptor fragments are linked *via* a rigid, covalent spacer. Compared with organic and organometallic TADF emitters for which non-covalent D–A coupling was rationally designed,^41–46^ the C–H⋯π distances between ^CN^phen and ^TMS^BT of *ca*. 2.5 Å in Na1D and Na2D are exceptionally short and close to the calculated separation for a benzene T-shaped dimer, providing substantial stabilization energy.^47^ The fact that bonding in sodium complexes mainly arises from coulombic interactions, which are generally weaker and more flexible than covalent bonds, results in a structure-determining effect of the ^TMS^BT⋯^CN^phen and ^CN^phen⋯^CN^phen interactions, allowing for the ultra-short distances between these D–A pairs. This aspect of Na-based complexes has game-changing potential for constructing molecular systems where enhanced non-covalent interactions are required, for instance, in materials science or drug design.^48,49^ TSEC between donor and acceptor ligands also bears great potential for the development of new emitter classes as it promotes the formation of through-space charge transfer (TSCT) excited states,^41,43,44,50,51^ which commonly enhances rISC by reducing Δ*E*_ST_ due to the absence of through-bond D–A conjugation. As a result, non-radiative deactivation can be suppressed in favor of improved PL performance.^42,44,52,53^ However, the decisive parameter affecting the efficiency of TSEC is the spatial D–A separation, since a distance beyond *ca.* 3.0 Å may lead to a substantial decrease in the CT rate.^50^

### Photophysical properties and emission mechanism in the solid

**Fig. 3:**
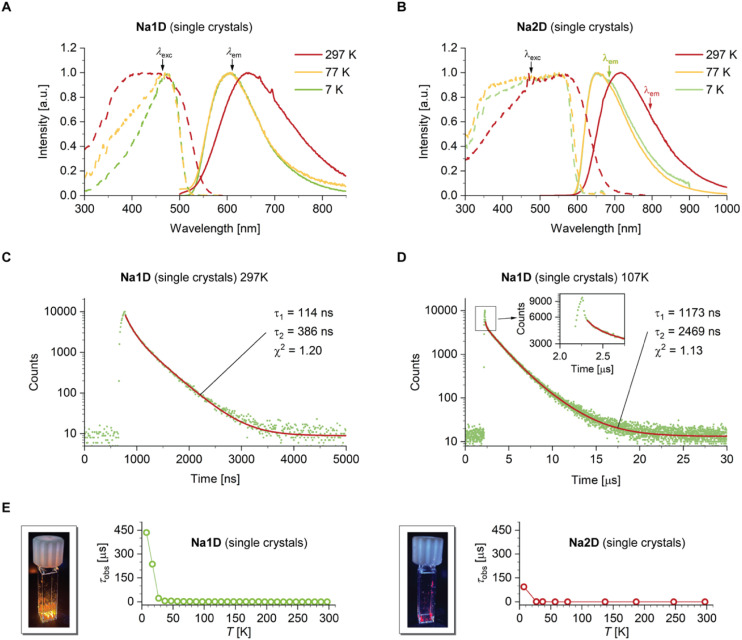
Photophysical characterization of Na1D and Na2D. Top: variable-temperature emission (solid lines) and excitation (dashed lines) spectra of single-crystalline samples of Na1D (A) and Na2D (B). Middle: time-resolved excited state decays of Na1D (*λ*_ex_ = 450 nm, *λ*_em_ = 600 nm) recorded at 297 (C) and 107 K (D). The inset shows a detail of the short-lived component observed at lower temperatures. Bottom: temperature-dependence of the observed lifetime recorded for single-crystalline samples of Na1D (E, left) and Na2D (E, right) and photographs of the luminescence of single crystals of Na1D and Na2D at room temperature upon 365 nm irradiation.

Surprisingly, upon 450 nm light irradiation, single crystals of Na1D and Na2D show bright orange to deep red luminescence at room temperature with very broad spectral appearance and *λ*_em,max_ of 645 and 715 nm, respectively, without tailing into the near-infrared region (NIR) (Table 1 and Fig. 3A, B and the inset of Fig. 3F). Such low emission energies are highly unusual not only for luminescent complexes of sodium but in general for alkali and earth-alkali metal-based compounds that typically feature blue to green fluorescence with *λ*_em,max_ below *ca*. 540 nm.^24,25,54,55^ According to our time-dependent density functional theory (TD-DFT) calculation (see below), this unique photophysical characteristic of Na1D and Na2D stems from a ^TMS^BT → ^CN^phen TSCT mechanism, which commonly red-shifts emission profiles due to weak electronic coupling and stabilization of the ground state *via* non-covalent interactions. Such modulation of emission energy is potentially of interest for future developments regarding applications where red-to-NIR emission is beneficial, such as bio-imaging^56^ or telecommunication.^57^ The relatively low photoluminescence quantum yields *Φ*_PL_ of 0.036 (Na1D) and 0.013 (Na2D) (Table 1) result from vibrational coupling between the ground and excited states according to the energy gap law,^58^ presumably enhanced by significant photo-initiated structural distortions of the assemblies as evidenced by the large apparent Stokes shifts and full widths at half maximum of 3956 (Na1D) and 3225 cm^−1^ (Na2D). Lifetime (LT) measurements revealed biexponential decays (Fig. 3C) with average values 〈*τ*〉 of 221 and 29 ns, affording estimated *k*_r_ of 1.6 (Na1D) and 4.5 × 10^5^ s^−1^ (Na2D), indicative of spin-forbidden triplet excited states being involved.

**Table 1:** Selected photophysical data for Na1D and Na2D (see also the SI)

	*T* [K]	*λ*_em_ [nm]	〈*τ*〉 [ns]^a^	*Φ*_PL_	*k*_r_ [s^−1^]^b^
Na1D solid^c^	297	645	221	0.036	1.6 × 10^5^
	77	607	2956	0.16	0.5 × 10^5^
	7	605	435 × 10^3^	—	—
Na2D solid^c^	297	715	29	0.013	4.5 × 10^5^
	77	656	251	0.053	2.1 × 10^5^
	7	656	125 × 10^3^	—	—
Na1D THF	297	555	—	—	—

a Amplitude average lifetimes.

b *k*_r_ = *Φ*_PL_/〈*τ*〉.

c Single-crystalline form.

Variable-temperature (VT) measurements for Na1D reveal an increase of 〈*τ*〉 and *Φ*_PL_ to 2956 ns and 0.16, respectively. The resulting slight decrease of *k*_r_ = 0.5 × 10^5^ s^−1^ in conjunction with the hypsochromic shift of the emission maximum from 645 to 607 nm (Δ*E* = 970 cm^−1^) at 77 K suggests temperature-dependent rigidification and changes in the Frank–Condon factors of the rather flexible emitter system. Upon further cooling, however, an enormous elongation of the excited state lifetime by more than three orders of magnitude compared to room temperature is observed to reach *ca.* 0.4 ms at 7 K (Fig. 3E left and Table 1). This cannot be explained by additional rigidification but rather by a change in the nature of the emitting excited state. Importantly, the emission spectrum at 7 K with *λ*_em,max_ = 605 nm shows only a minor additional hypsochromic shift by 55 cm^−1^ compared to the spectrum recorded at 77 K with no signs of vibrational progression, *i.e.*, the CT character of the emissive state is maintained even at very low temperature. Such a photophysical behavior is typical for a TADF mechanism with a very small energy gap Δ*E*_ST_ that allows the detection of emissive triplets only at very low temperatures, which we conclude to stem from the formation of interconverting ^1/3^TSCT states. A very similar behavior of the VT data was also found for Na2D (Fig. 3E right). It is important to note that for a wide temperature range, the experimentally obtained luminescence lifetime decays are bi-exponential, analogous to the 297 K data shown in Fig. 3C. However, we observed an additional short-lived component of *ca.* 0.9 to 1.0 ns (SI Fig. S47 and S48), whose contribution becomes larger with decreasing temperature in particular below *ca.* 170 K (see Fig. 3D and the inset). Since sulfur is the heaviest atom in the Na1D and Na2D systems, it is reasonable to expect the (r)ISC processes to be dominated by a spin-vibronic coupling mechanism. At low temperatures, where the vibrational motions are significantly suppressed, the rate/efficiency of spin-vibronic-driven ISC would be expected to be reduced. Thus, we attributed the short-lived components to residual prompt fluorescence from the ^1^TSCT state and excluded it from the 〈*τ*〉^TADF^ calculation, and we estimated the rate constant *k*_ISC_ at room temperature to be *ca.* 10^9^ s^−1^. Assuming that prompt fluorescence occurs with *Φ*_PL_ of 0.001–0.01, the resulting fluorescence rate *k*_F_ of 10^6^–10^7^ s^−1^ leaves us to conclude that reverse ISC occurs with *k*_rISC_ = 10^7^–10^8^ s^−1^ for Na1D and Na2D, which are compatible with established organic TADF emitters. The estimated values appear reasonable as the oscillator strength for prompt fluorescence from a ^1^TSCT state would be expected to be small, while a very small energy gap Δ*E*_ST_ would foster fast ISC and also rISC processes.

**Fig. 4:**
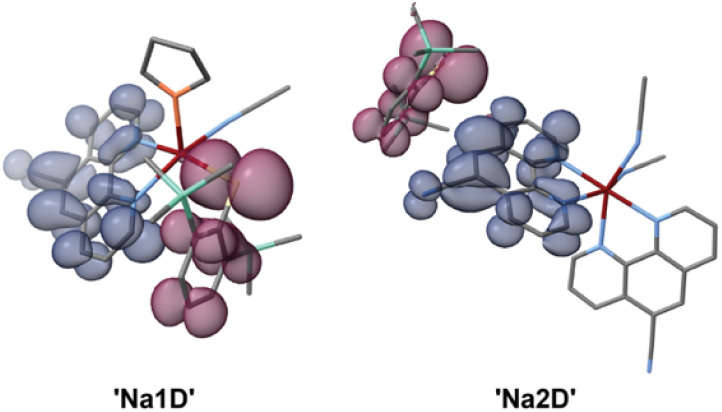
TD-DFT difference densities of the S_0_ → S_1_ excitations of monomeric units isolated from X-ray diffraction data of Na1D and Na2D. Areas losing electron density are depicted in purple, and areas gaining electron density in blue.

To gain deeper insight into the photophysical properties of Na1D and Na2D, we performed TD-DFT calculations on the monomeric units extracted from X-ray diffraction data, with the CN-coordinating phen ligand being substituted by acetonitrile as simplified models of the polymeric structures (for full details, see the SI), assuming localization of the excitons (see below). Under these approximations, the TD-DFT calculations provided energetically nearly identical S_1_ and T_1_ states (Fig. 4 and SI S49) with Δ*E*_ST_ values of only 2 (Na1D) and 31 meV (Na2D), which coincide with the proposed close-to-isoenergetic ^1/3^TSCT excited states, allowing for efficient (r)ISC processes in a TSCT–TADF mechanism (see above). Considering the polymeric nature of Na1D and Na2D, we further investigated whether the transport of optically excited charges in the form of exciton hopping can occur. For both compounds we obtained large single crystals suitable for time-resolved 400 nm pump terahertz (THz) transmission probe spectroscopic experiments with femtosecond time resolution.

**Fig. 5:**
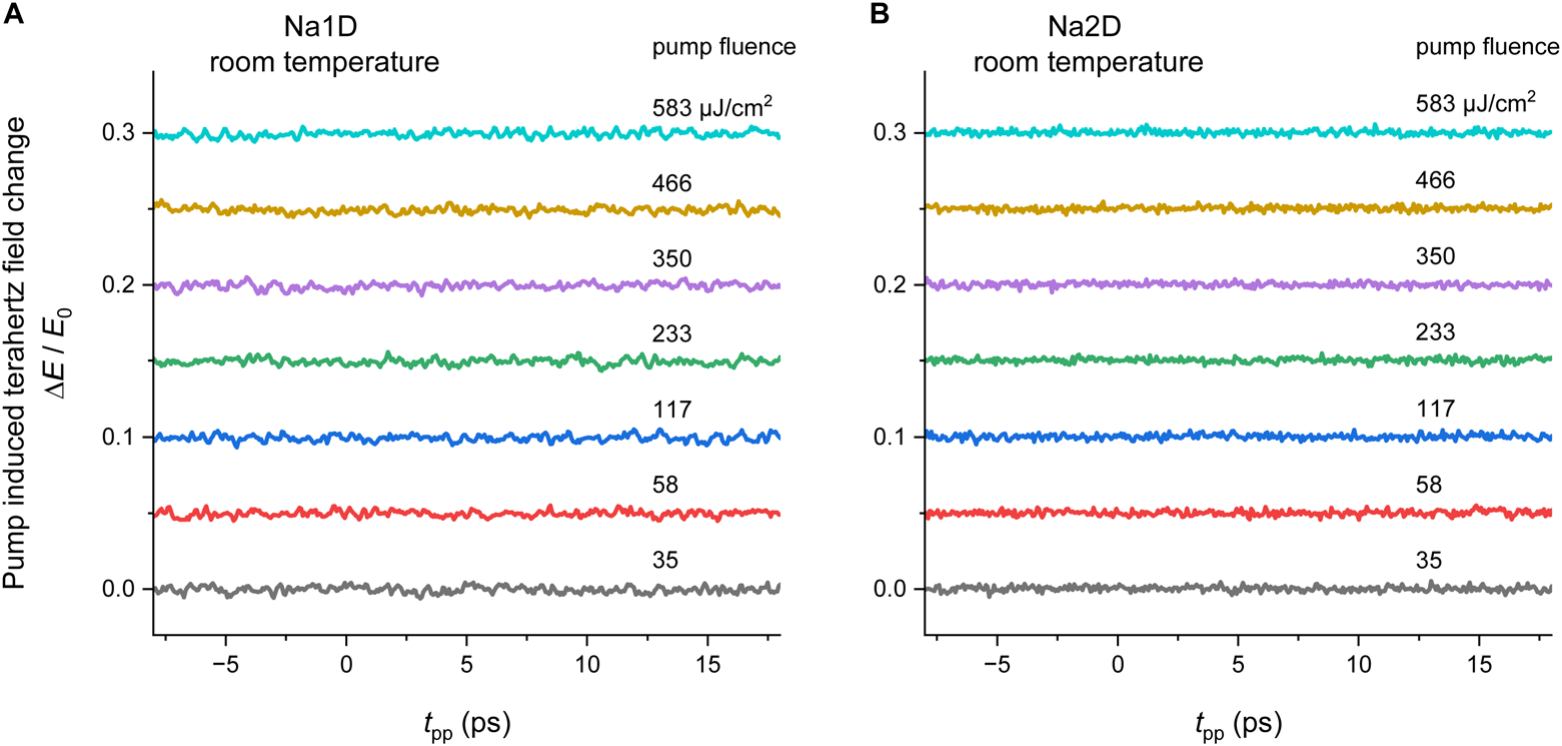
400 nm optical pump terahertz transmission probe spectroscopy of Na1D (A) and Na2D (B) at 297 K. The pump-induced change of the transmitted terahertz electric field Δ*E*/*E*_0_ is measured as a function of pump-probe time delay *t*_pp_ for various fluences of the 400 nm pump pulses, where *E*_0_ denotes the peak electric field of the terahertz probe pulse without the optical pump.

The time-resolved optical pump THz probe spectroscopy is a well-established approach to investigate charge carrier dynamics in nonequilibrium states.^59,60^ As presented in Fig. 5, the change of the transmitted THz electric field Δ*E* is recorded as a function of the time delay between the 400 nm excitation laser pulse and the peak electric field of the THz probe pulse. For a variety of pump fluences from 35 to 583 µJ cm^−2^, which is well above the threshold for the observation of photoluminescence, we cannot resolve any change in the THz probe pulse. This is in clear contrast to the situation of, *e.g.*, a semiconductor, where an optical excitation creates additional charge carriers (*i.e.*, electrons and holes), whose transport leads to enhanced absorption of the THz field.^60^ The observed absence of THz absorption indicates that the optically excited charges in Na1D and Na2D are mostly localized. Otherwise, the transport of these charges will not only result in absorption of the THz field but also in enhanced absorption at higher pump fluences.

Intramolecular TSCT-derived emission related to metal complexes is very rare and underexplored. Usually, the high degree of metal–ligand bond covalency restricts the molecular geometry and prevents its distortion from optimizing the D–A spatial separation and non-covalent interactions, limiting TS electronic coupling between donor and acceptor moieties. Consequently, TSCT excited states with transition metal complexes are restricted to very few specific molecular designs with limited chemical tunability. For instance, a rapid TSCT–TADF process due to an extremely low Δ*E*_ST_ of *ca*. 7 meV and a rISC rate constant up to 10^7^ s^−1^ was recently reported for a new type of CCA complex, consisting of a carbazolide donor and functionalized N-heterocyclic carbene (NHC).^42^ In this case, however, TSCT is only enabled for NHCs decorated with phenylsulfonyl flanking units that act as acceptors due to the strongly electron-withdrawing sulfonyl function,^42^ while the herein-reported sodium-based assemblies provide ample possibilities for further excited state modification.

### Coordination behavior in solution

An additional important photophysical feature of our TSCT assemblies Na1D and Na2D presents visible light absorption (excitation spectra in Fig. 3A and B), critical for many applications, such as photocatalysis. The direct comparison with the previously reported Na-based TADF emitter (Fig. 1, B), which can only be excited by UV light,^25^ clearly demonstrates the potential of TSCT donor–acceptor motifs to access luminescent alkaline metal complexes with visible-light absorption. Thus, we were further interested in whether a molecular species based on these assemblies, featuring TS electronic donor–acceptor communication, might persist in a solution for potential photocatalysis.

Despite the diverse solid-state structures of Na1D and Na2D, ^1^H and ^13^C{^1^H} NMR spectra in THF-d_8_ have an analogous character with only minor variations in chemical shifts (SI Fig. S25). In the ^1^H NMR spectra, the only significant spectral differences were found in the integral intensities of the observed resonances with the expected ratios for the ^TMS^BT and ^CN^phen ligands of 1 : 1 and 1 : 2 for Na1D and Na2D, respectively. Interestingly, the spectra show signals of one molecule THF (3.62 and 1.78 ppm) originating from a non-coordinated solvent.^61^ For Na2D, this is expected as single crystals used for NMR measurements contain one molecule of co-crystallized THF in the unit cell, while for Na1D, this implies either an exchange of coordinated THF for THF-d_8_ or a more complicated solution behavior of both assemblies, which appears to be independent of the ^TMS^BT : ^CN^phen ratio. ^1^H diffusion-ordered spectroscopy (DOSY) of Na1Ddissolved in THF-d_8_ revealed extensive ligand dissociation and the formation of free ^CN^phen, ^TMS^BT, and THF (for detailed ^1^H DOSY NMR analysis, see SI Fig. S28). Notably, ^1^H–^1^H nuclear Overhauser effect spectroscopy (NOESY) revealed NOE between the protons of the TMS group and those of ^CN^phen in position 9 or 2.

This observation suggests a close proximity of the respective protons, similar to the ligand arrangement found in the solid state, with a distance of only *ca.* 3.2 Å, and thus reversible dissociation from/association to sodium in a solution of ^CN^phen and ^TMS^BT. The exact Na^+^ complex solution structure is unclear and present only in very low concentrations, as this equilibrium is shifted towards mostly dissociated ligands, preventing identification by 1D NMR techniques or ^1^H DOSY NMR spectroscopy at room temperature. However, at −100 °C, the original ^1^H NMR signal of the protons in positions 2/9 on ^CN^phen (9.12 ppm at 297 K) splits into two doublets with chemical shifts of 9.32 and 9.28 ppm (SI Fig. S27), indicating a temperature-dependent reversible complexation–dissociation equilibrium that can be shifted towards the sodium complex under kinetic control. The ^15^N NMR resonance of heterocyclic nitrogen atoms is shifted upfield by 9.3 ppm at −100 °C to a value of −88.4 ppm, which is also expected upon the coordination of ^CN^phen with metal ions.^62^

### Absorption, emission, and photocatalytic triplet energy transfer properties in solution

**Fig. 6:**
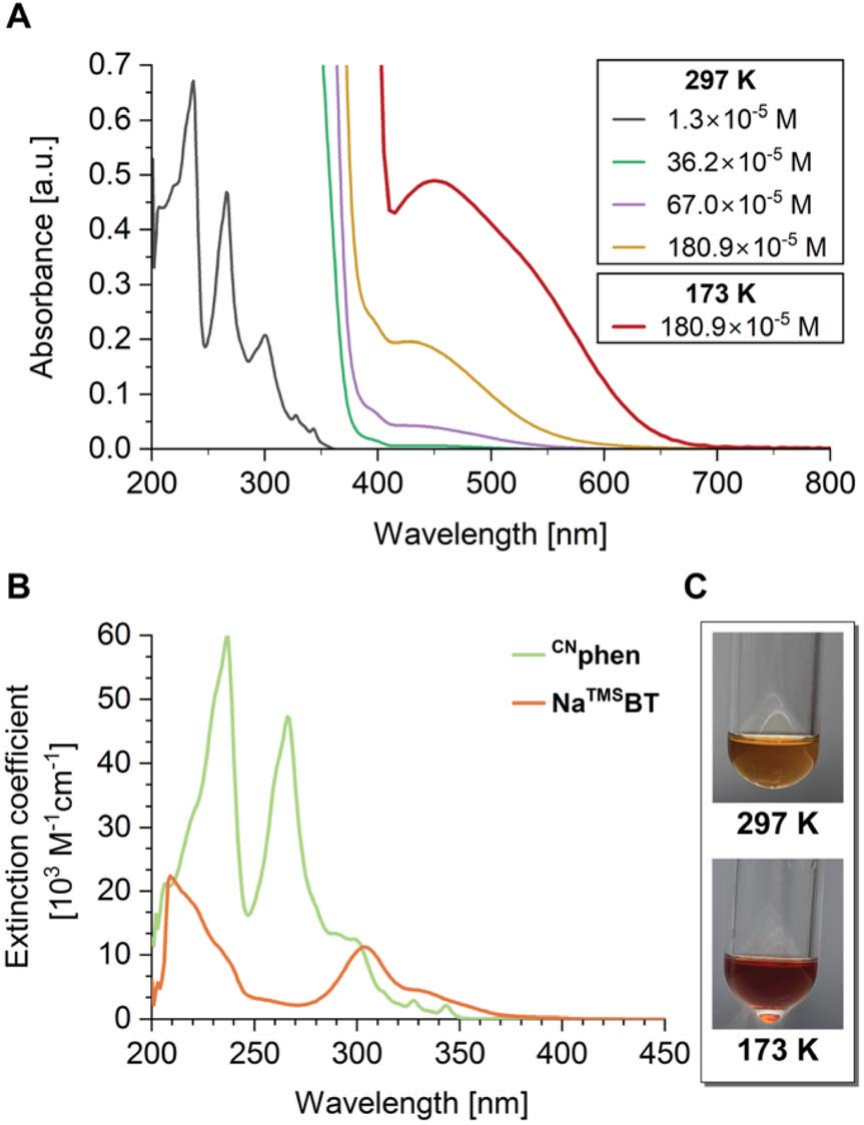
(A) Concentration-dependent absorption spectra recorded upon dissolving Na1D in THF. (B) Absorption spectra of ^CN^phen (green) and Na^TMS^BT (orange) in THF solution. (C) Photographs of solutions of Na1D (∼180 × 10^−5^ M) at 297 K (top) and 173 K (bottom).

At concentrations below *ca*. 10^−4^ M, the steady-state UV/Vis spectra of Na1D dissolved in THF show solely high-energy excitations below *ca.* 370 nm (Fig. 6A), which we attribute, by comparison, to π → π* and n(E) → π*(phen) (E = N, S) transitions of free colorless ^CN^phen and ^TMS^BT, respectively (Fig. 6B). However, at concentrations >5 × 10^−4^ M, an additional broad band in the visible region between 410 and 650 nm with *λ*_abs,max_ = 428 nm is observed that we assign to CT excitation between ^CN^phen and ^TMS^BT due to a concentration-dependent shift of the equilibria investigated by NMR spectroscopy (*vide supra*). Interestingly, previously reported coordination complexes bearing phen and benzenethiolate ligands, for example, [Zn(BT)_2_(phen)],^63,64^ are typically colorless with only near-UV LLCT excitations. Considering the pronounced tendency to form C–H⋯π non-covalent interactions in the solid state and the observed NOE between ^CN^phen and ^TMS^BT, proving the proximity of these two ligands in solutions even at room temperature, the occurrence of electrostatic-controlled low energy ^TMS^BT → ^CN^phen TSCT facilitated by the sodium ion would explain the shift into the visible region observed for dissolved Na1D. It is important to note that such intense low-energy absorption bands were not observed for pure solutions of ^CN^phen or Na^TMS^BT, even at higher concentrations (SI Fig. S40A and B), excluding the formation of ^CN^phen- or Na^TMS^BT-based solvent exciplexes. In line with the VT-NMR data discussed above, cooling THF solutions of Na1D also affects the color of the solution from light orange to red due to the equilibrium shift towards the sodium coordination complex (Fig. 6C). As shown in Fig. 6A, at 173 K (red traces), the low energy band shows a significant bathochromic shift to 450 nm with the apparent shoulder at *ca*. 550 nm and considerable broadening, leading to offset of the band up to 670 nm. Notably, the absorption intensity increases significantly by factors of *ca*. 2.5 and 10 at 450 and 550 nm, respectively. These findings further prove preferable complexation at low temperatures, for which more pronounced ^TMS^BT → ^CN^phen TSCT visible-light excitations are expected.

**Fig. 7:**
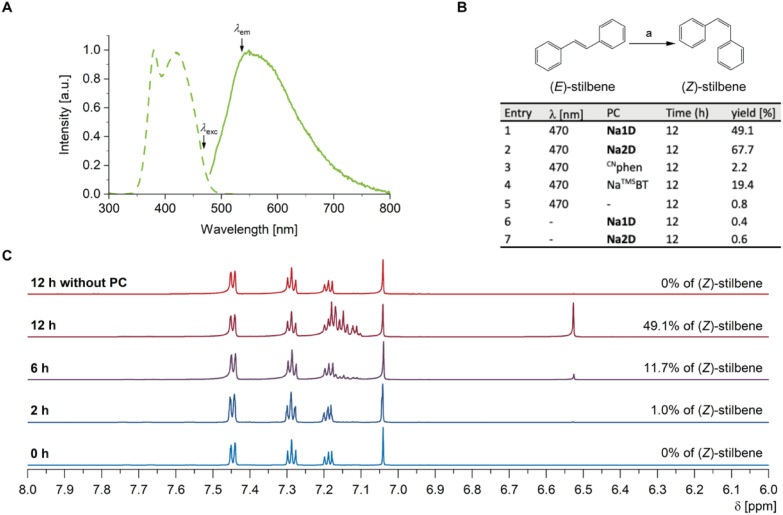
Solution luminescence and energy transfer. (A) Luminescence (solid) and excitation (dashed) spectra of Na1D in THF (*λ*_ex_ = 470 nm, *λ*_em_ = 525 nm). (B) Photoisomerization of (*E*)-stilbene, a = photocatalytic conditions are summarized in the table. (C) Example of the low-field part of ^1^H NMR spectra showing photocatalytic conversion of (*E*)-stilbene to (*Z*)-stilbene using Na1D solution and 470 nm irradiation.

Excitation into the visible absorption band enabled by TSCT transitions in the range of 470–500 nm resulted in very weak broad luminescence with *λ*_em,max_ = 555 nm (Table 1 and Fig. 7A), similar to the properties of Na1D in the solid state. Although we could not deduce the emission lifetime or quantum yield, the existence of triplet excited states in single crystals of Na1D under visible light excitation led us to assume that the luminescence of the assembly in solution is also TADF in nature. This would imply that dissolved Na1D may be suitable for homogeneous photocatalysis *via* Dexter energy transfer.

Photocatalytic (*E*)-to-(*Z*) isomerization *via* triplet energy transfer (EnT) is an important strategy for accessing stereodefined (*Z*)-alkenes as relevant building blocks in synthetic chemistry, which are typically thermodynamically less stable compared to their more common (*E*)-counterparts.^65,66^ The key concept behind the isomerization is the promotion of electron density from π to π* orbitals accompanied by lowering of the C=C bond order, allowing rotation and subsequent relaxation to the (*Z*)-alkene configuration. However, since the lowest π → π* singlet excitations typically require high-energy UV light, transition metal complexes that can be excited with visible light are typically used for sensitization of the alkene ^3^ππ* states.^67,68^ Bearing in mind the assumed triplet excited state energy of Na1D in solution of ∼2.23 eV (*λ*_em,max_ ∼ 555 nm), we employed our novel sodium-based systems for photo-driven isomerization of (*E*)-stilbene exhibiting a triplet energy of 2.13 eV as a proof of concept test reaction. Irradiation of degassed THF solutions containing (*E*)-stilbene and 5 mol% of dissolved Na1D or Na2D with 470 nm blue light promoted (*E*) → (*Z*) conversion within 12 h to give the desired product in 50% and 68% yield, respectively (Fig. 7B and C). Importantly, control experiments without light or using only ^CN^phen as a catalyst gave only negligible formation of (*E*)-stilbene under otherwise identical reaction conditions. Interestingly, we observed up to 19% conversion with Na^TMS^BT, presumably due to very weak direct S_0_ → T_1_ absorption of ^TMS^BT enabled by SOC from sulfur and subsequent EnT.

Recently, efficient energy and electron transfer photocatalysis using a coulombic dyad composed of tris(1,10-phenanthroline)ruthenium(ii) bound non-covalently to pyrene-1,3,6,8-tetrasulfonate was reported by Kerzig and coworkers*.*^69^ Our findings demonstrate for the first time that a similar approach is applicable also for simple organic alkaline metal salts, such as Na^TMS^BT, which might act as photoactive species in triplet EnT processes. Additional topical studies have shown that organic and transition metal photocatalysts can decompose within the first short period of the overall reaction time, although the conversion continues.^70^ Bearing in mind the coexistence of various salts in the reaction mixtures of typical photocatalytic applications, our study indicates that they may be involved in the decisive photoinitiation steps. In addition, the activity and required excitation wavelength can be efficiently modulated by employing or forming donor–acceptor motifs, which enhances visible-light absorption and likely promotes ISC due to lowering Δ*E*_ST_, as demonstrated by the application of Na1D and Na2D.

## Conclusion

In contrast to previous studies focusing on prompt fluorescence or TADF originating from intra-ligand CT states in sodium compounds, we have shown a new approach to foster emission involving triplet excited states in abundant alkaline metal complexes by generating through-space CT. The critical design motif we deduce from our case study is a D/A orientation around the Na ion that allows for non-covalent interactions. Generally, sodium complexes are dominantly stabilized by the coulombic character of the Na–ligand bonds, which are geometrically more flexible than those typically observed in transition metal complexes bearing a much higher degree of covalency. Consequently, the coordination geometry of the sodium ion in our compounds is distorted to accommodate the interligand interactions.

As a far-reaching consequence, strong D–A through-space coupling is observed for single-crystalline one- (Na1D) or two-dimensional (Na2D) coordination entities composed of Na^TMS^BT and ^CN^phen, leading to efficient ISC and rISC between the ^1/3^TSCT states due to their very low experimentally determined Δ*E*_ST_ of only *ca.* 55 cm^−1^ (7 meV), as found for Na1D. The enabled TADF process leads to deep red to NIR luminescence, which on its own is highly unusual for alkali- or earth-alkaline metal compounds,^24,25,54,55^ and high radiative rate constants of up to 4.5 × 10^5^ s^−1^ that are unprecedented for sodium-based emitters involving triplet excited states. Despite the rather complicated solution behavior, TSCT was also confirmed for the system obtained by dissolving Na1D in THF, giving emission at *λ*_max_ = 555 nm, leading for the first time to the successful application of a sodium complex as a photosensitizer for visible-light-driven triplet EnT, as evidenced by the photoisomerization of (*E*)-stilbene. Despite their insignificant SOC, our case study indicates great potential for highly abundant alkaline and earth-alkaline complexes in future photonic applications involving TADF or red-to-NIR luminescence, such as electroluminescent devices, bio-imaging, telecommunication, and sensitizer compounds for photocatalytic conversions.

## Author contributions

O. M. designed the research, wrote the paper, and performed experimental work, including syntheses, photophysical measurements, and data analysis; T. H. performed experimental work, including syntheses, photophysical measurements, and data analysis; A. B. carried out X-ray diffraction analysis; L. H. and I. S. contributed to synthetic work and photophysical measurements; P. P. and Z. W. performed THz measurements and wrote the respective section of the manuscript; A. S. designed the research, analyzed the data, supervised the project and wrote the paper. All authors discussed the results and commented on the manuscript.

## Conflicts of interest

There are no conflicts to declare.

## Supplementary Material

SC-OLF-D5SC08432F-s001

SC-OLF-D5SC08432F-s002

## Data Availability

CCDC 2288161 and 2288162 contain the supplementary crystallographic data for this paper.^71*a*,*b*^ Supplementary information (SI): synthesis details, characterisation data for compounds including NMR spectroscopy, mass spectrometry, single crystal X-ray diffraction data, theoretical studies, and photophysical data. See DOI: https://doi.org/10.1039/d5sc08432f.
