# Supplementary material for: Sodium-based donor–acceptor assemblies featuring thermally activated delayed fluorescence enabled by highly efficient through-space charge transfer
Source: Chem Sci. 2025 Dec 29;17(9):4803–13. doi: 10.1039/d5sc08432f (PMC12809462; doi:10.1039/d5sc08432f)
Supplement: SC-017-D5SC08432F-s001 [file SC-017-D5SC08432F-s001.pdf]

Supplementary Information (SI) for Chemical Science.
This journal is © The Royal Society of Chemistry 2025

Sodium-based Donor-Acceptor Assemblies Featuring Thermally Activated Delayed Fluorescence Enabled by Highly Efficient Through-Space Charge Transfer

Ondřej Mrózek[1]*, Tabea Heil[1], Lukáš Hanzl[1,2], Andrey Belyaev,[1] Indranil Sen,[1] Patrick Pilch[3], Zhe Wang[3] and Andreas Steffen[1]*

[1] Department of Chemistry and Chemical Biology, TU Dortmund University, Otto-Hahn-Str. 6, 44227 Dortmund (Germany)

[2] Department of General and Inorganic Chemistry, Faculty of Chemical Technology, University of Pardubice, Studentská 573, 532 10 Pardubice (Czech Republic)

[3] Department of Physics, TU Dortmund University, Otto-Hahn-Str. 4a, 44227 Dortmund (Germany)

E-mail: ondrej.mrozek@gmail.com, andreas.steffen@tu-dortmund.de

Contents

Methods and Materials 3
Synthesis 6
Sodium 2,6-bis(trimethylsilyl)benzenethiolate (Na^{TMS}BT) 6
1,10-phenanthroline-5-carbonitrile (^{CN}phen) 9
Na1D 11
Na2D 12
NMR Spectra 13
DOSY NMR analysis 39
X-ray diffraction data 48
Photophysical data 58
Photoisomerization of (*E*)-stilbene 65
DFT calculations 66
Difference densities 66
TD-DFT excitation data 67
XYZ coordinates 71
References 79

Methods and Materials

All operations were carried out under an argon atmosphere using standard Schlenk or glovebox techniques. The solvents were dried using the Technology Inc. Pure-Solv system or standard methods,[1] degassed, and stored under activated 4Å molecular sieves. Unless stated otherwise, all chemicals were obtained from commercial suppliers and used without purification. The target compounds **Na1D** and **Na2D** are moisture-sensitive, and exposure to air causes quick discoloration of samples in both solid and solution forms, likely due to water-induced protonation of the thiolate group.

NMR spectra were recorded on a Bruker AVANCE III HD NanoBay 400, Bruker AVANCE Neo 500, or Bruker AVANCE III HD 700. All chemical shifts δ are given in *parts per million* (ppm) relative to the residual signal of deuterated solvent (chloroform-d$_1$ ($CDCl_3$): δ_H = 7.26 ppm, δ_C = 77.16; benzene-d$_6$ (C_6D_6): δ_H = 7.16 ppm, δ_C = 128.06 ppm; tetrahydrofuran-d$_8$ (C_4D_8O): δ_H = 3.58, 1.72 ppm, δ_C = 67.21, 25.31 ppm).[2] For the ^{29}Si NMR spectra tetramethyl, silane was used as an external reference. The chemical shift values for the ^{15}N NMR spectra are given in the nitromethane scale.

The crystals of **Na1D** and **Na2D** were immersed in perfluoropolyether oil, mounted on a polyimide microloop (MiTeGen), transferred to a stream of cold nitrogen, and measured at a temperature of 100 K. The X-ray diffraction data were collected on a Bruker D8 diffractometer with a CMOS Photon 100 and multilayer optics monochromated Mo K-α (0.71073 Å) or Cu K-α (1.5406 Å) radiation (INCOATEC microfocus sealed tube). The frames were integrated with the Bruker SAINT software package using a narrow-frame algorithm. The APEX4 program package was used for cell refinements and data reductions. The structure was solved using the intrinsic phasing method,[3,4] refined and visualized with the OLEX2-1.5 program.[5] A semiempirical absorption correction (SADABS) was applied to all data. All non-hydrogen atoms were refined anisotropically. Hydrogen atoms were included using structure factors calculations. All Hydrogen atoms were assigned to idealized geometric positions. The crystallographic details are summarized in Supporting Table S3. The unit cells of the **Na2D** contain disordered THF solvent molecules which have been treated as a diffuse contribution to the overall scattering without specific atom positions by SQUEEZE/PLATON.[6] The crystallographic details are summarized in Table 1. CCDC **2288161** and **2288162** contain supplementary crystallographic data for this paper.

Attenuated total reflection infrared (IR-ATR) measurements were measured with a diamond ATR holder in reflection mode on a Bruker Alpha II inside a glovebox. The samples were measured in crystalline form, but the instrument’s stamp ground the samples slightly.

Thermogravimetric analysis was performed with an SDT 650 Simultaneous Thermal Analyzer by TA Instruments under nitrogen in an open 90 mL alumina crucibles, with 8.311023

mg of **Na1D** and 6.988114 mg **Na2D**, respectively, and a temperature range of 40 - 800 ºC. A 10 K/min heating rate, isothermal conditions for 1.0 min at 40 ºC, and a zinc reference were applied.

The masses of the species of **Na1D** and **Na2D** in solution were analyzed via *high-performance liquid chromatography time-of-flight mass spectrometry* (HPLC-TOF-MS) using an Agilent 1260 Infinity II system by Agilent Technologies consisting of a G7129A autosampler, a G7116A column oven, a G7117C photodiode array detector and a G7111B quaternary pump system. The measurement was performed on a compact QTOF by Bruker Daltonics GmBH & Co. KG.

Due to the polymeric character and high flexibility of **Na1D** and **Na2D**, caused by predominant electrostatic interaction, the (TD)-DFT calculations were performed on monomeric units isolated from X-ray diffraction data, and only hydrogens atoms were optimized. Geometry optimizations were carried out using ORCA 5.0.3 software package[7] by employing B3LYP functional[8-11] together with def2-svp basis set[12] and Grimme-D3 empirical dispersion correction.[13,14] Solvents effects (THF, ε = 7.25) were accounted for by implicit solvent model CPCM.[15] Difference densities at isovalue of 0.001 were prepared by using orca_plot module as implemented in ORCA 5.0.3. software package and visualized using ChimeraX graphical software.[16]

All photophysical measurements were performed under rigorous exclusion of air and moisture. Tetrahydrofuran for steady-state absorption and emission spectroscopy was dried and degassed before the measurement. The solid-state measurements were performed in single-crystalline form using a thin quartz EPR tube or 4 K cryostat sample holders, which allows sample preparation without applying mechanical stress to the crystals. UV-visible absorption spectroscopy was performed on an Agilent Cary 5000 spectrophotometer using standard 1 cm path-length quartz cells. Excitation and emission spectra were recorded on an Edinburgh Instrument FLS1000 spectrometer, equipped with a 450 W Xenon arc lamp, double monochromators for the excitation and emission pathways, and a red-sensitive photomultiplier (PMT-980) as a detector. The excitation and emission spectra were corrected using the standard corrections supplied by the manufacturer for the excitation source's spectral power and the detector's sensitivity. Quantum yields of solid samples and polymeric matrices were measured using an integrating sphere (N-M01) from Oxford Instruments. The luminescence lifetimes were measured using a μF2 pulsed 60 W Xenon microsecond flashlamp, with a repetition rate of 10-100 Hz and a multichannel scaling (MCS) module or with VPLED (449.6 nm with 37 mW), with 100 ns pulse width and an MCS module, depending on the time range. The emission was collected at a right angle to the excitation source. The Prompt fluorescence lifetimes were measured with EPLED – 365 (363.2nm, 927.3 ps pulse width, 40 μW) as an

excitation source, and data were collected using time-correlated single photon counting (TCSPC). The low-temperature experiments used a 4 K cryostat (CS204SI-FMX-1SS) from Advanced Research System equipped with a closed cycle water-cooled helium compressor. Photoisomerization experiments were performed using 470 nm 18W EvoluChem™ LED irradiation sources. For each photocatalytic reaction, a solution containing 1.4 mM of **Na1D**/**Na2D** and 28 mM of (*E*)-stilbene in degassed THF. The isomerization was monitored by ^{1}H NMR spectroscopy, and conversion was determined based on the integration of proton resonances (for summary, see Supporting Tables 6 and 7).

Time-resolved 400 nm pump terahertz probe spectroscopy was set up based on an femtosecond amplifier laser system (800 nm, 100 fs, 7 mJ). The 400 nm pump pulses were prepared by using second-harmonic generation at a β-BaB_2O_4 crystal. The terahertz radiation was generated by using optical rectification at a $LiNbO_3$ crystal. The transmitted terahertz field through the sample was detected by electro-optic sampling at a ZnTe crystal. The sample was mounted in an optical cryostat and under vacuum to avoid degradation. The time-resolved pump-probe spectroscopy measurements were performed at room temperature.

Synthesis

Sodium 2,6-bis(trimethylsilyl)benzenethiolate (Na^{TMS}BT)

Na^{TMS}BT was synthesized using commercial 2,6-dibromoaniline following the multistep procedure (Supplementary Fig. 1), previously employed for the preparation of 2,6-bis[bis(2-diisopropylphenylphosphino)silyl)]-substituted benzenethiolate.[17]

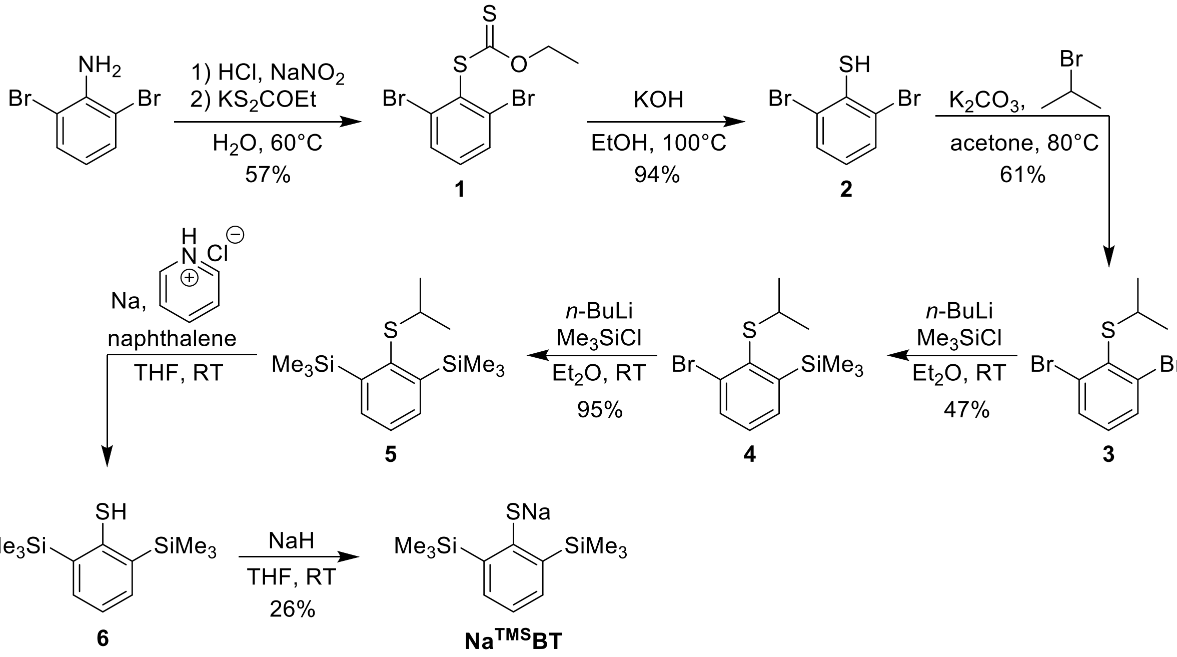

Supporting Fig. 1. Synthetic route for ligand **Na^{TMS}BT**.

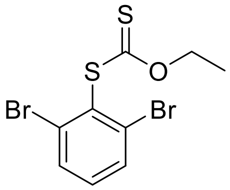

(2,6-dibromophenyl)ethyl xanthate (1). In a Schlenk flask, 2,6-dibromoaniline (24.50 g, 97.6 mmol, 1.0 equiv.) was dissolved in 45 mL of water and concentrated HCl (16.3 mL, 15.7 mmol, 0.2 equiv.) together with 15 g ice were added. Sodium nitrite (6.74 g, 97.6 mmol, 1.0 equiv.) was dissolved in 25 mL water and added over 40 mins to the sludgy white suspension of the aniline. The resulting yellow reaction mixture was stirred for another 30 mins at 0°C. Potassium ethyl xanthate (18.77 g, 117.2 mmol, 1.2 equiv.) was dissolved in 100 mL water at 60 °C. The diazo compound formed in the previous step was added dropwise over 20 minutes to the xanthate solution at 60°. After the addition, the reaction mixture was stirred for 40 minutes at 60°C and then cooled to room temperature, providing an orange suspension. Then, the reaction mixture was filtered, and the obtained solid was washed with cold methanol and pentane and then dried under vacuum to give an off-white powder of **1** (20.18 g, 56.7 mmol, 57%). ^{1}H NMR ($CDCl_3$, 400 MHz, 298 K): δ [ppm] = 7.68 (d, $^3J(^1H,^1H)$ = 8.0 Hz, 2H, $H^{3,5}$), 7.14 (t, $^3J(^1H,^1H)$ = 8.0 Hz, 1H, H^4), 4.62 (q, $^3J(^1H,^1H)$ = 7.1 Hz, 2H, -OCH_2CH$_3$), 1.33 (t,

$^3J(^1H,^1H)$ = 7.1 Hz, 3H, -OCH_2CH_3). Analytical data are in accordance with the literature-reported ones.[17]

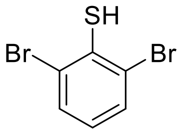

2,6-dibromothiophenol (2). In a round-bottom flask, compound **1** (20.17 g, 56.7 mmol, 1.0 equiv.) and potassium hydroxide (95 g, 7.70 mol, 30 equiv.) were mixed with 200 mL of ethanol, and the suspension was stirred at 100°C for 7.5 h and then for 14 h at room temperature resulting in a thick orange mixture. The ethanol was evaporated under vacuum, yielding a brown solid, which was mixed with water (300 mL) and washed with diethyl ether. The aqueous phase was cooled to 0°C and the mixture was acidified with HCl (12 M) and stirred at 0 °C for another 15 min (pH = 1), resulting in a white cloudy solution with orange solid, which was extracted with diethyl ether (3 × 100 mL). The organic layers were combined, dried over $MgSO_4$, filtered, and concentrated to dryness, yielding an orange solid of **2** (14.31 g, 53.4 mmol, 94%). ^1H NMR ($CDCl_3$, 400 MHz, 298 K): δ [ppm] = 7.48 (d, $^3J(^1H,^1H)$ = 8.0 Hz, 2H, $H^{3,5}$), 6.82 (t, $^3J(^1H,^1H)$ = 8.0 Hz, 1H, H^4), 5.00 (s, 1H, -S*H*). Analytical data are in accordance with the literature-reported ones.[17]

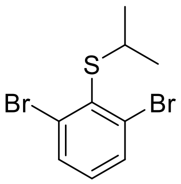

(2,6-dibromophenyl)isopropyl thioether (3). In a round-bottom flask connected to a reflux condenser, compound **2** (14.31 g, 53.4 mmol, 1.0 equiv.), 2-bromopropane (7.88 g, 64.1 mmol, 6.0 mL, 1.2 equiv.) and potassium carbonate (11.81 g, 85.4 mmol, 1.6 equiv.) were mixed with 100 mL of acetone and stirred under reflux (80 °C) for 7 h, and then at room temperature overnight, resulting in a brown cloudy suspension. The acetone was evaporated under a vacuum, and the solid residue was taken up in 200 mL of CH_2Cl_2. The solution was washed with brine. The deep red organic layer was dried over $MgSO_4$, filtered, and dried under a vacuum. The resulting oil was stirred in 60 mL of degassed and dry pentane at -50 °C for 30 mins under an argon atmosphere, yielding an orange precipitate. After sedimentation, the pentane was filtered off, and the remaining solid was dried under vacuum to give a pale orange solid of **3** (10.07 g, 32.5 mmol, 61%). ^1H NMR ($CDCl_3$, 400 MHz, 298 K): δ [ppm] = 7.62 (d, $^3J(^1H,^1H)$ = 8.0, 2H, $H^{3,5}$), 6.99 (t, $^3J(^1H,^1H)$ = 8.0, 1H, H^4), 3.59 (sept, $^3J(^1H,^1H)$ = 6.7, 1H, H^7), 1.29 (d, $^3J(^1H,^1H)$ = 6.4, 6H, $H^{8,8'}$). Analytical data are in accordance with the literature-reported ones.[17]

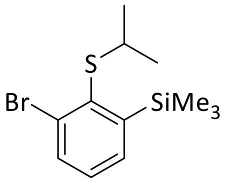

(2-bromo-6-trimethylsilylphenyl)isopropyl thioether (4). In a dry Schlenk flask, compound **3** (10.07 g, 32.5 mmol, 1.0 equiv.) was dissolved in 40 mL of diethyl ether under an argon atmosphere. The yellow solution was cooled to -78 °C, and *n*-BuLi (2.5 M in hexane, 14.3 mL, 35.7 mmol, 1.1 equiv.) was added dropwise. The cooling bath was removed, and the reaction mixture was stirred for 1 h at room temperature. Then, the reaction mixture was cooled again to -78 °C, and Me_3SiCl (3.64 g, 33.5 mmol, 1.03 equiv.) was slowly added via syringe. The reaction mixture was stirred for 24 h at room temperature to give a yellow suspension, which was evaporated to dryness. The solid residue was mixed with toluene (~40 mL), and the obtained suspension was filtrated to a new Schlenk tube. Toluene was removed under vacuum, and the resulting orange oil was distilled under high vacuum (0.05 mbar, 140 ºC) to give clear, colorless oil of **4** (6.22 g, 20.5 mmol, 63%). ^{1}H NMR ($CDCl_3$, 400 MHz, 298 K): δ [ppm] = 7.63 (d, $^3J(^1H,^1H)$ = 8.0 Hz, 1H, H^5), 7.44 (d, $^3J(^1H,^1H)$ = 8.0 Hz, 1H, H^3), 7.11 (t, $^3J(^1H,^1H)$ = 8.0 Hz, 1H, H^4), 3.84 (sept, $^3J(^1H,^1H)$ = 7.0 Hz, 1H, H^7), 1.21 (d, $^3J(^1H,^1H)$ = 7.0 Hz, 6H, $H^{8,8'}$), 0.39 (s, 9H, -Si(CH_3)$_3$). $^{13}C\{^1H\}$ NMR ($CDCl_3$, 101 MHz, 298 K): δ [ppm] = 149.7 (s, 1C, C^6), 141.2 (s, 1C, C^1), 134.5 (s, 1C, C^5), 134.0 (s, 1C, C^3), 132.0 (s, 1C, C^2), 128.8 (s, 1C, C^4), 38.9 (s, 1C, C^7), 22.7 (s, 2C, $C^{8,8'}$), 1.2 (s, 1C, -Si(CH$_3$)$_3$). ^{29}Si NMR ($CDCl_3$, 119 MHz, 298 K): δ [ppm] = -3.20 (-Si(CH$_3$)$_3$).

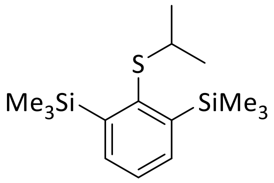

(2,6-ditrimethylsilylphenyl)isopropyl thioether (5). In a dry Schlenk tube, compound **4** (1.46 g, 4.7 mmol, 1.0 equiv.) was dissolved in 30 mL of dry diethyl ether. The yellow solution was cooled to -78 °C, and *n*-BuLi (2.5 M in hexane, 1.2 mL, 5.2 mmol, 1.1 equiv.) was added dropwise. The cooling bath was removed, and the reaction mixture was stirred for 1 h at room temperature. Then, the clear yellow solution was cooled again to -78 °C and Me_3SiCl (538 mg, 5.0 mmol, 1.05 equiv.) was slowly added via syringe. The reaction mixture was stirred for 24 h to give a cloudy yellow mixture. The ether was removed under vacuum, and the solid residue was mixed with 50 mL of toluene. The white precipitate was filtered off, and the toluene was removed under a vacuum. The resulting yellow oil was dried under vacuum and purified via column chromatography in *n*-hexane:ethyl acetate = 100:1 to give a clear, colorless oil of **5** (1.36 g, 4.6 mmol, 95%, R_f = 0.8). ^{1}H NMR (C_6D_6, 400 MHz, 298 K): δ [ppm] = 7.53 (d, $^3J(^1H,^1H)$ = 7.0 Hz, 2H, $H^{3,5}$), 7.12 (t, $^3J(^1H,^1H)$ = 7.0 Hz, 1H, H^4), 3.59 (sept, $^3J(^1H,^1H)$ = 7.0 Hz, 1H, H^7), 1.02 (d, $^3J(^1H,^1H)$ = 7.0 Hz, 6H, $H^{8,8'}$), 0.44 (s, 18H, -Si(CH_3)$_3$). $^{13}C\{^1H\}$ NMR (C_6D_6, 100 MHz, 298 K): δ [ppm] = 148.8 (s, 1C, C^1), 147.7 (s, 2C, $C^{2,6}$), 136.8 (s, 2C, $C^{3,5}$), 126.8 (s, 1C, C^4), 42.5 (s, 1C, C^7), 22.4 (s, 2C, $C^{8,8'}$), 1.8 (-Si(CH$_3$)$_3$). ^{29}Si NMR (C_6D_6, 79 MHz, 298 K): δ [ppm] = -4.53 (-Si(CH$_3$)$_3$).

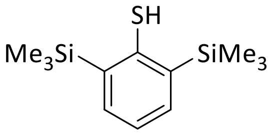

(2,6-ditrimethylsilyl)thiophenol (6). Under an argon atmosphere, compound **5** (1.01 g, 3.39 mmol, 1.0 equiv.) was dissolved in 50 mL THF. Then, sodium (203 mg, 8.8 mmol, 2.6 equiv.) and naphthalene (552 mg, 4.1 mmol, 1.2 equiv.) were added portion-wise. The reaction mixture was stirred at room temperature for 24 h. After that, the deep-blue solution was cooled to −80°C and pyridinium chloride (1.02 g, 8.8 mmol, 2.6 equiv.) was added. The reaction mixture was slowly warmed up to RT and stirred overnight at room temperature, resulting in a grey suspension. The THF was removed under vacuum, and the solid residue vacuum dried. The solid residue was mixed with toluene (50 mL) and stirred for 15 minutes. Then, the solution was filtered, and the solvent was removed under vacuum to give an orange oil of **6**, which was used in the next reaction without further purification and characterization.

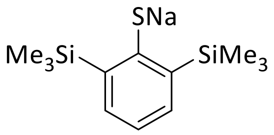

Sodium 2,6-bis(trimethylsilyl)benzenethiolate (**Na^{TMS}BT**). Under an argon atmosphere, sodium hydride (140 mg, 5.81 mmol, 1.2 equiv.) was suspended in THF (5 mL), and crude compound **6** (1.23 g), dissolved in 5 mL of THF, was added portion-wise via syringe. After stirring the reaction mixture at room temperature for 5 h, during which evolution of gaseous hydrogen was observed, the excess of NaH was filtered off, and the solvent was evaporated under vacuum to dryness. The obtained yellow solid was washed with *n*-pentane (2 × 10 mL) and dried under vacuum to give white powder of **Na^{TMS}BT**. (774 mg, 2.8 mmol, 57%). ^{1}H NMR (C_4D_8O, 400 MHz, 298 K): δ [ppm] = 7.0 (d, $^{3}J(^{1}H,^{1}H)$ = 7.2 Hz, 2H, $H^{3,5}$), 6.51 (t, $^{3}J(^{1}H,^{1}H)$ = 7.2 Hz, 1H, H^{4}), 0.34 (s, 18H, -Si(CH_3)$_3$). ^{13}C{^{1}H} NMR (C_4D_8O, 100 MHz, 298 K): δ [ppm] = 172.0 (s, 1C, C^{1}), 139.7 (s, 2C, $C^{2,6}$), 133.9 (s, 2C, $C^{3,5}$), 116.6 (s, 1C, C^{4}), 0.2 (s, 6C, -Si(CH_3)$_3$). ^{29}Si NMR (C_4D_8O, 80 MHz, 298 K): δ [ppm] = -10.50 (s, 2Si, -*Si*(CH_3)$_3$).

1,10-phenanthroline-5-carbonitrile (^{CN}phen)

^{CN}phen was prepared by the literature-reported procedure using the nucleophilic attack of CN^- to 1,10-phenanthroline-5,6-epoxide (Supplementary Fig. 2).[18]

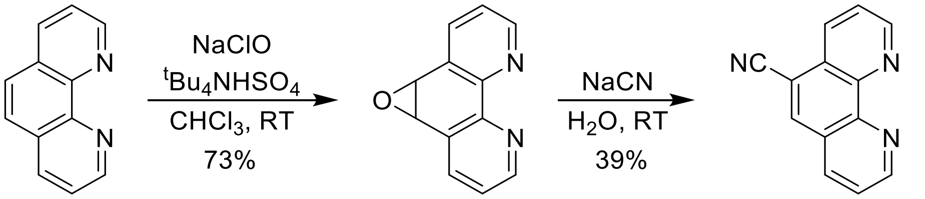

Supplementary Fig. 2. Synthetic route for ligand ^{CN}phen.

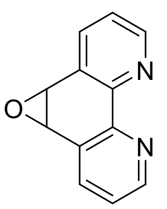

5,6-epoxy-5,6-dihydro-1,10-phenanthroline ($^{\mathbf{O}}$phen). 1,10-phenanthroline (8.0 g, 40 mmol, 1.0 equiv.) was dissolved in 80 mL chloroform. Sodium hypochlorite (10-15% in water, 400 mL, ~500 mmol, ~13 equiv.) was cooled to 0° and acidified with nitric acid (pH ~ 8). Immediately after the desired pH was reached, tetra-test-butylammonium bisulfate (7.2 g, 21 mmol, 0.5 equiv.) and the 1,10-phenanthroline solution were added. The mixture was stirred for 25 minutes, followed by extraction with chloroform (3 × 70 mL). The combined organic phases were washed with basic water (pH = 8), water, and brine, dried over $MgSO_4$, and evaporated to dryness under a vacuum. The resulting powder was dissolved in 80 mL chloroform, and *n*-pentane was added dropwise until a white solid precipitated. Then, the mixture was cooled to -78°C and stirred for 30 min. The solution was filtered off, and the solid was washed with *n*-pentane and dried under vacuum to give a white powder of $^{\mathbf{O}}$**phen** (5.70 g, 291 mmol, 73%). ^{1}H NMR ($CDCl_3$, 400 MHz, 298 K): δ [ppm] = 8.89 (dd, $^3J(^1H,^1H)$ = 4.7, 1.7 Hz, 2H, $H^{2,9}$), 7.99 (dd, $^3J(^1H,^1H)$ = 7.6, 1.7 Hz, 2H, $H^{4,7}$), 7.38 (dd, $^3J(^1H,^1H)$ = 7.7, 4.7 Hz, 2H, $H^{3,8}$), 4.60 (s, 2H, $H^{5,6}$) ppm. Analytical data are in accordance with the literature-reported ones.[19]

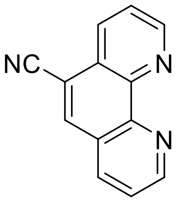

5-cyano-1,10-phenanthroline ($^{\mathbf{CN}}$phen). $^{\mathbf{O}}$**phen** (2.0 g, 10 mmol, 1.0 equiv.) and sodium cyanide (2.5 g, 50 mmol, 5.0 equiv.) were dissolved in 200 mL of water each. The obtained solutions were mixed and stirred at room temperature overnight. After that, the solvent was filtered off, and the solid residue was washed with water (3 × 70 mL) and dried under a high vacuum for 24 h, to give a white powder of $^{\mathbf{CN}}$**phen** (792 mg, 3.9 mmol, 39%). ^{1}H NMR (C_4D_8O, 600 MHz, 298 K): δ [ppm] = 9.22 (m, 2H, $H^{2,9}$), 8.61 (dd, $^3J(^1H,^1H)$ = 8.1 Hz, $^4J(^1H,^1H)$ = 1.7 Hz, 1H, H^4), 8.56 (s, 1H, H^6), 8.44 (dd, $^3J(^1H,^1H)$ = 8.1 Hz, $^4J(^1H,^1H)$ = 1.7 Hz, 1H, H^7), 7.83 (dd, $^3J(^1H,^1H)$ = 8.1 Hz, $^3J(^1H,^1H)$ = 4.2 Hz, 1H, H^3), 7.75 (dd, $^3J(^1H,^1H)$ = 8.1 Hz, $^3J(^1H,^1H)$ = 4.2 Hz, 1H, H^8). $^{13}C\{^1H\}$ NMR (C_4D_8O, 150 MHz, 298 K): δ [ppm] = 153.5 (1C, C^9), 152.0 (1C, C^2), 148.5 (1C, C^{10a}), 146.9 (1C, C^{10b}), 137.3 (1C, C^7), 136.1 (1C, C^6), 133.7 (1C, C^4), 127.9 (1C, C^{6a}), 127.0 (1C, C^{4a}), 124.7 (1C, C^3), 124.5 (1C, C^8), 116.8 (1C, C^{CN}), 110.1 (1C, C^5). ^{15}N

(C_4D_8O, 61 MHz, 298 K): δ [ppm] = -66.2 (2N, $N^{1,10}$). Analytical data are in accordance with the literature-reported ones.[18]

Na1D

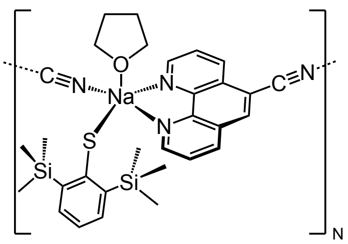

Na1D

Under an argon atmosphere, ^{CN}phen (74.2 mg, 362 mmol, 1 equiv.) and Na^{TMS}BT (100 mg, 362 mmol, 1 equiv.) were mixed in 10 mL of THF, leading to a brisk color change from white to orange-red. The mixture was stirred overnight at room temperature. After that, a small amount of insoluble material was filtered off, and crystallization was induced by gas-phase diffusion of *n*-pentane as an anti-solvent to reach a ratio of *n*-pentane:THF of ca. 1:1. Importantly, diffusion of an increased amount of n-pentane resulted in minor contamination of the sample with red crystals of **Na2D** assembly. The obtained yellow single crystals were collected, washed with toluene (5 mL) and *n*-pentane (3 × 5 mL) and dried under vacuum to give orange single crystals of **Na1D** (77.5 mg, 140 mmol, 39%). ^{1}H NMR (C_4D_8O, 400 MHz, 298 K): δ [ppm] = 9.12 (d, $^3J(^1H,^1H)$ = 4.2 Hz, 2H, $H^{2,9}$, ^{CN}phen), 8.68 (d, $^3J(^1H,^1H)$ = 8.2 Hz, 1H, H^4, ^{CN}phen), 8.61 (s, 1H, H^6, ^{CN}phen), 8.51 (d, $^3J(^1H,^1H)$ = 8.1 Hz, 1H, H^7, ^{CN}phen), 7.84 (dd, $^3J(^1H,^1H)$ = 4.4, 8.2 Hz, 1H, H^3, ^{CN}phen), 7.74 (dd, $^3J(^1H,^1H)$ = 8.1, 4.4 Hz, 1H, H^8, ^{CN}phen), 7.03 (d, $^3J(^1H,^1H)$ = 7.2 Hz, 2H, $H^{3,5}$, ^{TMS}BT), 6.56 (t, $^3J(^1H,^1H)$ = 7.2 Hz, 1H, H^4, ^{TMS}BT), 3.61 (m, 4H, THF), 1.77 (m, 4H, THF), 0.28 (s, 18H, -Si(C*H*$_3$)$_3$, ^{TMS}BT). ^{13}C{^{1}H} NMR (C_4D_8O, 125 MHz, 298 K): δ [ppm] = 174.1 (s, 1C, C^1, ^{TMS}*BT*), 154.2 (s, 1C, C^9, ^{CN}phen), 152.6 (s, 1C, C^2, ^{CN}phen), 147.6 (s, 1C, C^{6a}, ^{CN}phen), 146.1 (s, 1C, C^{4a}, ^{CN}phen), 140.8 (s, 2C, $C^{2,6}$, ^{TMS}BT), 138.1 (s, 1C, C^7, ^{CN}phen), 136.1 (s, 1C, C^6, ^{CN}phen), 135.0 (s, 2C, $C^{3,5}$, ^{TMS}BT), 134.6 (s, 1C, C^4, ^{CN}phen), 128.0 (s, 1C, $C^{10a/11a}$, ^{CN}phen), 127.2 (s, 1C, $C^{10a/11a}$, ^{CN}phen), 125.2 (s, 1C, C^3, ^{CN}phen), 125.0 (s, 1C, C^8, ^{CN}phen), 117.6 (s, 1C, -*C*N, ^{CN}phen), 116.5 (s, 1C, C^4, ^{TMS}BT), 110.2 (s, 1C, C^5, ^{CN}phen), 68.0 (s, 2C, THF), 26.1 (s, 2C, THF), 1.1 (s, 2C, -*C*H$_3$, ^{TMS}BT). ^{15}N (C_4D_8O, 61 MHz, 298 K): δ [ppm] = -79.1 (2N, $N^{1,10}$). ^{29}Si NMR (C_4D_8O, 119 MHz, 298 K): δ [ppm] = -10.53 (s, 2Si, ^{TMS}BT). CHN analysis calcd for $C_{29}H_{36}N_3Si_2SONa\cdot(C_5H_{12})_{0.3}$: C, 63.7; H, 6.9; N, 7.3. Found: C, 63.4; H, 6.9; N, 7.4. ESI-MS m/z: pos mode: 433.1172 $[Na(C_{13}H_7N_3)_2]^+$, 228.0533 $[NaC_{13}H_7N_3]^+$, 206.0715 $[C_{13}H_8N_3]^+$; neg. mode: 301.0756 $[(S(C_6H_2(Si(CH_3)_3)_2))NaCN]^-$, 285.0807 $[SS(C_6H_3(Si(CH_3)_3)_2]^-$, 253.0908 $[S(C_6H_3(Si(CH_3)_3)_2)]^-$.

Na2D

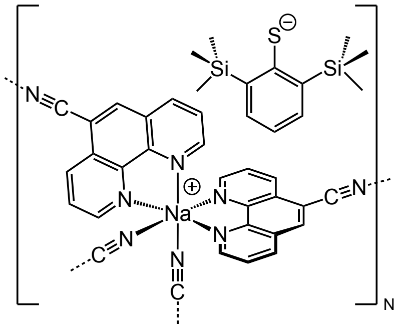

Under an argon atmosphere, ^{CN}phen (2 equiv., 148.4 mg, 724 mmol) and Na^{TMS}BT (1 equiv., 100 mg, 362 mmol) were mixed in 10 mL of THF, leading to a brisk color change from white to red. The mixture was stirred overnight at room temperature to give deep-red single crystals. The solvent was filtered off, and obtained crystals were washed with cold THF (3 × 3 mL), toluene (5 mL) and *n*-pentane (5 mL) and dried under vacuum to give red single crystalline material of **Na2D** (174.8 mg, 231 mmol, 64%). Identical single crystalline material can be obtained by gas-phase diffusion of *n*-pentane into a saturated solution of **Na2D** in THF or its layering with cyclohexane. ^{1}H NMR (C_4D_8O, 500 MHz, 298 K): δ [ppm] = 9.14 (m, 4H, $H^{2,9}$, ^{CN}phen), 8.66 (dd, $^3J(^1H,^1H)$ = 8.2 Hz, $^4J(^1H,^1H)$ = 1.6 Hz, 2H, H^4, ^{CN}phen), 8.60 (s, 2H, H^6, ^{CN}phen), 8.49 (dd, $^3J(^1H,^1H)$ = 8.2 Hz, $^4J(^1H,^1H)$ = 1.6 Hz, 2H, H^7, ^{CN}phen), 7.83 (dd, $^3J(^1H,^1H)$ = 8.2 Hz, $^3J(^1H,^1H)$ = 4.3 Hz, 2H, H^3, ^{CN}phen), 7.74 (dd, $^3J(^1H,^1H)$ = 8.2 Hz, $^3J(^1H,^1H)$ = 4.3 Hz, 2H, H^8, ^{CN}phen), 7.04 (d, $^3J(^1H,^1H)$ = 7.1 Hz, 2H, $H^{3,5}$, ^{TMS}BT), 6.58 (t, $^3J(^1H,^1H)$ = 7.1 Hz, 1H, H^4, ^{TMS}BT), 3.61 (m, 4H, THF), 1.77 (m, 4H, THF), 0.26 (s, 18H, -Si(C*H*$_3$)$_3$, ^{TMS}BT). ^{13}C{^{1}H} NMR (C_4D_8O, 150 MHz, 298 K): δ [ppm] = 174.0 (s, 1C, C^1, ^{TMS}BT), 154.0 (2C, C^9, ^{CN}phen), 152.5 (2C, C^2, ^{CN}phen), 147.9 (2C, C^{10a}, ^{CN}phen), 146.4 (2C, C^{10b}, ^{CN}phen), 140.8 (s, 2C, $C^{2,6}$, ^{TMS}BT), 137.9 (s, 2C, C^7, ^{CN}phen), 136.1 (s, 2C, C^6, ^{CN}phen), 135.1 (s, 2C, $C^{3,5}$, ^{TMS}BT), 134.4 (s, 2C, C^4, ^{CN}phen), 128.0 (2C, C^{6a}, ^{CN}phen), 127.2 (2C, C^{4a}, ^{CN}phen), 125.1 (2C, C^3, ^{CN}phen), 134.9 (2C, C^8, ^{CN}phen), 117.5 (s, 2C, C^4, ^{TMS}BT), 116.6 (s, 2C, C^{CN}, ^{CN}phen), 110.2 (s, 2C, C^5, ^{CN}phen), 68.03 (2C, $C^{2,5}$, THF), 26.19 (2C, $C^{3,4}$, THF), 1.16 (s, 6C, -Si(*C*H$_3$)$_3$). ^{29}Si NMR (C_4D_8O, 120 MHz, 298 K): δ [ppm] = -10.60 (s, 2Si, -*Si*(CH$_3$)$_3$). CHN analysis calcd for $C_{38}H_{35}N_6Si_2SNa{\cdot}C_4H_8O$: C, 66.5; H, 5.7; N, 11.1. Found: C, 66.1; H, 5.7; N, 11.0. ESI-MS m/z: pos mode: 433.1166 $[Na(C_{13}H_7N_3)_2]^+$, 228.053 $[NaC_{13}H_7N_3]^+$, 206.0712 $[C_{13}H_8N_3]^+$; neg. mode: 505.1736, $[(S(C_6H_3(Si(CH_3)_3)_2))$-$(S(C_6H_2(Si(CH_3)_3)_2))]^-$, 301.0755 $[(S(C_6H_2(Si(CH_3)_3)_2))NaCN]^-$, 285.0805 $[SS(C_6H_3(Si(CH_3)_3)_2]^-$, 253.0908 $[S(C_6H_3(Si(CH_3)_3)_2)]^-$.

NMR Spectra

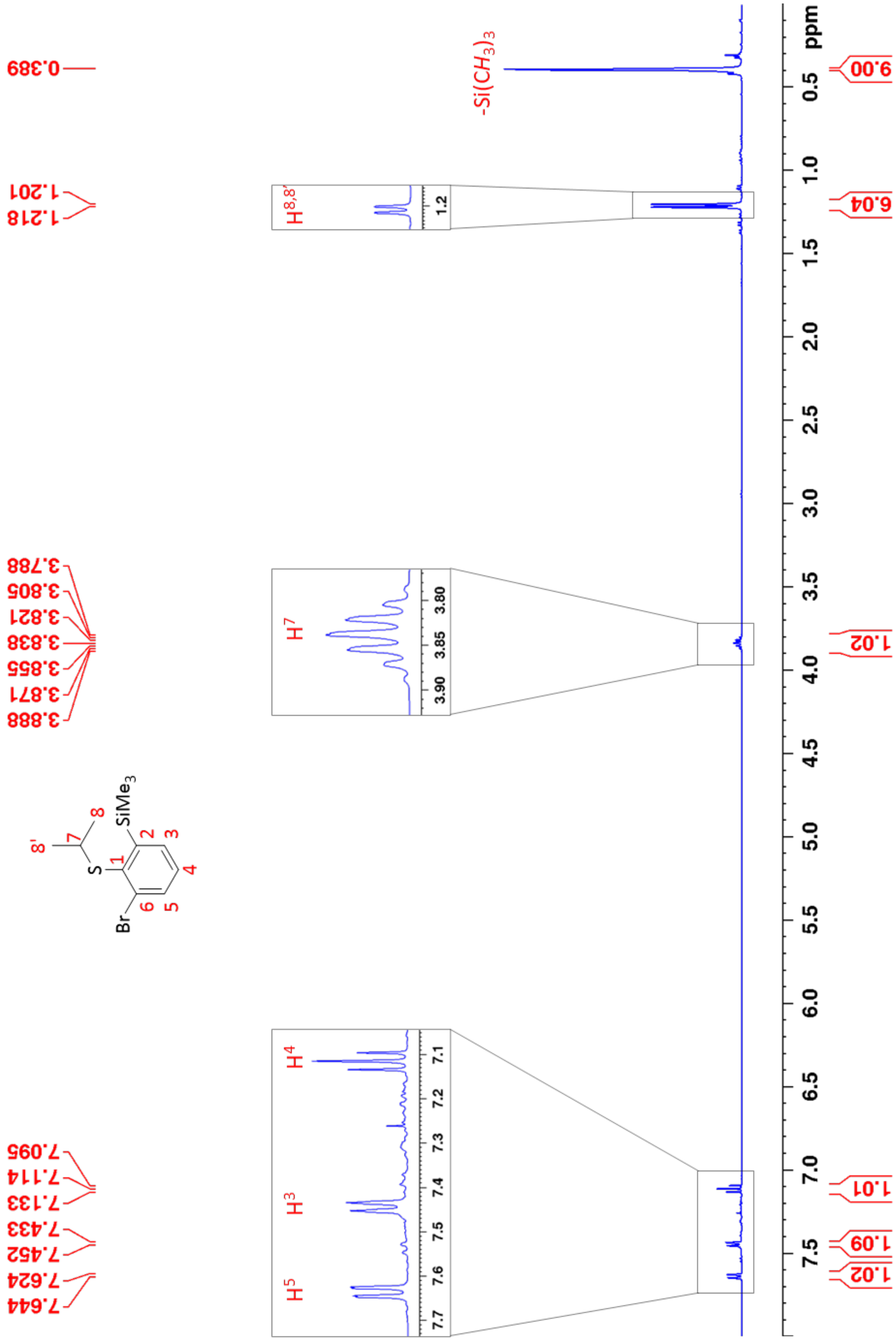

Supplementary Fig. 3. ^{1}H NMR ($CDCl_3$, 400 MHz, 298 K) of **4**.

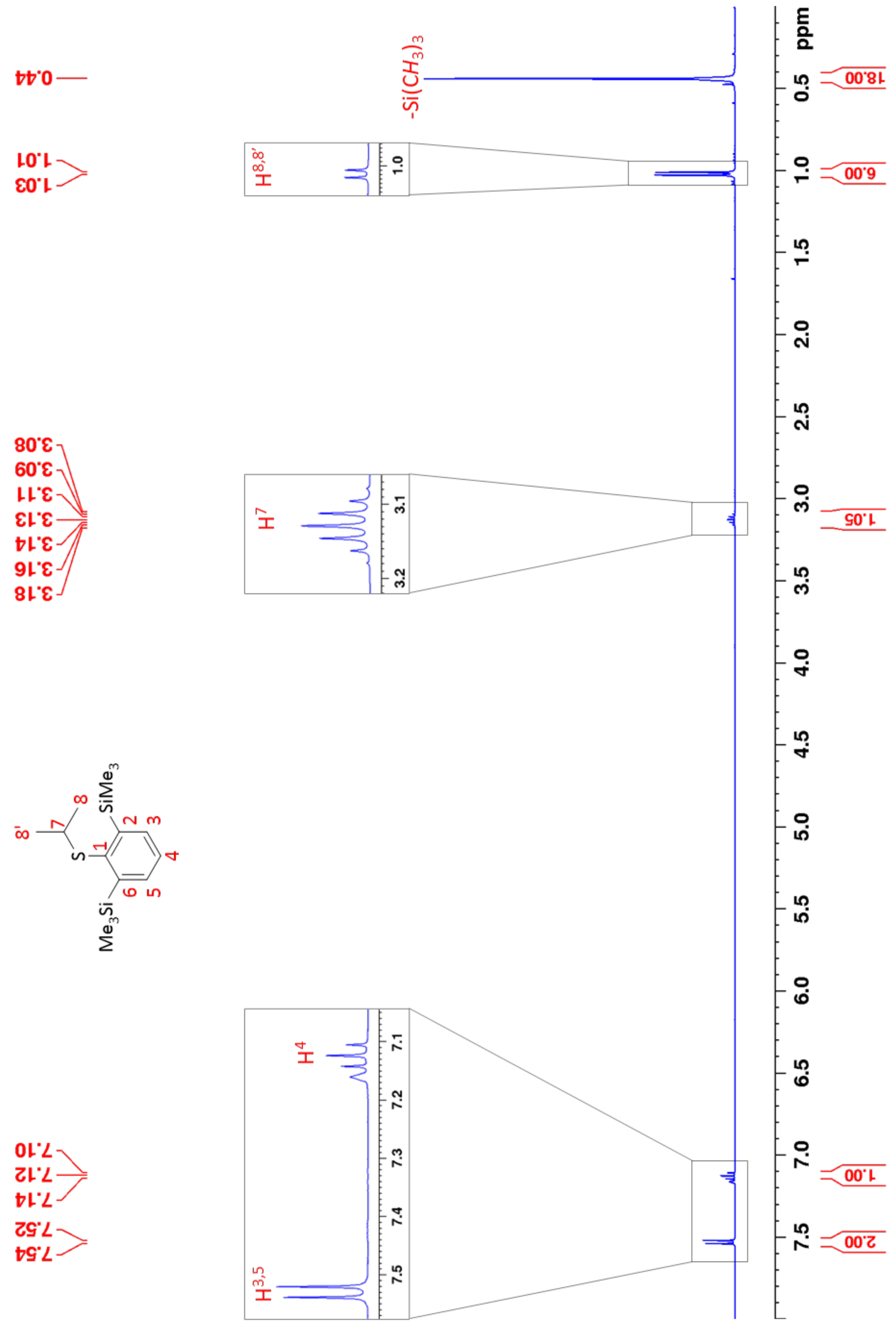

Supplementary Fig. 4. ^{1}H NMR (C_6D_6, 400 MHz, 298 K) of **5**.

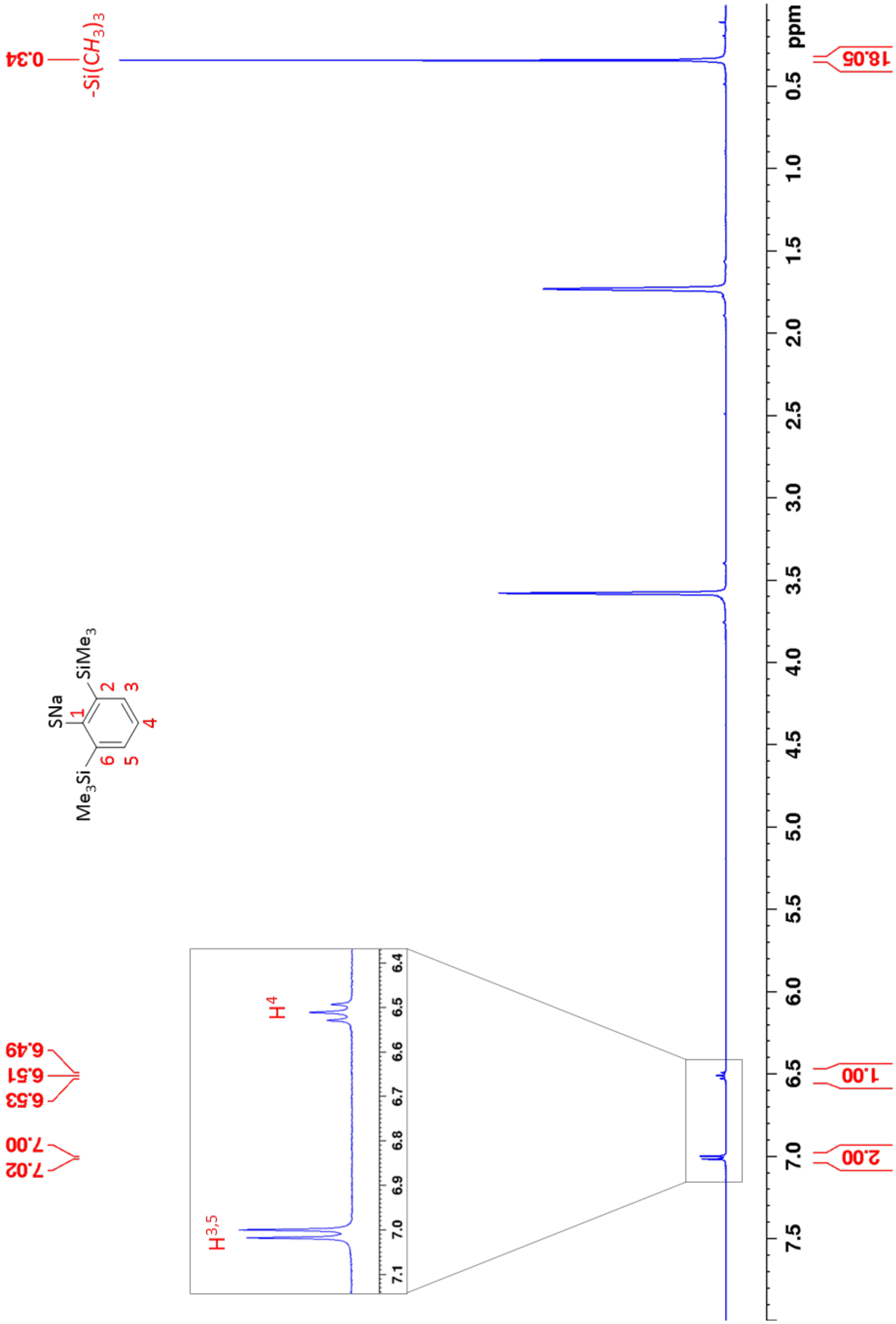

Supplementary Fig 5. ^{1}H NMR (THF-d_8, 400 MHz, 298 K) of **Na^{TMS}BT**.

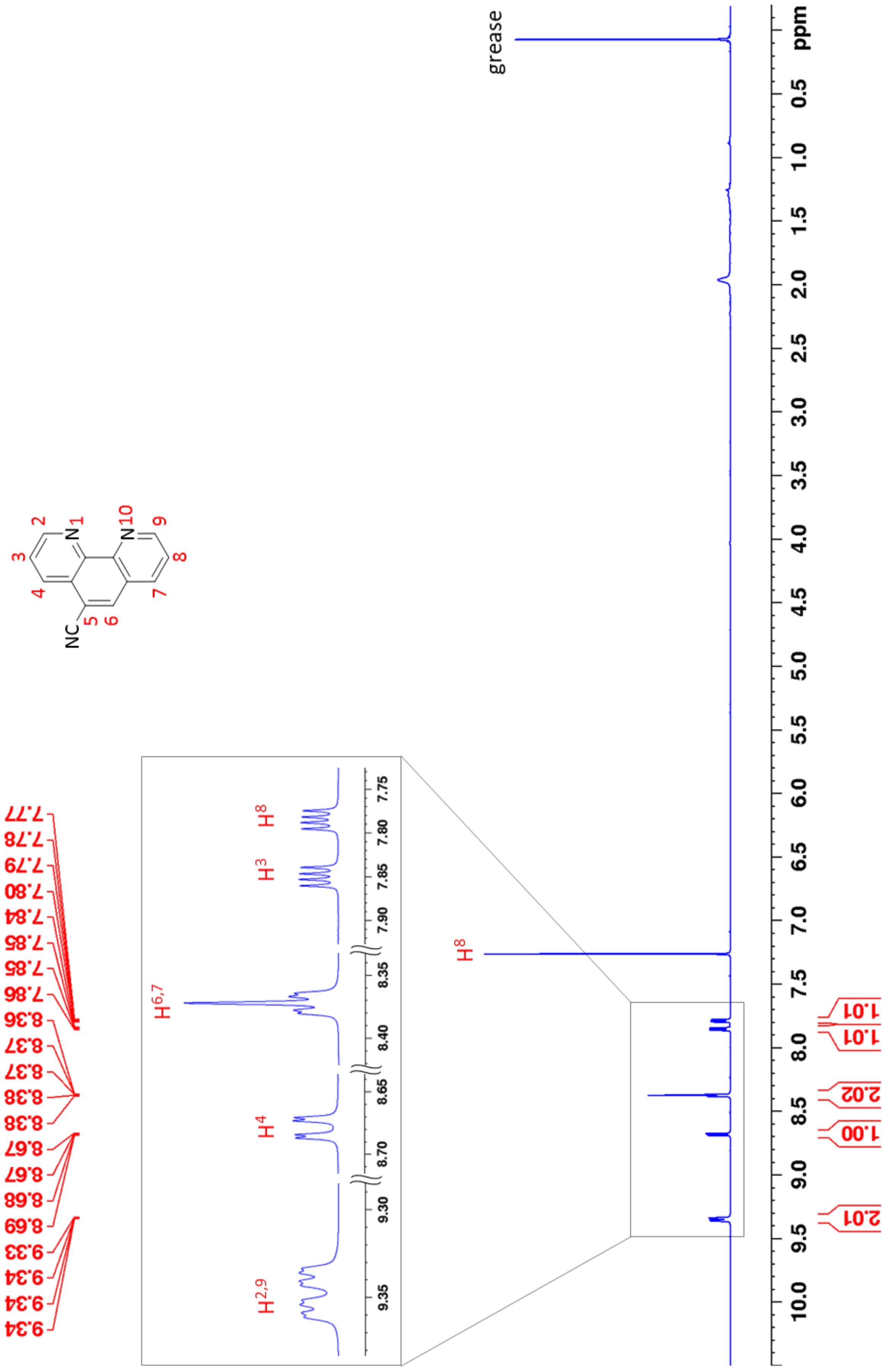

Supplementary Fig 6. ^{1}H NMR ($CDCl_3$, 400 MHz, 298 K) of ^{CN}**phen**.

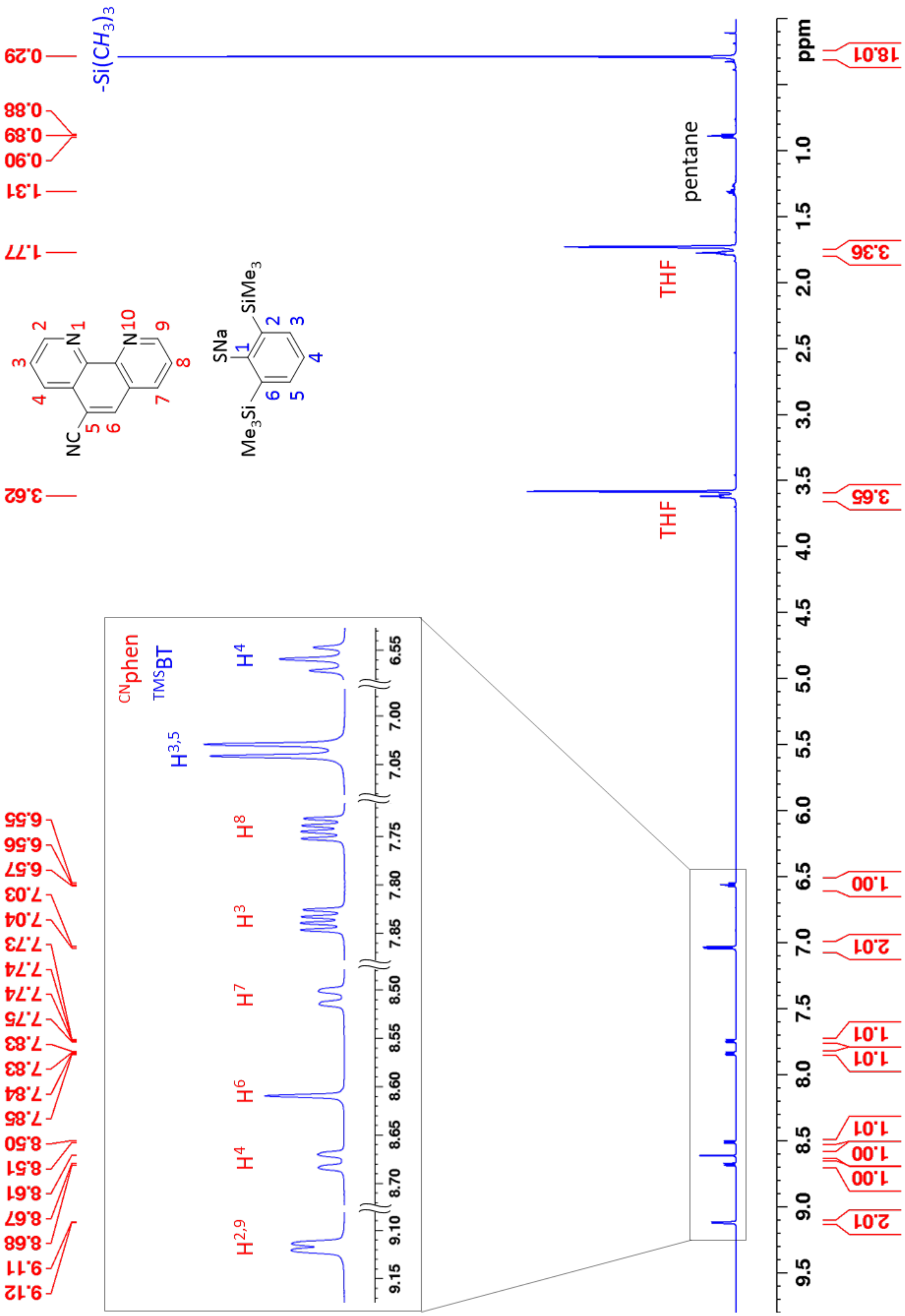

Supplementary Fig. 7. ^{1}H NMR (THF-d$_8$, 400 MHz, 298 K) of **Na1D**.

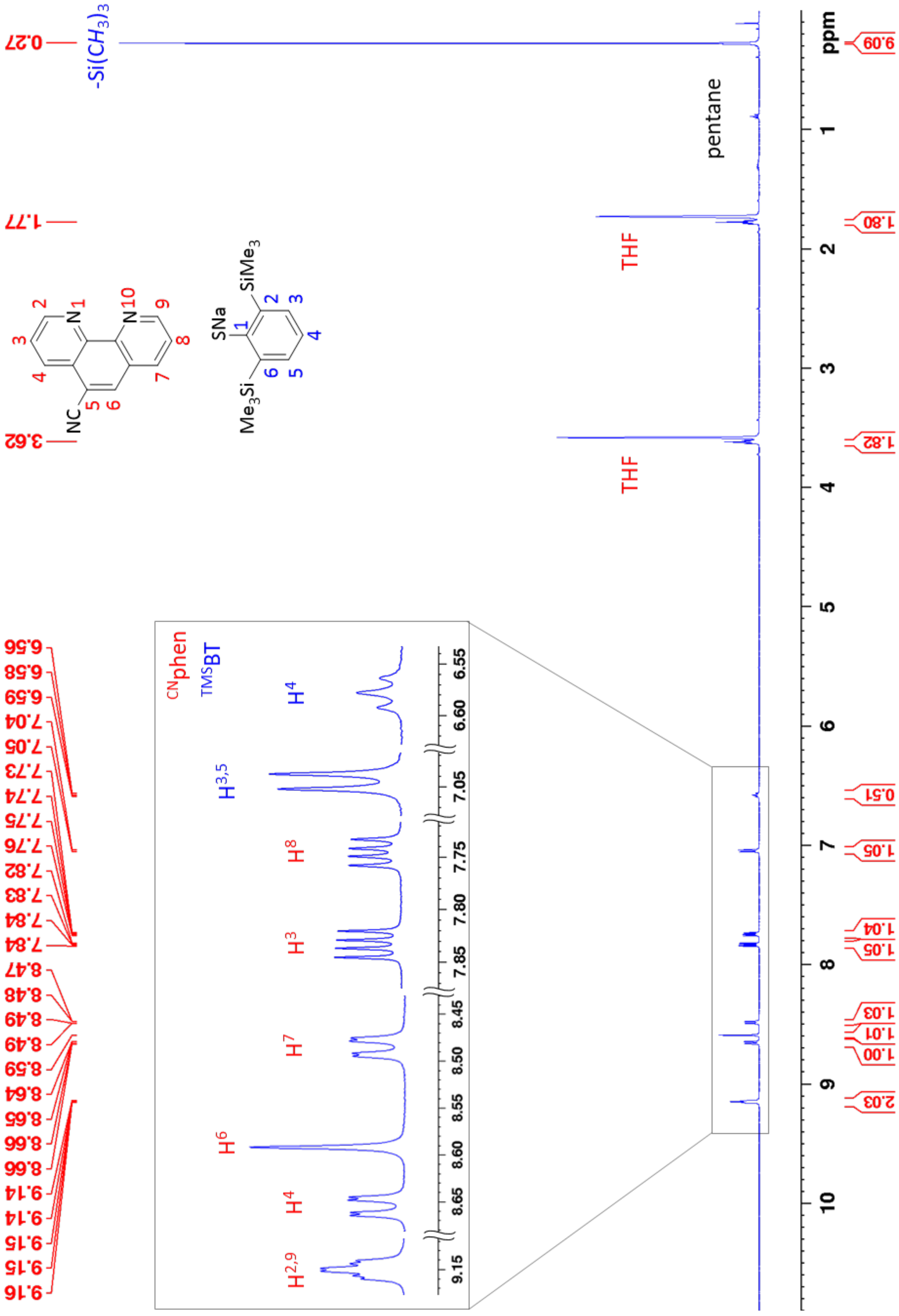

Supplementary Fig. 8. ^{1}H NMR (THF-d_8, 400 MHz, 298 K) of **Na2D.**

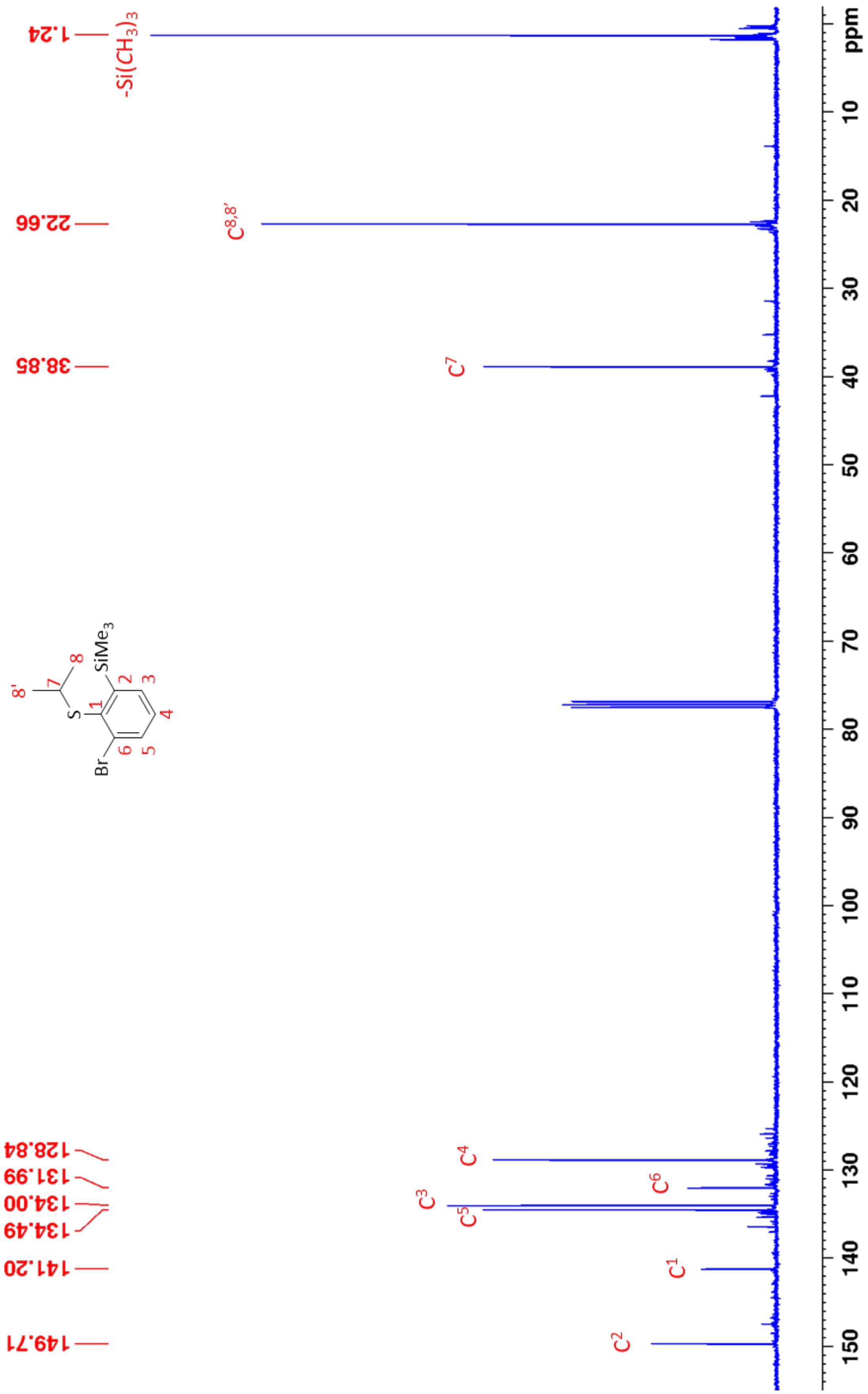

Supplementary Fig. 9. $^{13}C\{^1H\}$ NMR ($CDCl_3$, 101 MHz, 298 K) of **4**.

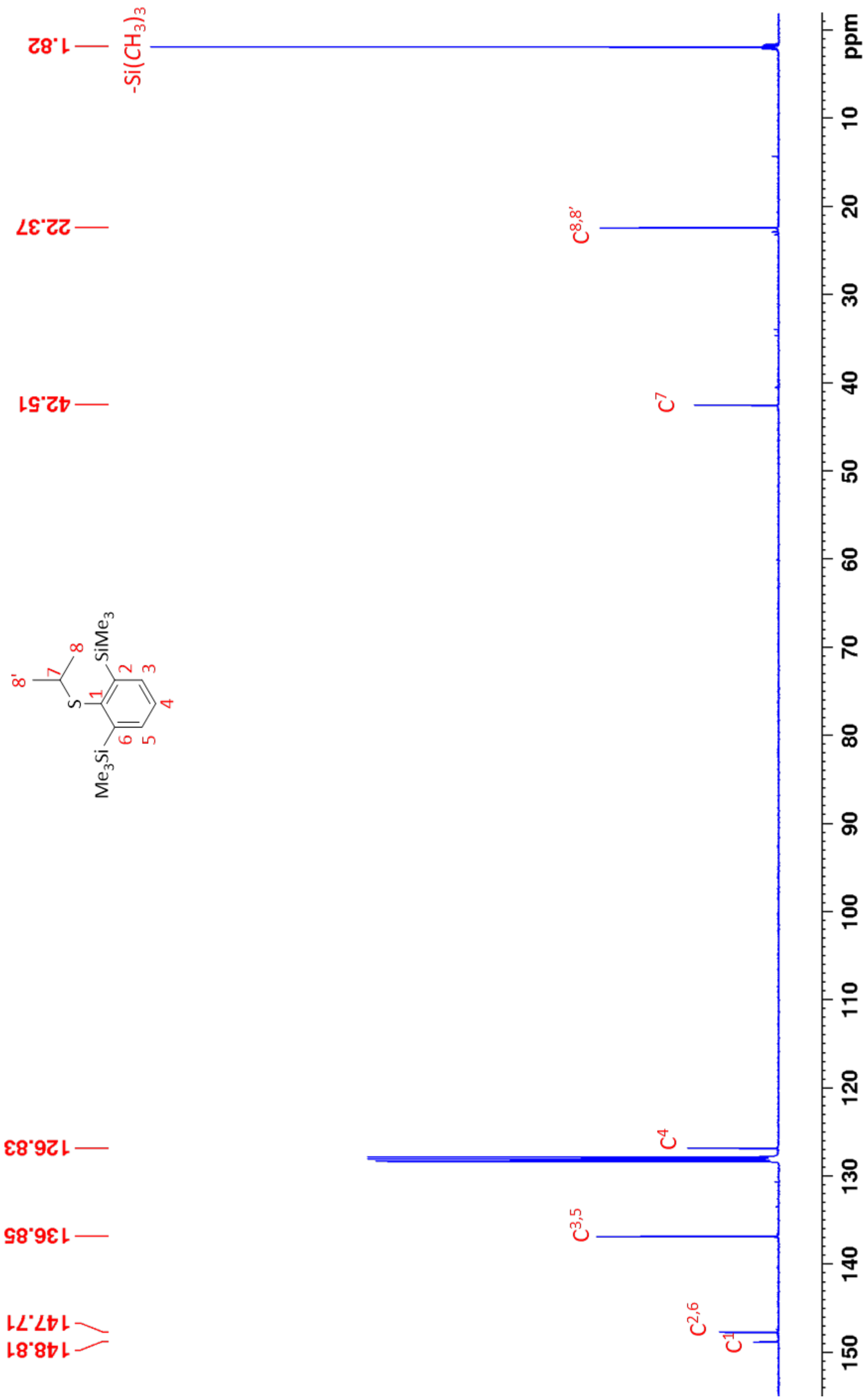

Supplementary Fig. 10. $^{13}C\{^{1}H\}$ NMR (C_6D_6, 101 MHz, 298 K) of **5**.

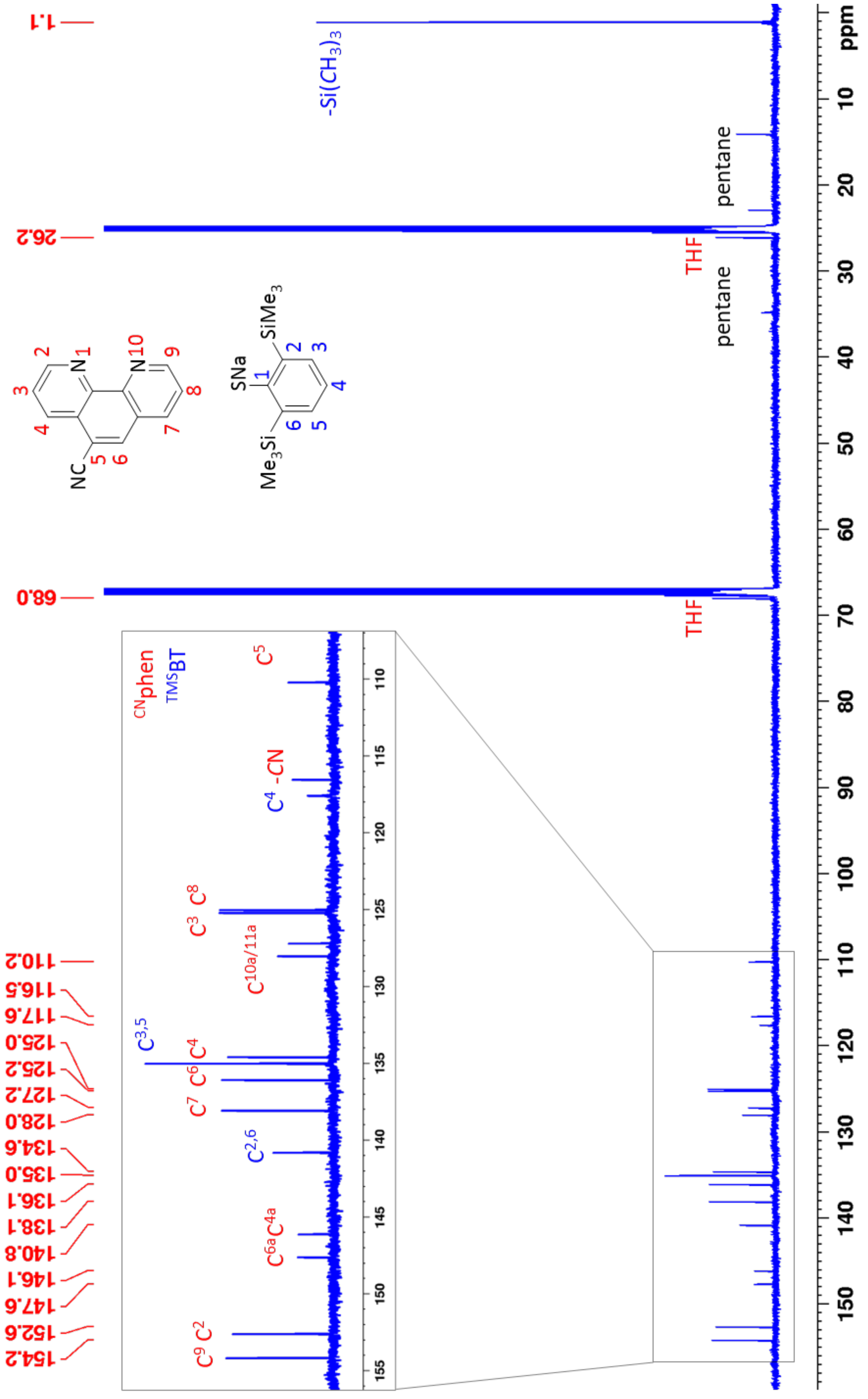

Supplementary Fig. 11. $^{13}C\{^1H\}$ NMR (THF-d_8, 125 MHz, 298 K) of **Na1D**.

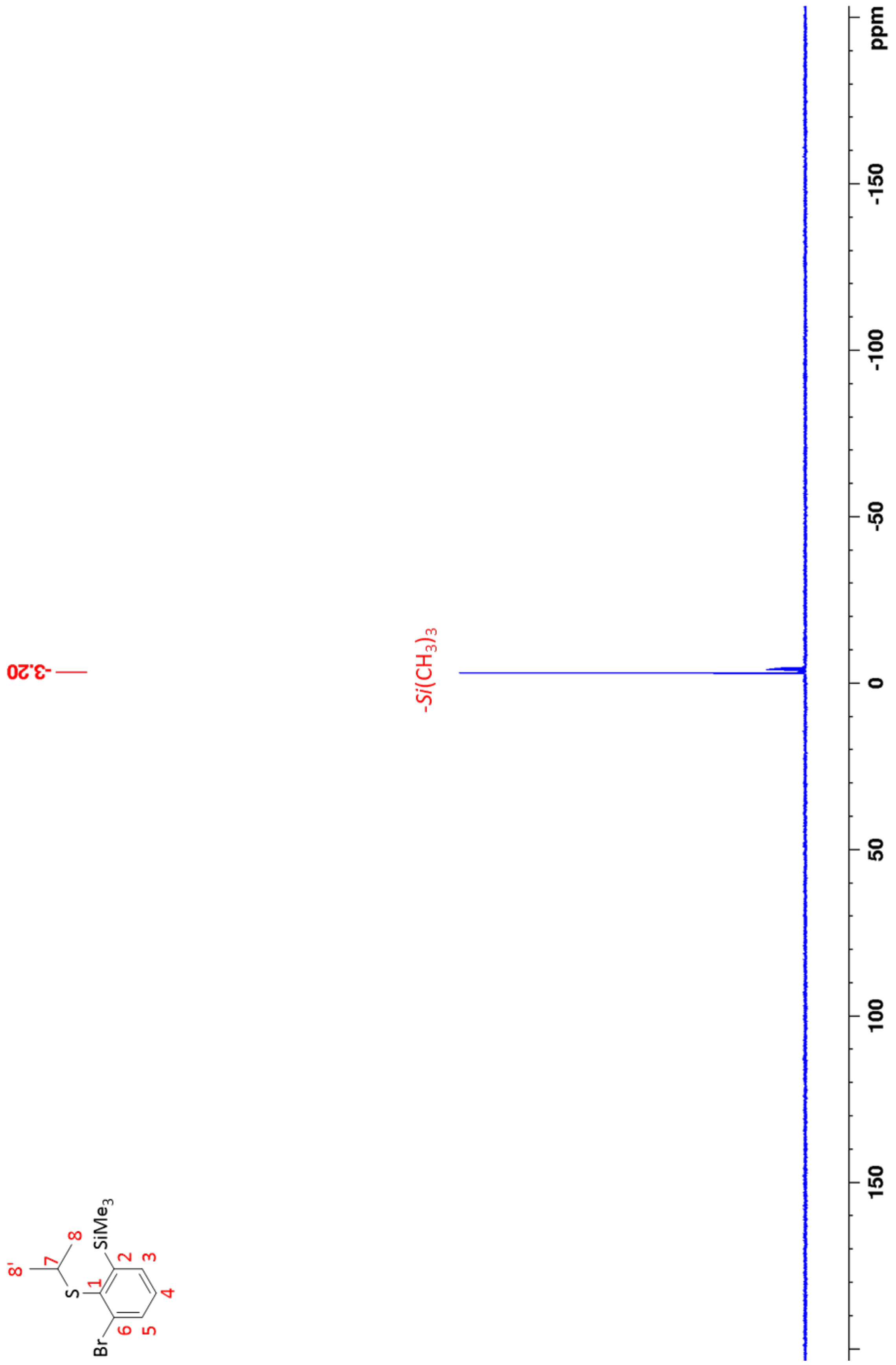

Supplementary Fig. 12. ^{29}Si NMR ($CDCl_3$, 119 MHz, 298 K) of **4**.

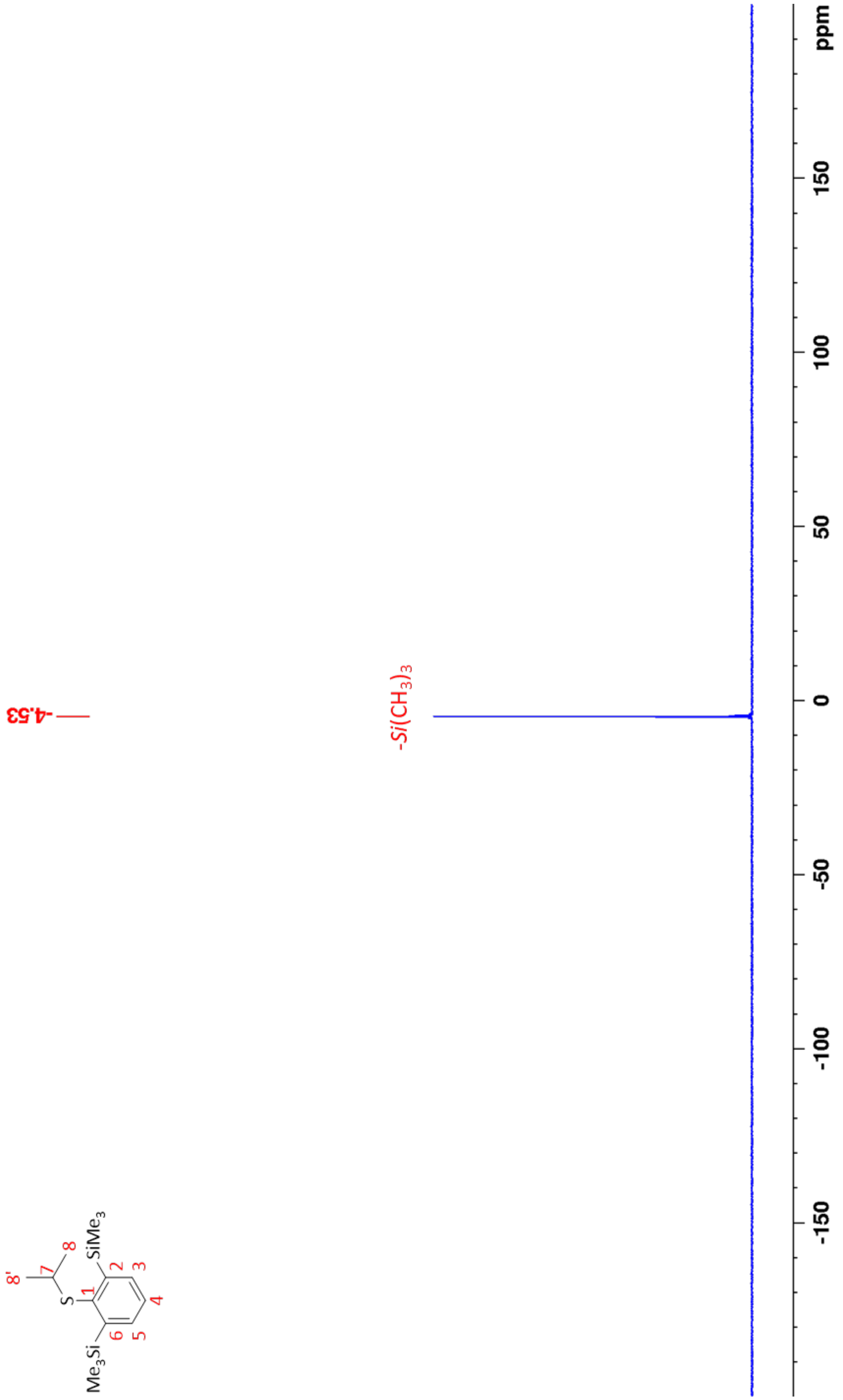

Supplementary Fig. 13. ^{29}Si NMR (C_6D_6, 79 MHz, 298 K) of **5**.

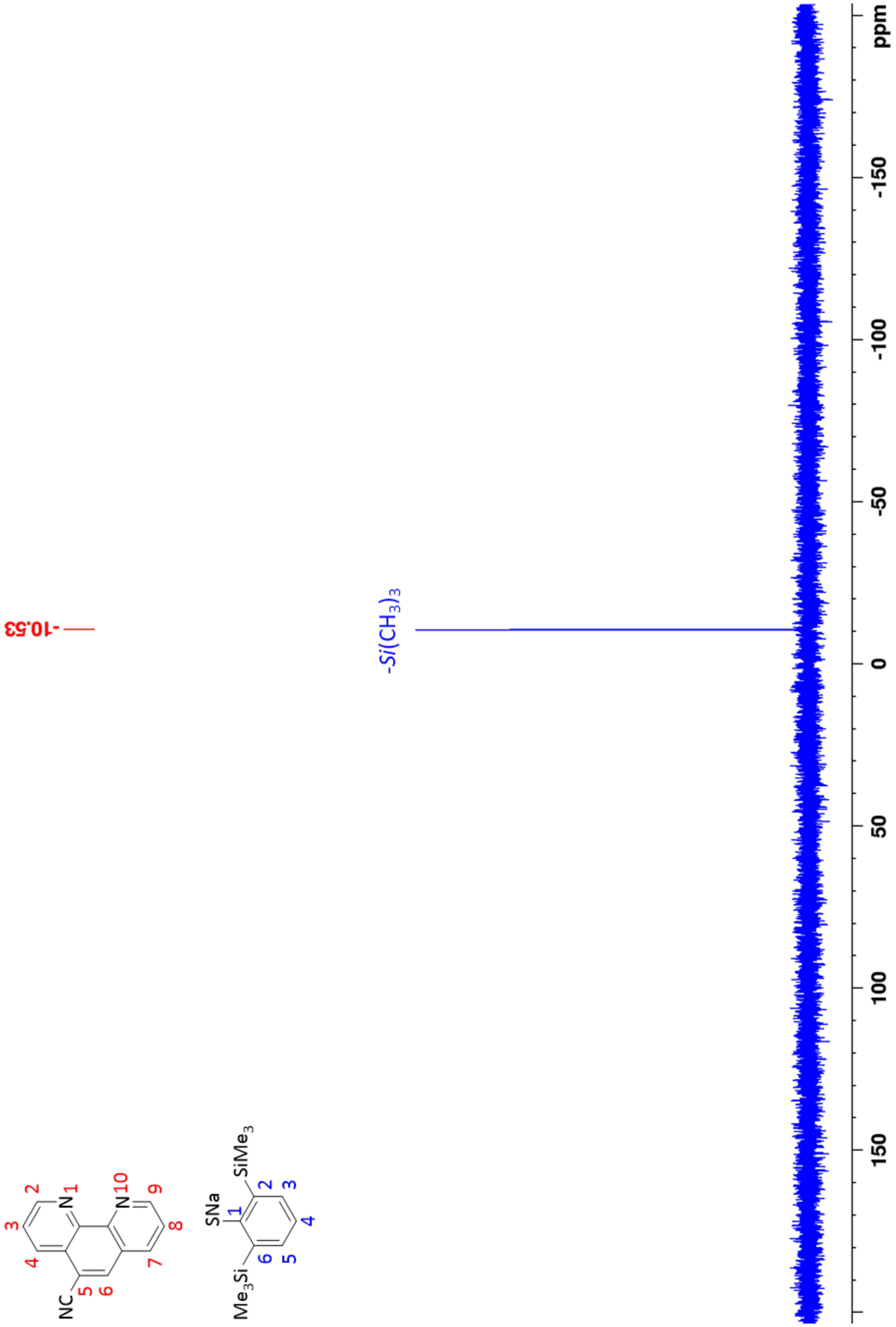

Supplementary Fig. 14. ^{29}Si NMR (THF-d_8, 119 MHz, 298 K) of **Na1D**.

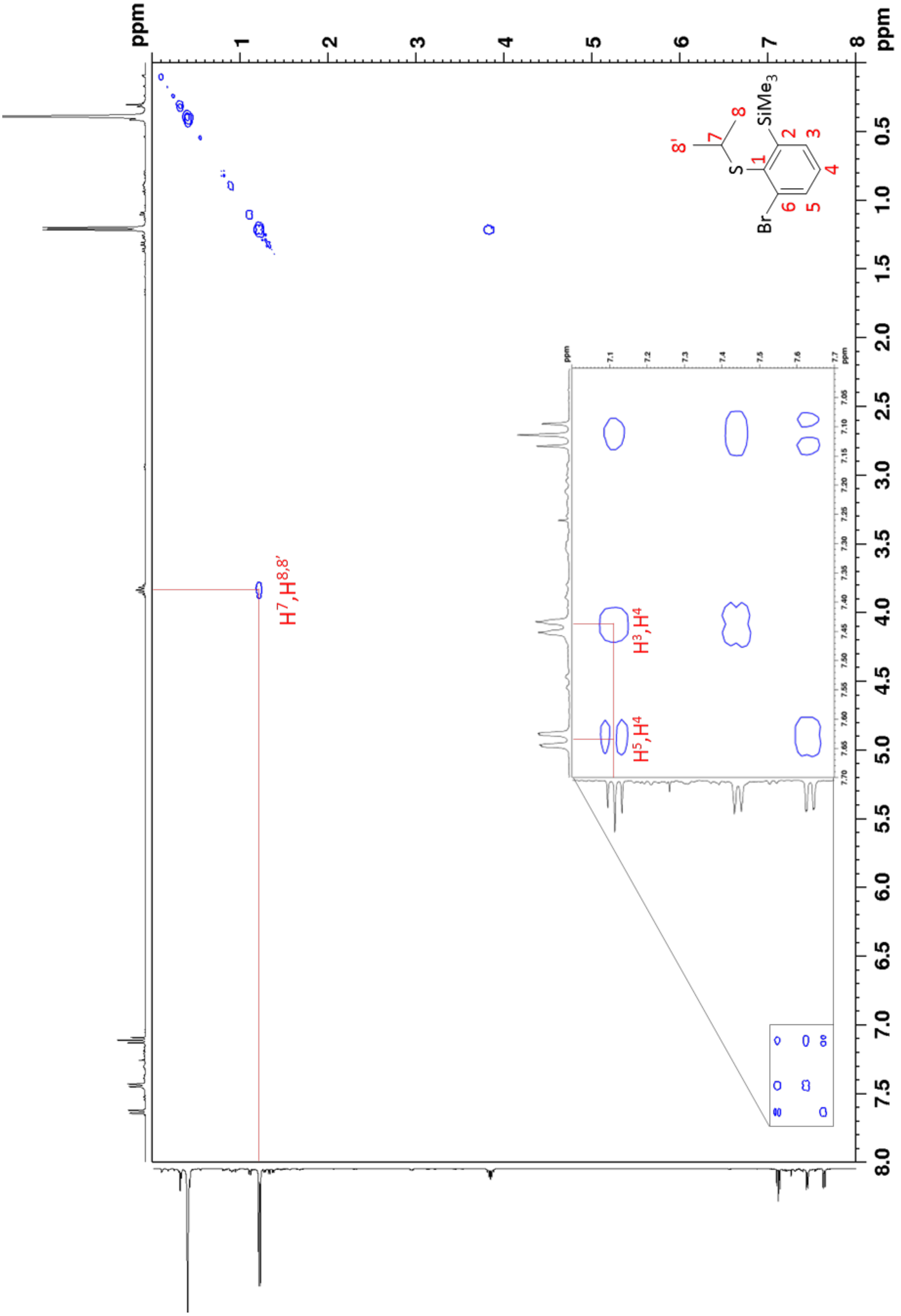

Supplementary Fig. 15. ^{1}H-^{1}H COSY NMR ($CDCl_3$, 400 MHz, 297 K) of **4**.

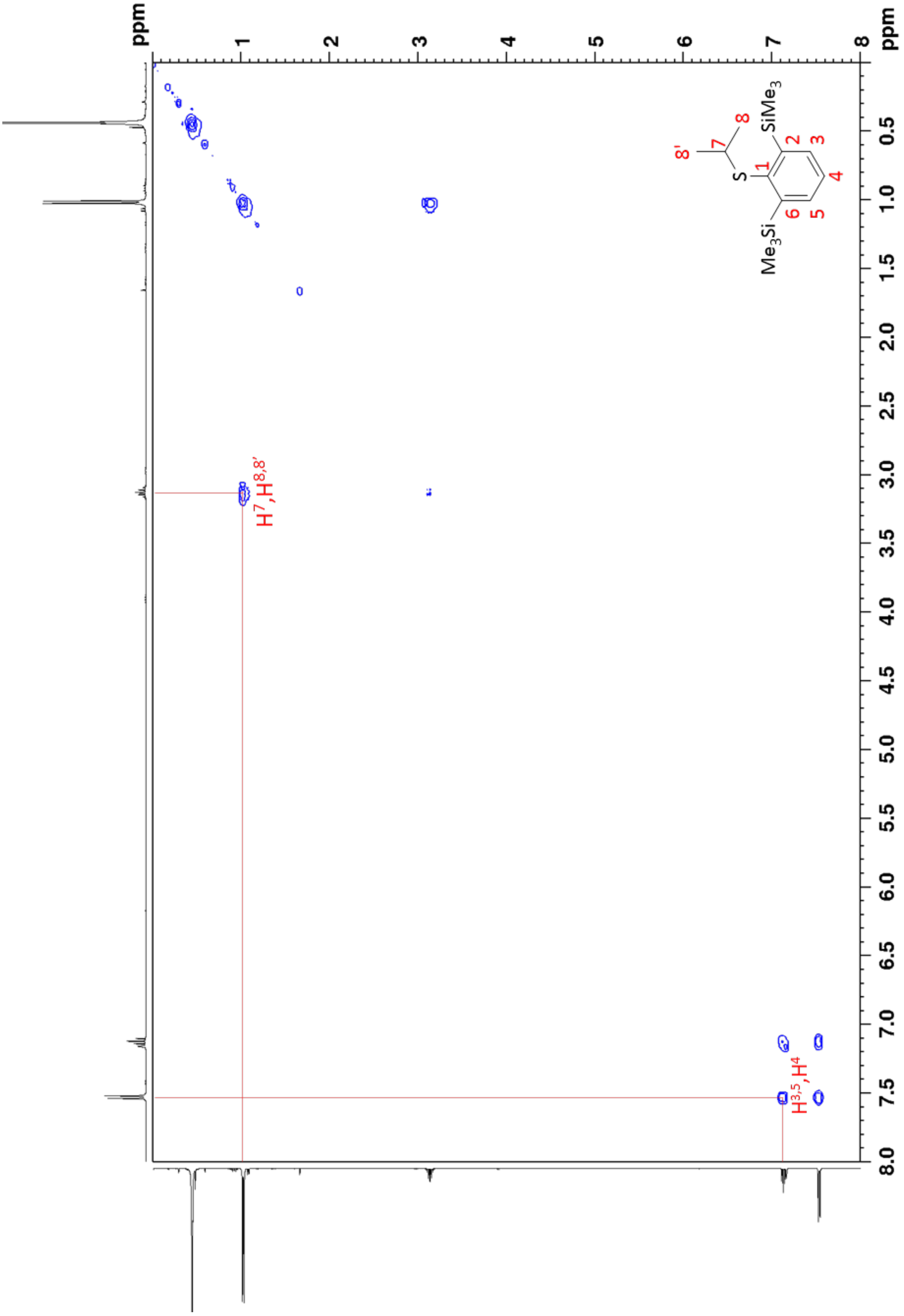

Supplementary Fig. 16. ^{1}H-^{1}H COSY NMR (C_6D_6, 400 MHz, 298 K) of **5**.

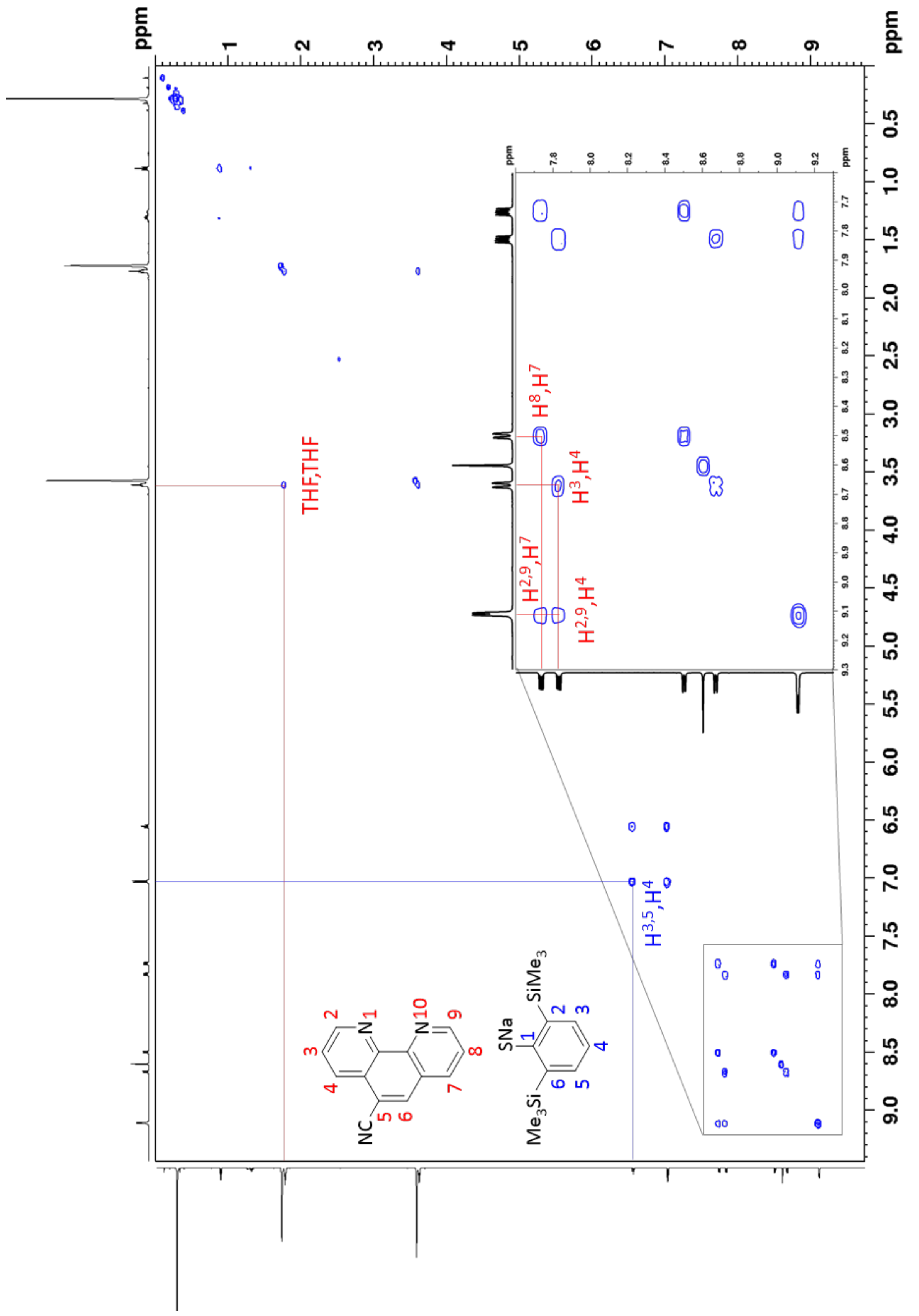

Supplementary Fig. 17. ^{1}H-^{1}H COSY NMR (THF-d$_8$, 600MHz, 298 K) of **Na1D**.

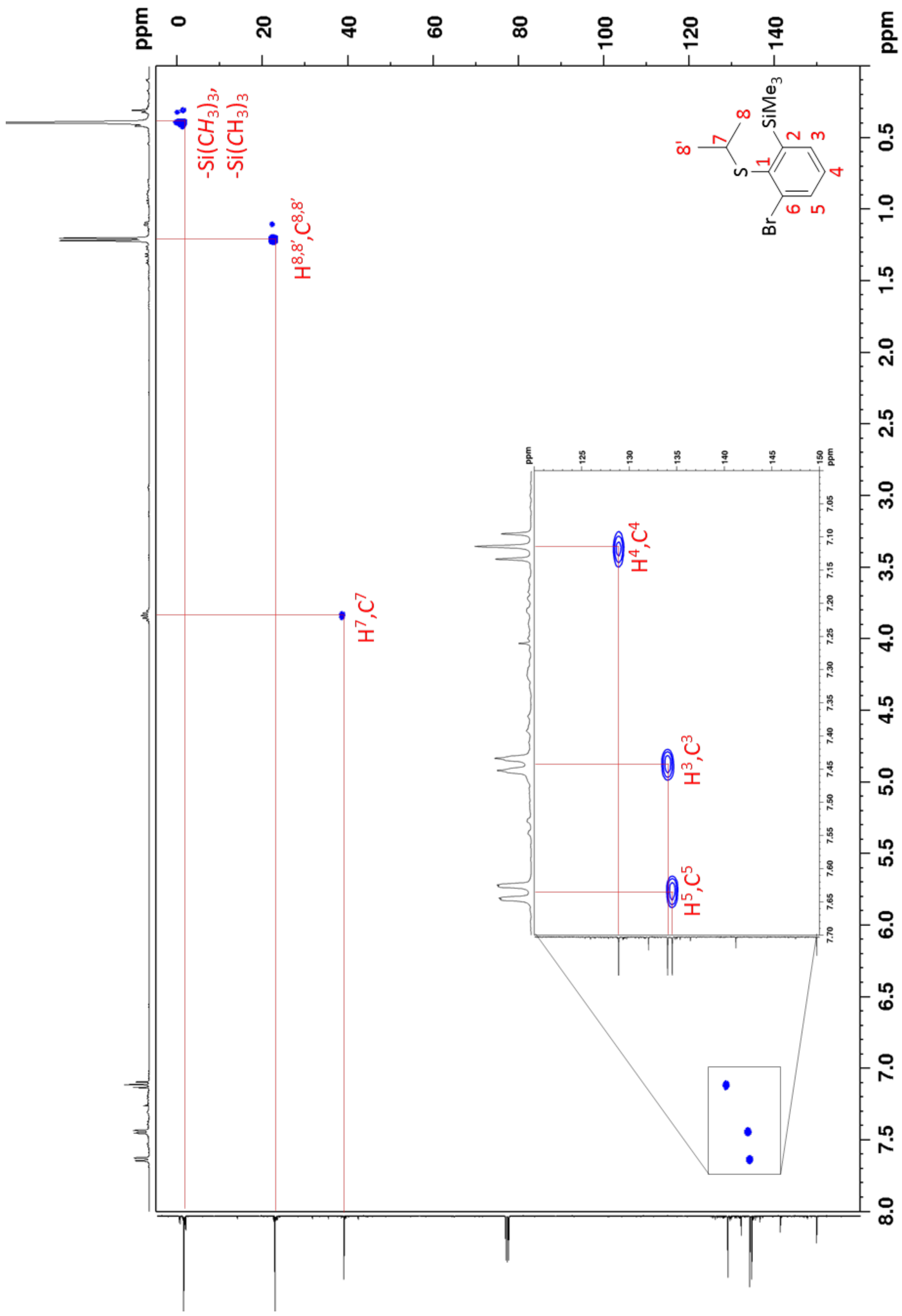

Supplementary Fig. 18. ^{1}H-^{13}C HSQC NMR (CDCl$_3$, 400 MHz, 297 K) of **4**.

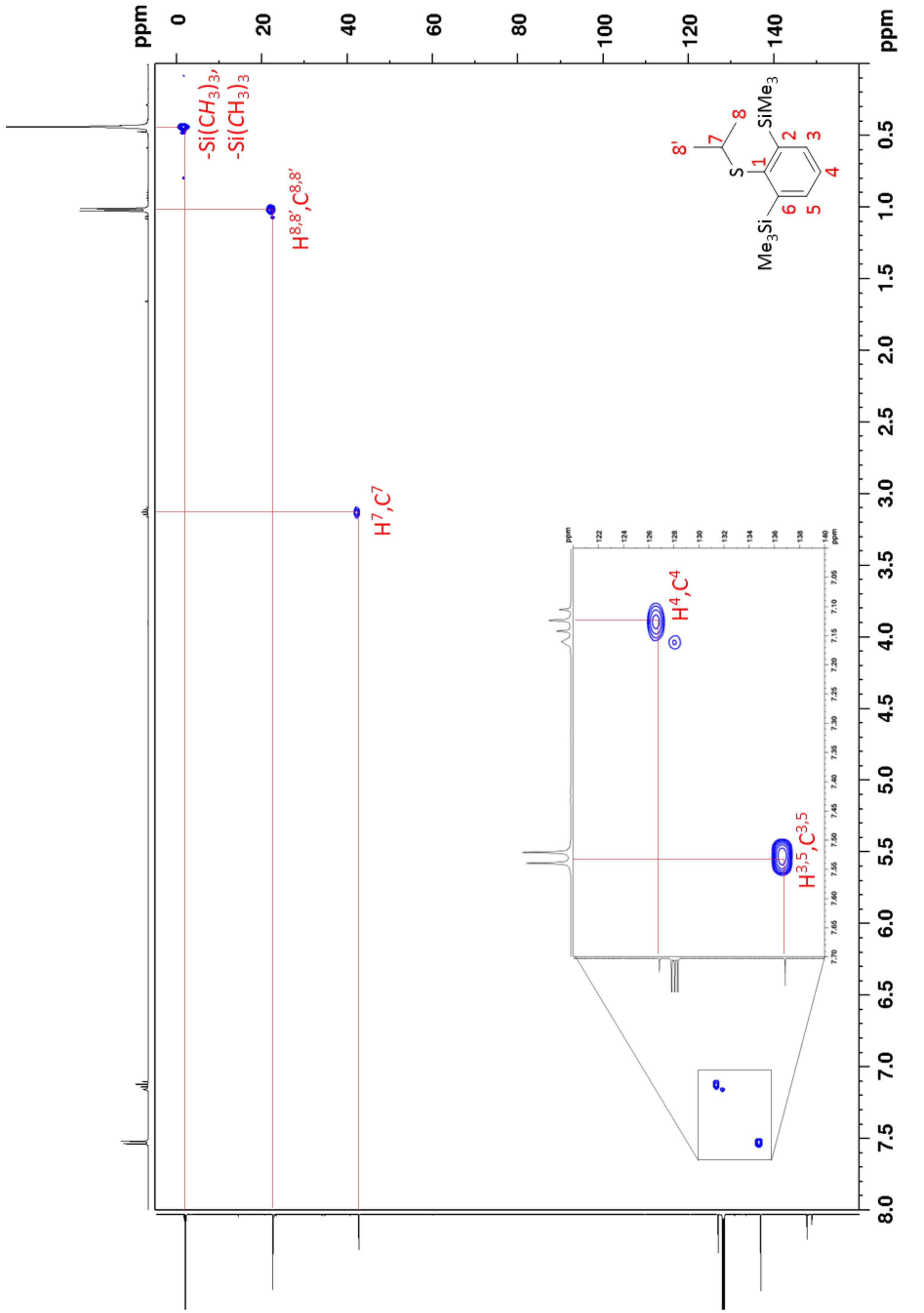

Supplementary Fig. 19. ^{1}H-^{13}C HSQC NMR (C_6D_6, 400 MHz, 298 K) of **5**.

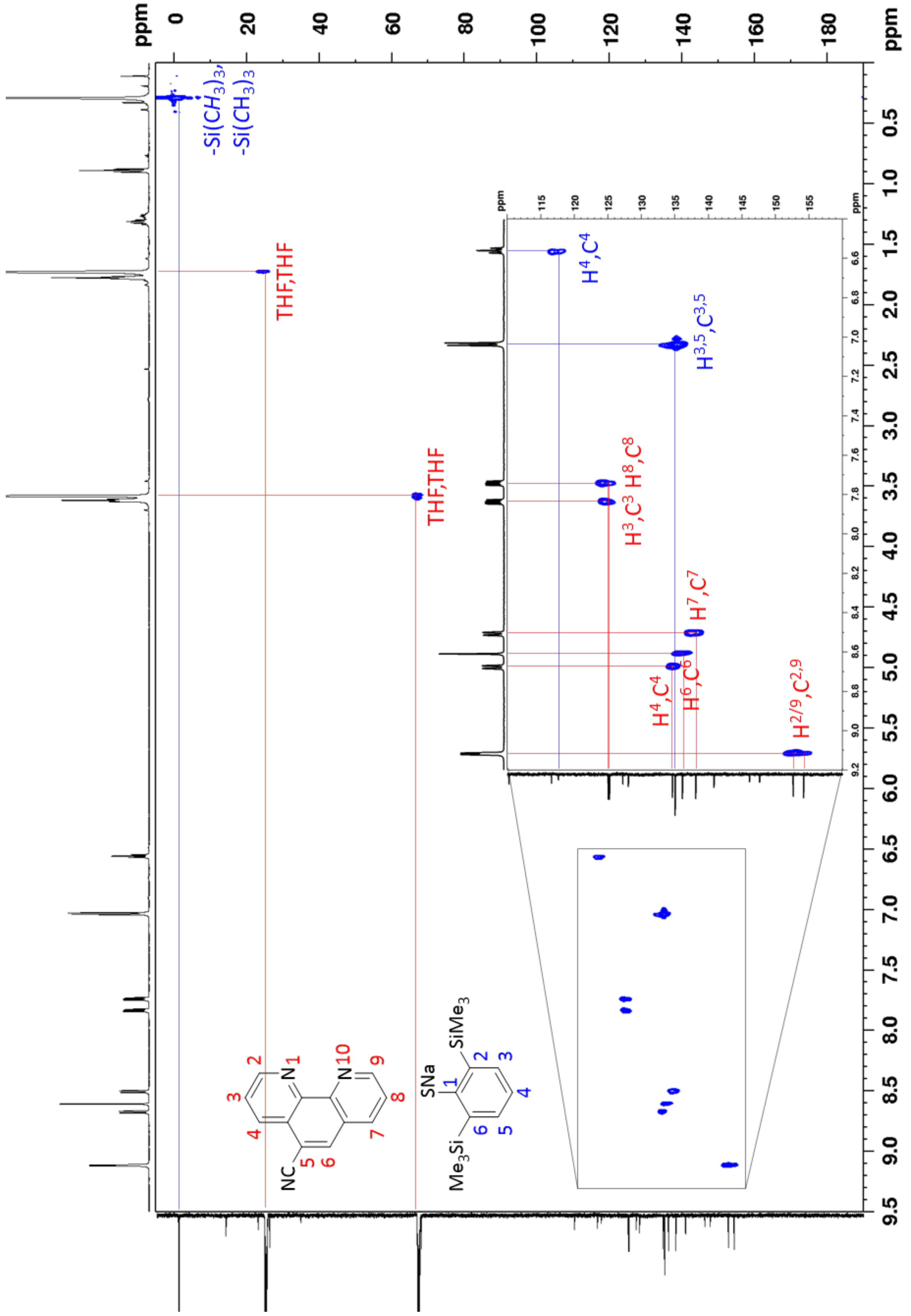

Supplementary Fig. 20. ^{1}H-^{13}C HSQC NMR (THF-d_8, 600 MHz, 298 K) of **Na1D**.

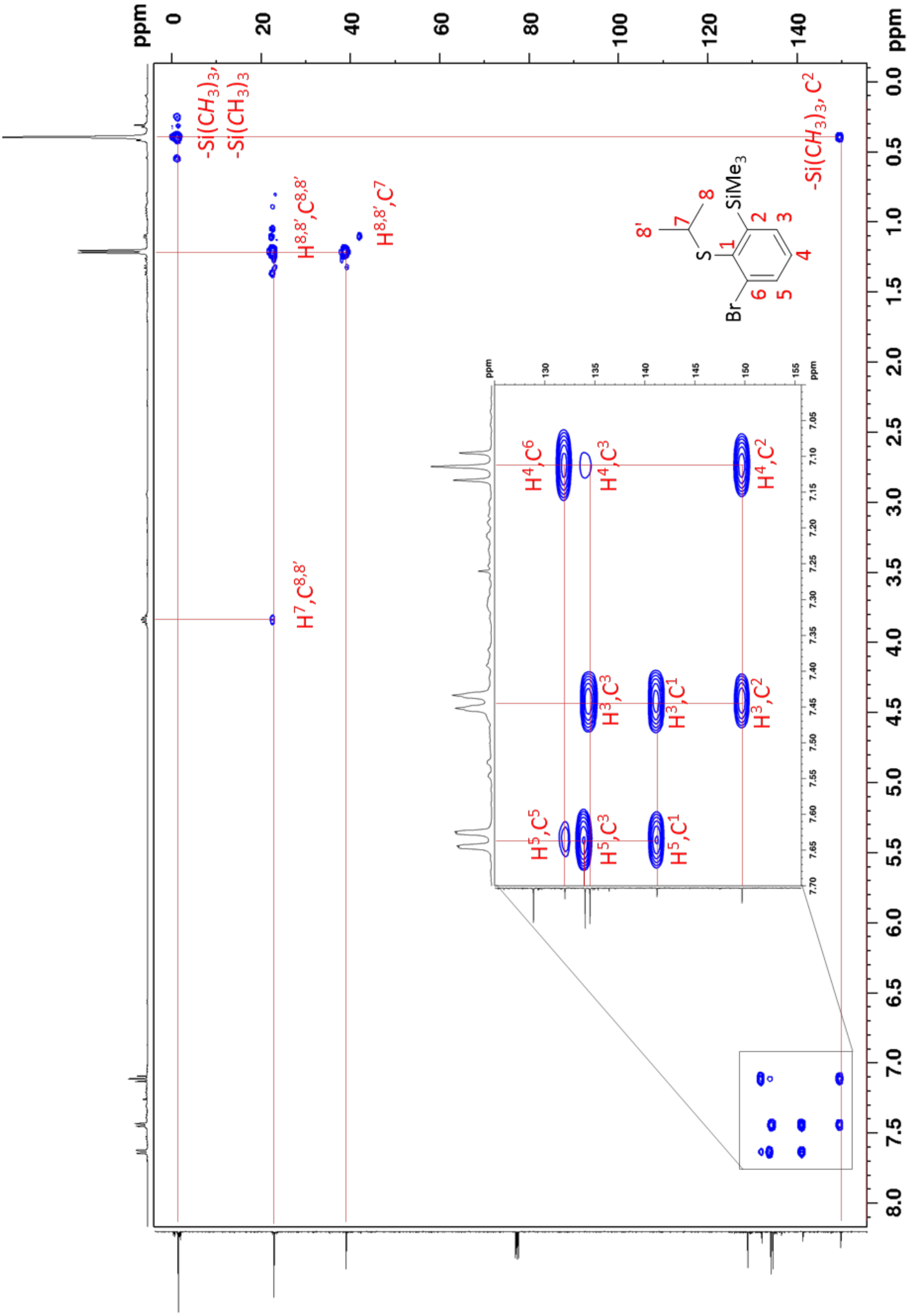

Supplementary Fig. 21. ^{1}H-^{13}C HMBC NMR (CDCl$_3$, 400 MHz, 297 K) of **4**.

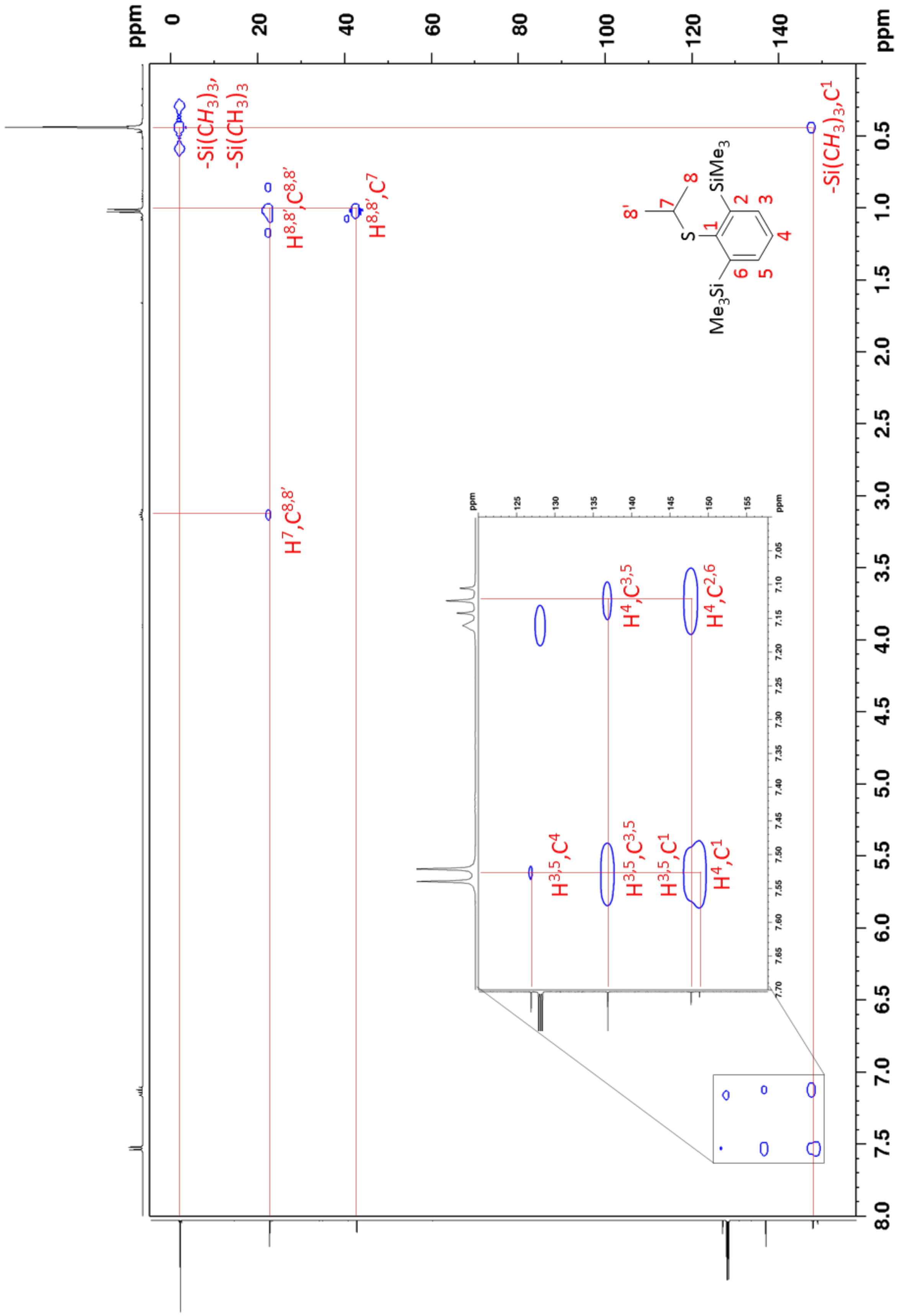

Supplementary Fig. 22. ^{1}H-^{13}C HMBC NMR (C_6D_6, 400 MHz, 298 K) of **5**.

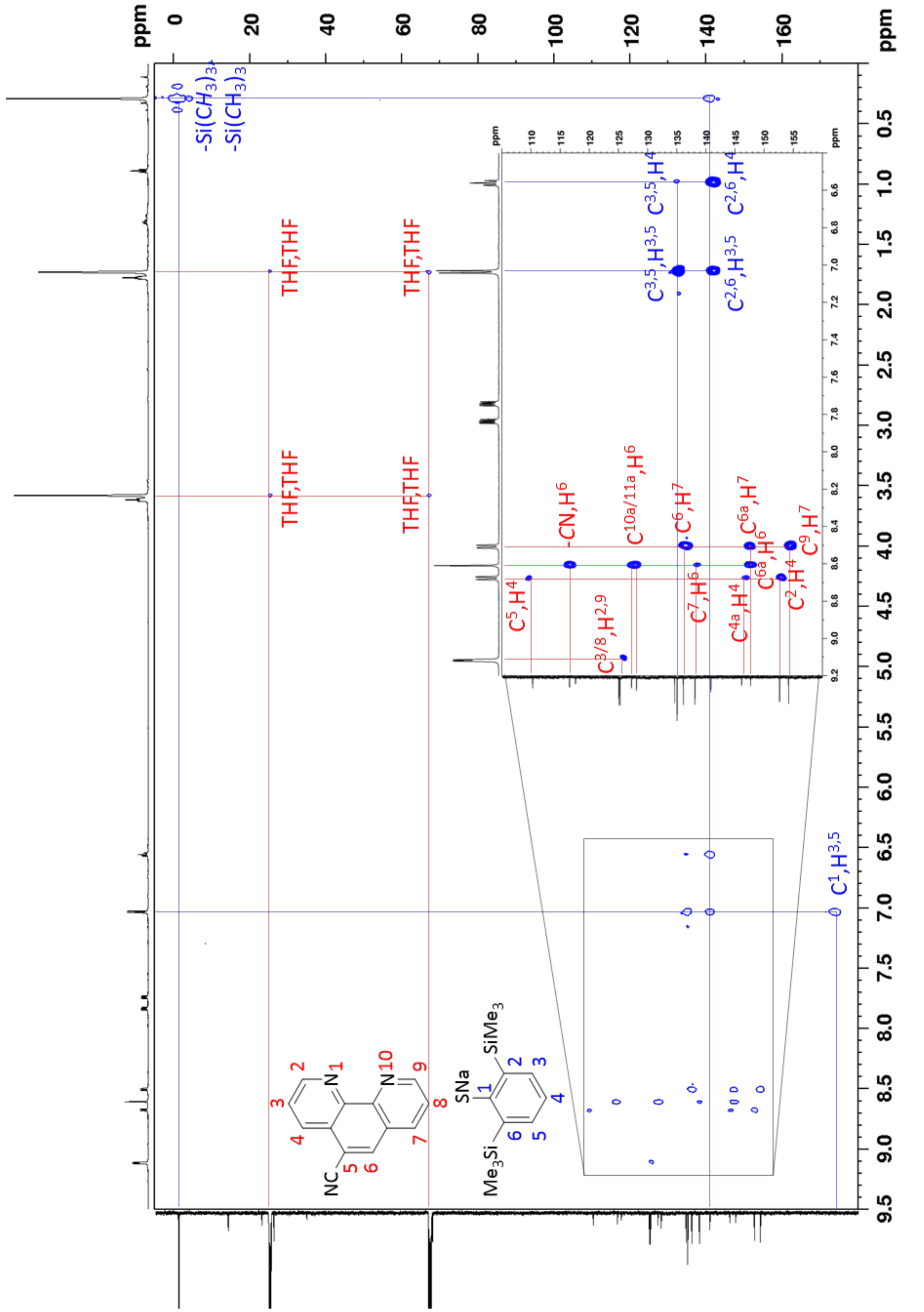

Supplementary Fig. 23. ^{1}H-^{13}C HMBC NMR (THF-d$_8$, 600 MHz, 298 K) of **Na1D**.

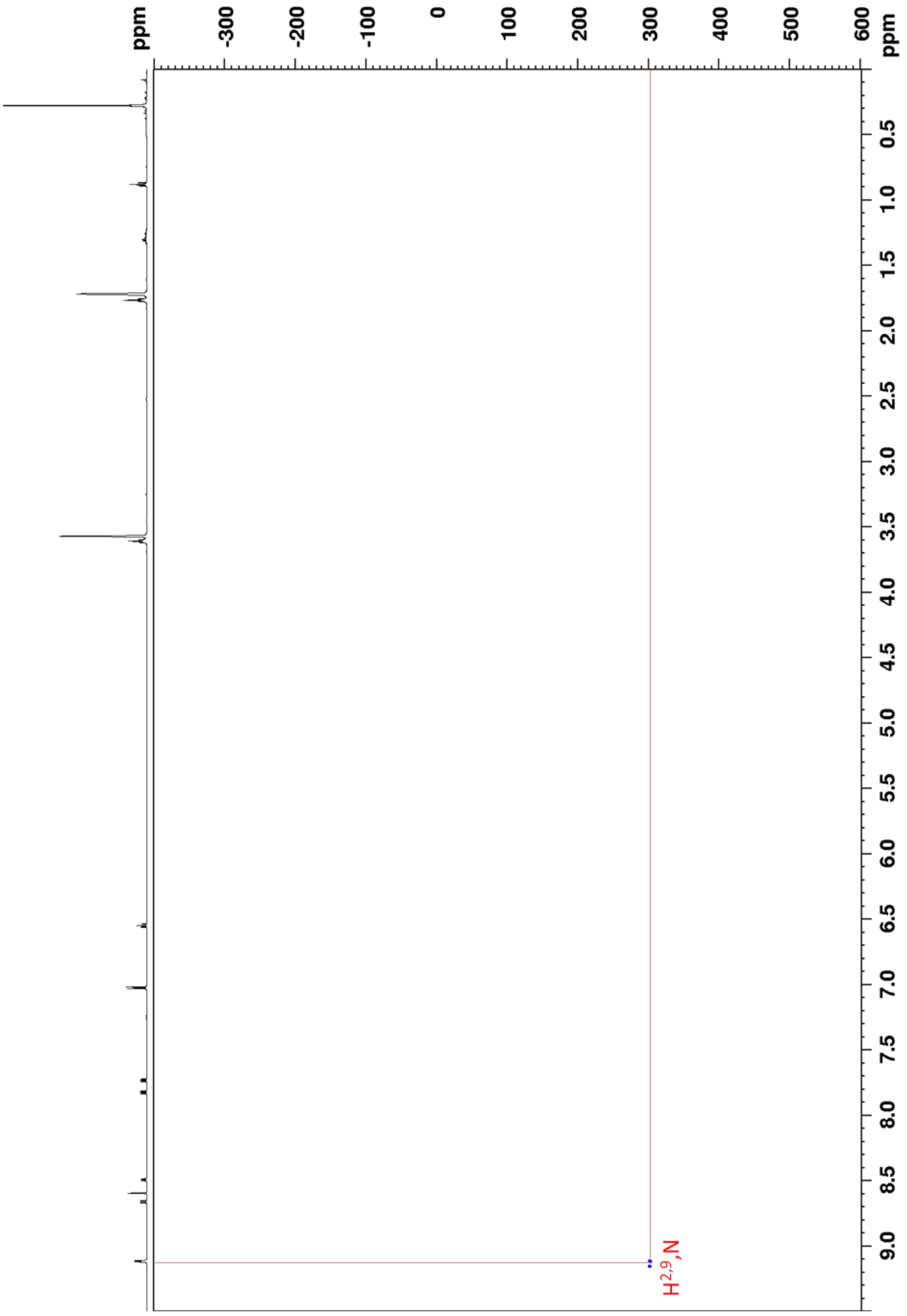

Supplementary Fig. 24. ^{1}H-^{15}N HMBC NMR (THF-d_8, 60 and 600 MHz, 298 K) of **Na1D.**

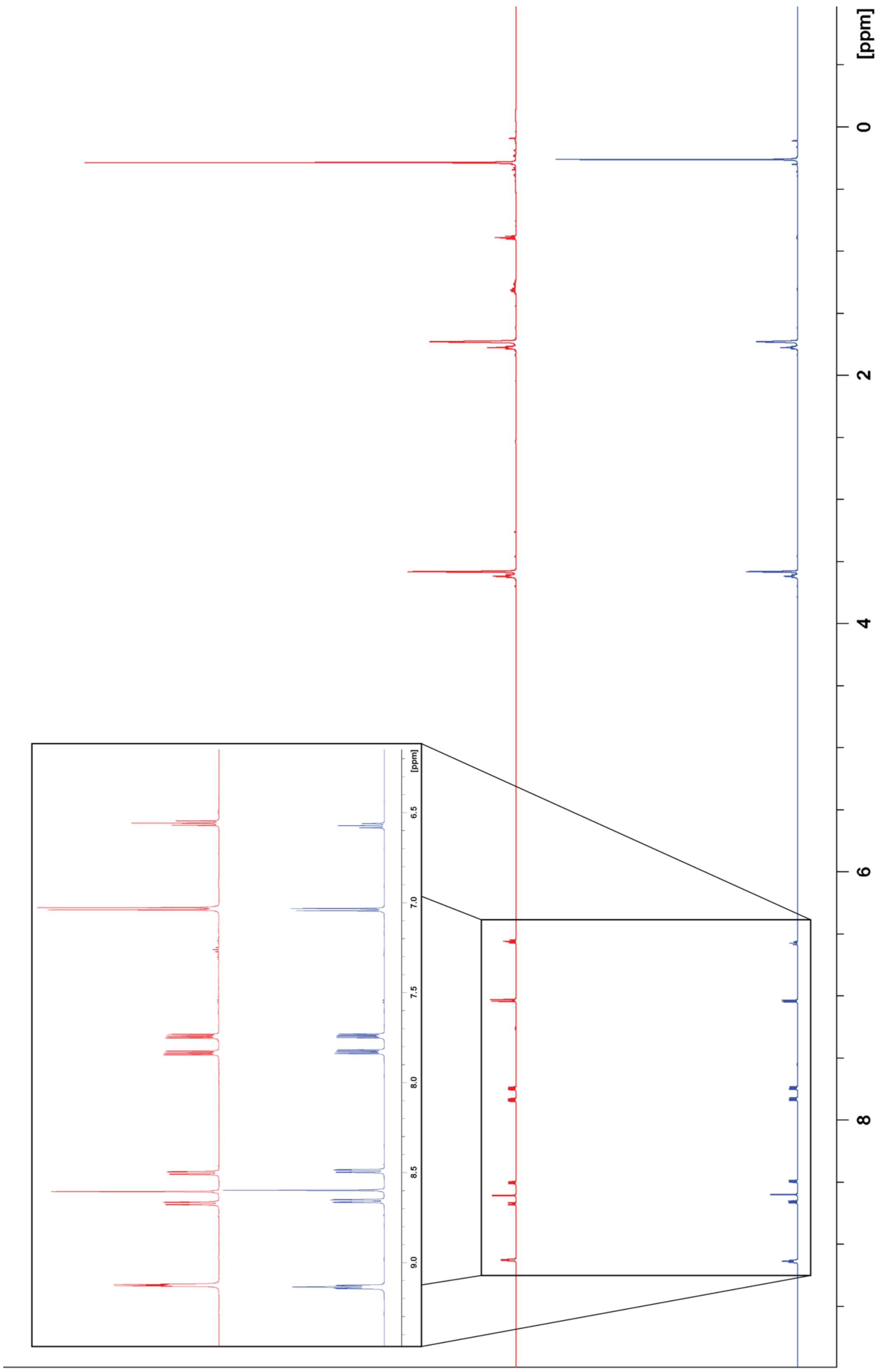

Supplementary Fig. 25. Comparison of ^{1}H NMR spectra of **Na1D** and **Na2D** (THF-d_8, 60 and 600 MHz, 298 K).

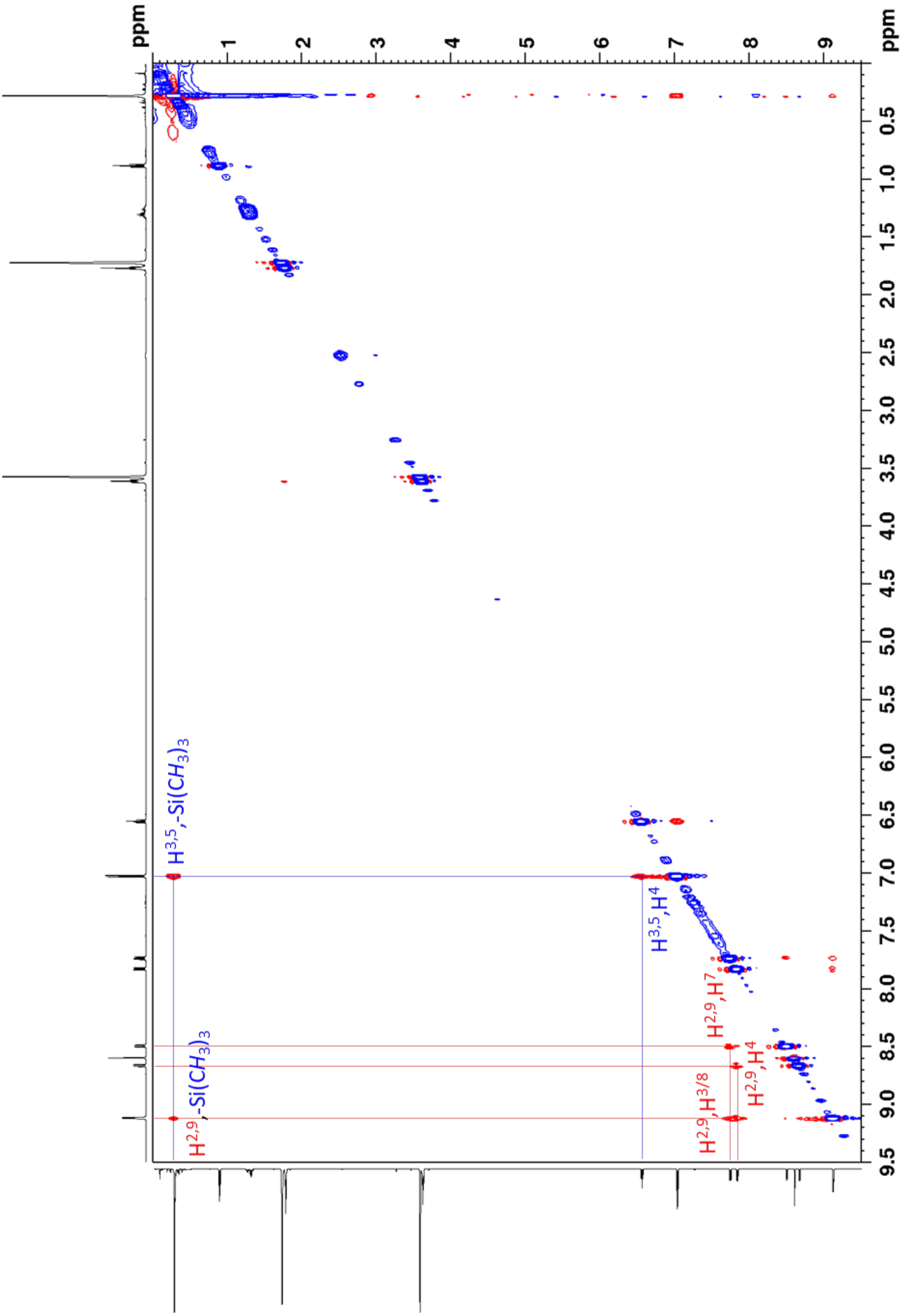

Supplementary Fig. 26. ^{1}H-^{1}H NOESY NMR (THF-d_8, 600 MHz, 298 K) of **Na1D**.

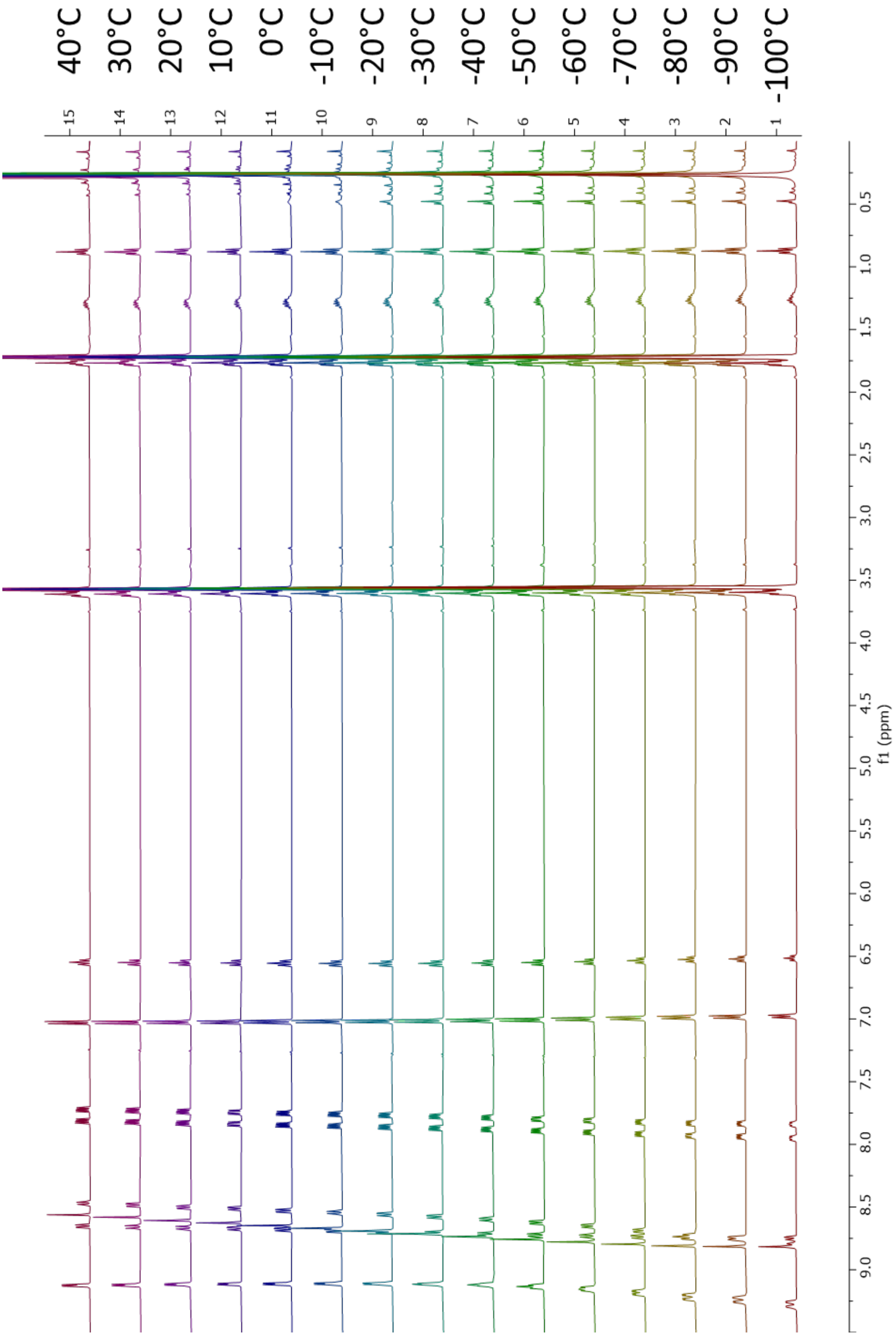

Supplementary Fig. 27. ^{1}H NMR data (THF-d_8, 400 MHz) of **Na1D** at -100 – 40°C.

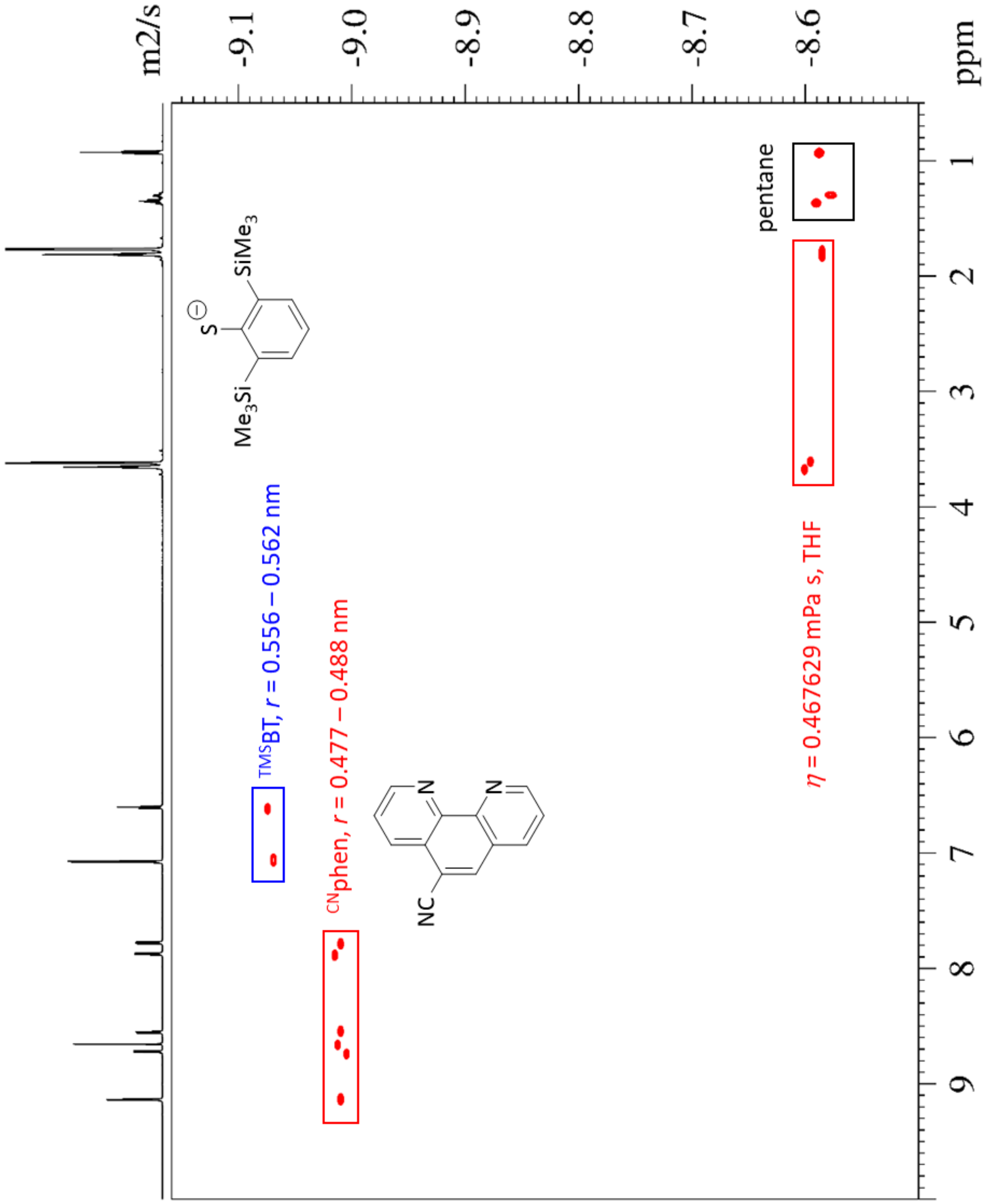

Supplementary Fig. 28. ^{1}H-DOSY NMR (THF-d_8, 700 MHz, 298 K) of **Na1D**.

DOSY NMR analysis

Supporting Table 1. Details about diffusion analysis of **Na1D** in THF-d_8 showing multiple species in solution.

Fitted function:	f (x) = Io * exp (-D * x^2 * gamma^2 * littleDelta^2 (bigDelta-littleDelta/3)* 10^4
used gamma:	26752 rad/(s*Gauss)
used little delta:	0.0016000 s
used big delta:	0.097270 s
used gradient strength:	variable
Random error estimation of data:	RMS per spectrum (or trace/plane)
Systematic error estimation of data:	worst case per peak scenario
Fit parameter Error estimation method:	from fit using arbitrary y uncertainties
Confidence level:	95%
Used peaks:	
Used integrals:	peak intensities
Used Gradient strength:	all values (including replicates) used

Supporting Table 2. Diffusion coefficients (D) for peaks in **Na1D** in THF-d_8 and radii (r) and diameter (d) calculated according to spherical Stokes-Einstein equation. Red: ^{CN}phen ligand, blue: ^{TMS}BT ligand, black: solvent

Peak name	F2 [ppm]	*D* [m^2/s]	error	errorScale	*r* [m]	*r* [nm]	*d*[nm]
1	9.139839961	9.426E-10	0.0000000000	1.978970602	4.95E-10	0.495	0.991
2	9.133842427	9.4918E-10	7.16505E-12	1.978970602	4.92E-10	0.492	0.984
3	8.7257478	9.88417E-10	7.55211E-12	1.978970602	4.72E-10	0.472	0.945
4	8.715756512	9.50913E-10	1.13971E-11	1.978970602	4.91E-10	0.491	0.982
5	8.655809988	9.24081E-10	7.74323E-12	1.978970602	5.05E-10	0.505	1.011
6	8.558397337	9.37795E-10	7.55272E-12	1.978970602	4.98E-10	0.498	0.996
7	8.546907716	9.21767E-10	9.76699E-12	1.978970602	5.07E-10	0.507	1.013
8	7.881308726	9.40019E-10	7.40543E-12	1.978970602	4.97E-10	0.497	0.994
9	7.874811748	9.43695E-10	1.14156E-11	1.978970602	4.95E-10	0.495	0.990
10	7.869314258	9.50471E-10	1.01675E-11	1.978970602	4.91E-10	0.491	0.983

11	7.86281728	9.53513E-10	9.64107E-12	1.978970602	4.90E-10	0.490	0.979
12	7.784353498	9.37575E-10	6.12996E-12	1.978970602	4.98E-10	0.498	0.996
13	7.77785652	9.53311E-10	5.88562E-12	1.978970602	4.90E-10	0.490	0.980
14	7.772859075	9.4336E-10	6.22074E-12	1.978970602	4.95E-10	0.495	0.990
15	7.766362097	9.49198E-10	8.54747E-12	1.978970602	4.92E-10	0.492	0.984
16	7.080104601	8.16863E-10	5.39588E-12	1.978970602	5.72E-10	0.572	1.143
17	7.069613869	8.17175E-10	7.72812E-12	1.978970602	5.71E-10	0.571	1.143
18	6.601462864	8.0036E-10	5.68158E-12	1.978970602	5.83E-10	0.583	1.167
19	6.591467375	8.18132E-10	7.29899E-12	1.978970602	5.71E-10	0.571	1.142
20	6.611958398	7.85849E-10	7.54807E-12	1.978970602	5.94E-10	0.594	1.188
21	3.653753334	2.53652E-09	2.12424E-11	1.978970602	1.84E-10	0.184	0.368
22	3.617271088	2.42208E-09	2.86274E-11	1.978970602	1.93E-10	0.193	0.386
23	1.811609414	2.60093E-09	1.38784E-11	1.978970602	1.80E-10	0.180	0.359
24	1.765151488	2.62275E-09	3.29066E-11	1.978970602	1.78E-10	0.178	0.356
25	1.346860873	2.62738E-09	1.29698E-11	1.978970602	1.78E-10	0.178	0.355
26	1.302400724	2.65591E-09	2.45836E-11	1.978970602	1.76E-10	0.176	0.352
27	0.925968585	2.5799E-09	2.17954E-11	1.978970602	1.81E-10	0.181	0.362
28	0.318693525	8.10133E-10	6.75515E-12	1.978970602	5.76E-10	0.576	1.153

Supporting Fig. 29. Correlation of every peak's intensity decay (black points) with the fitted function (blue dotted line) in the ^{1}H DOSY NMR of **Na1D** in THF-d_8.

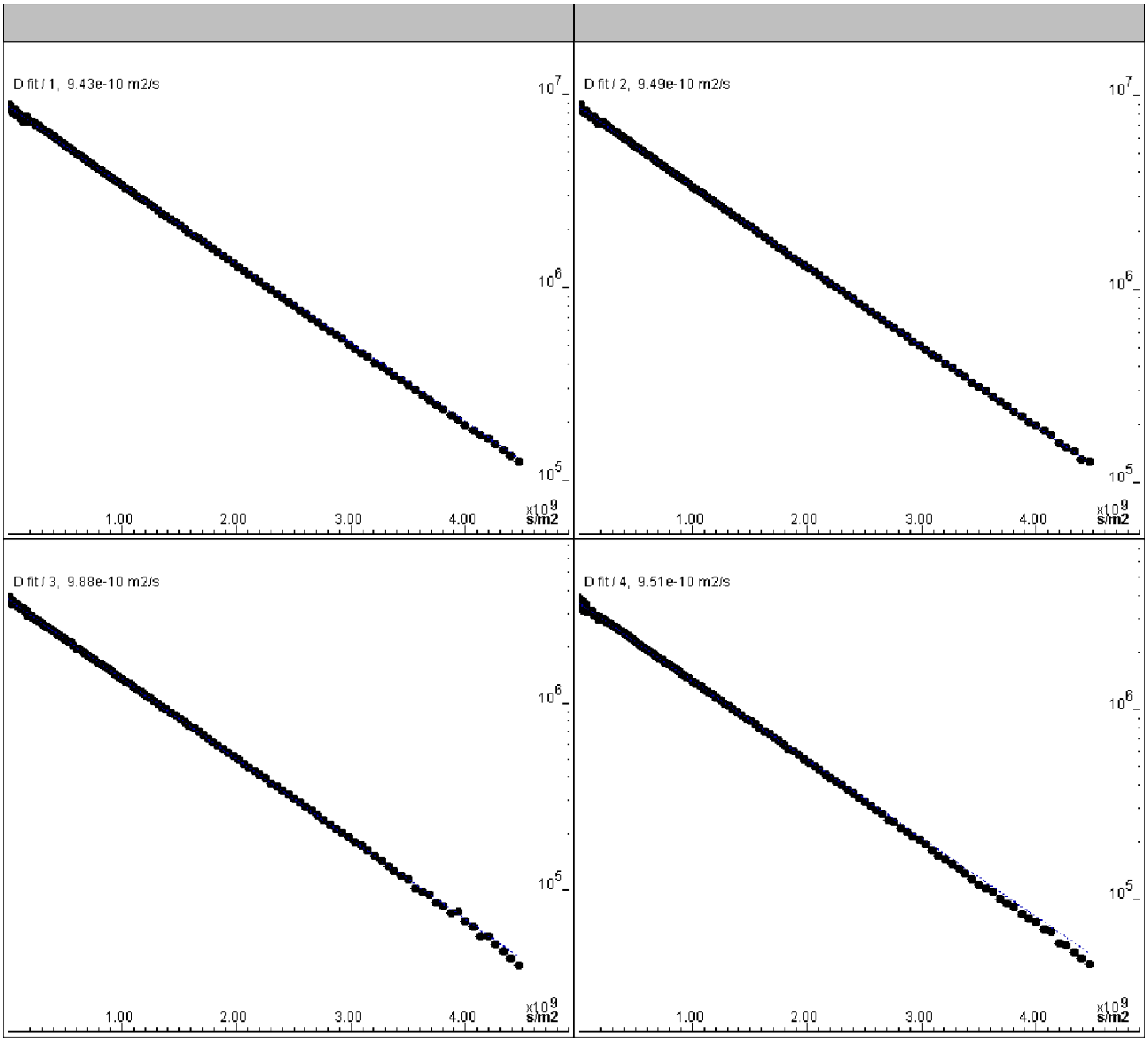

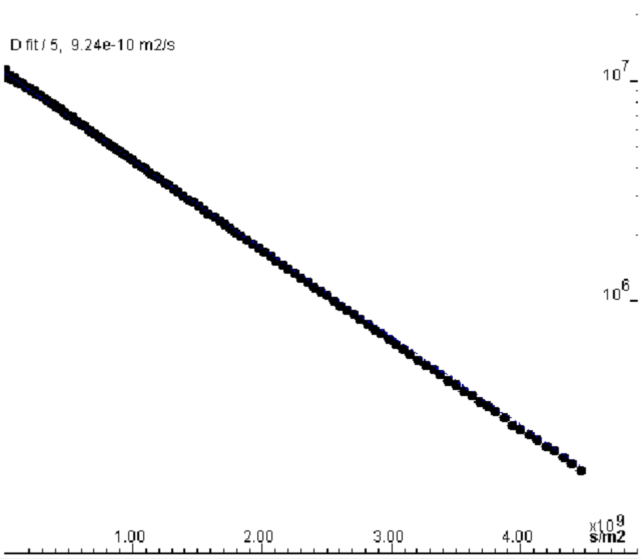
D fit / 5, 9.24e-10 m2/s
10^7
10^6
1.00
2.00
3.00
4.00
x10^9
s/m2

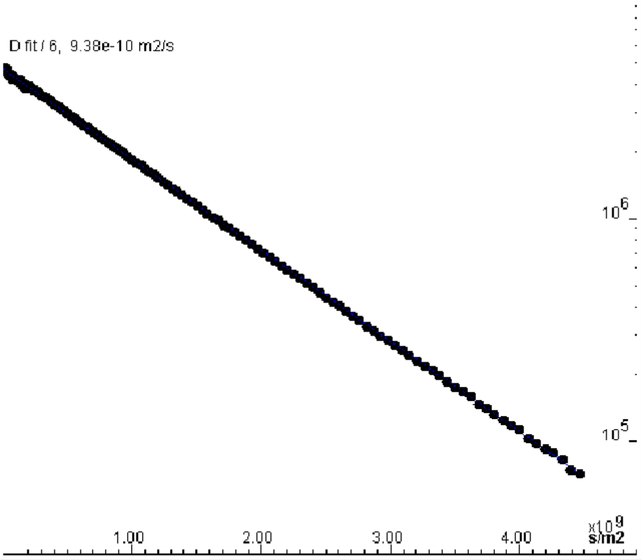
D fit / 6, 9.38e-10 m2/s
10^6
10^5
1.00
2.00
3.00
4.00
x10^9
s/m2

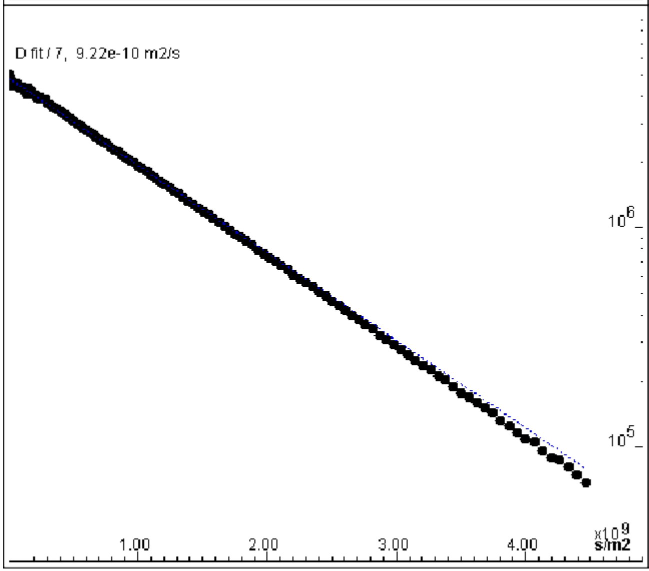
D fit / 7, 9.22e-10 m2/s
10^6
10^5
1.00
2.00
3.00
4.00
x10^9
s/m2

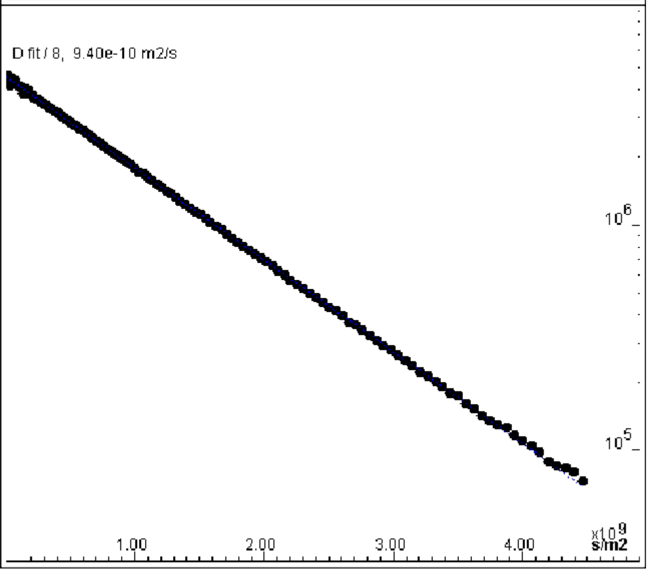
D fit / 8, 9.40e-10 m2/s
10^6
10^5
1.00
2.00
3.00
4.00
x10^9
s/m2

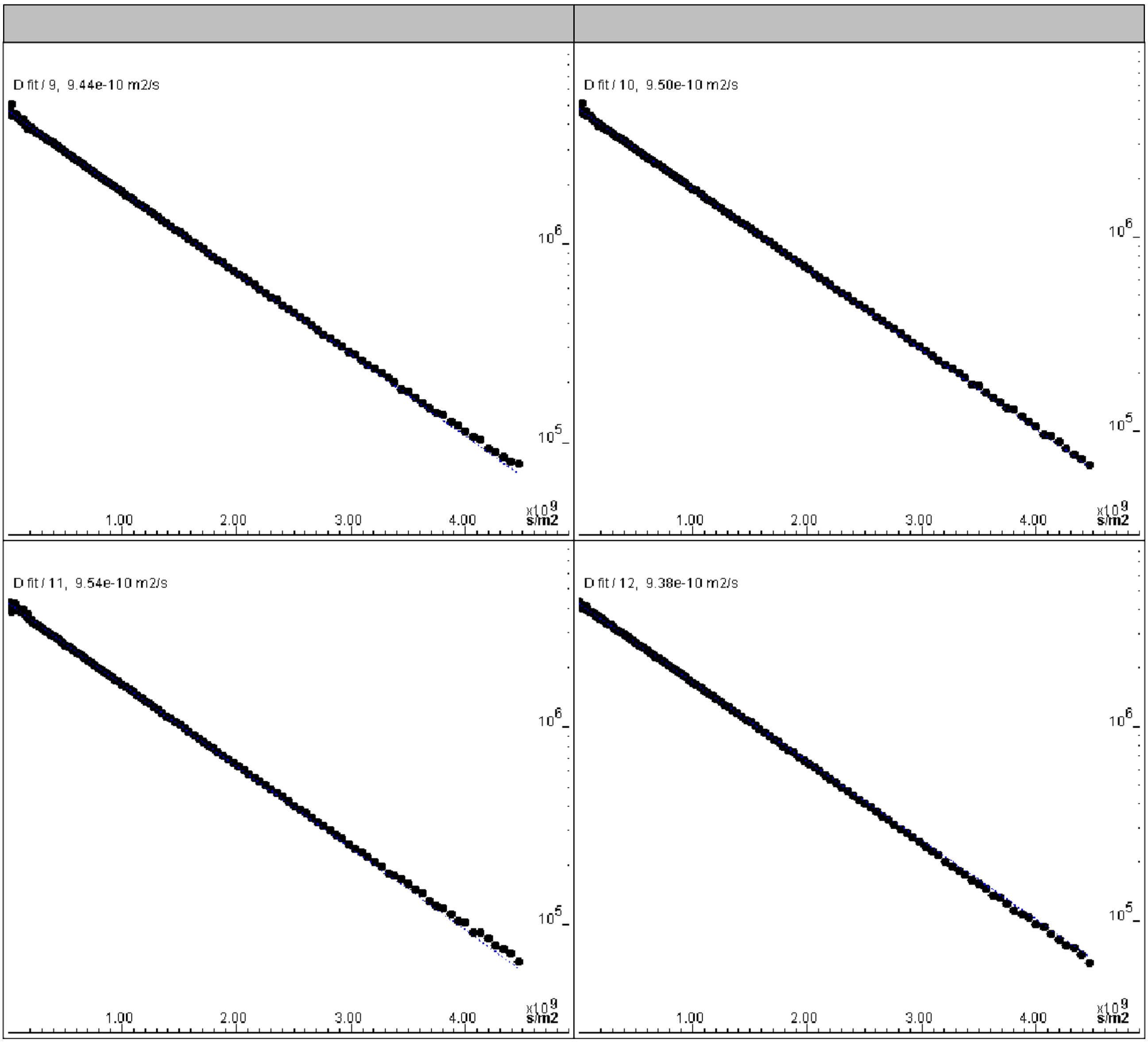
D fit / 9, 9.44e-10 m2/s
D fit / 10, 9.50e-10 m2/s
D fit / 11, 9.54e-10 m2/s
D fit / 12, 9.38e-10 m2/s
10^6
10^5
1.00
2.00
3.00
4.00
x10^9
s/m2

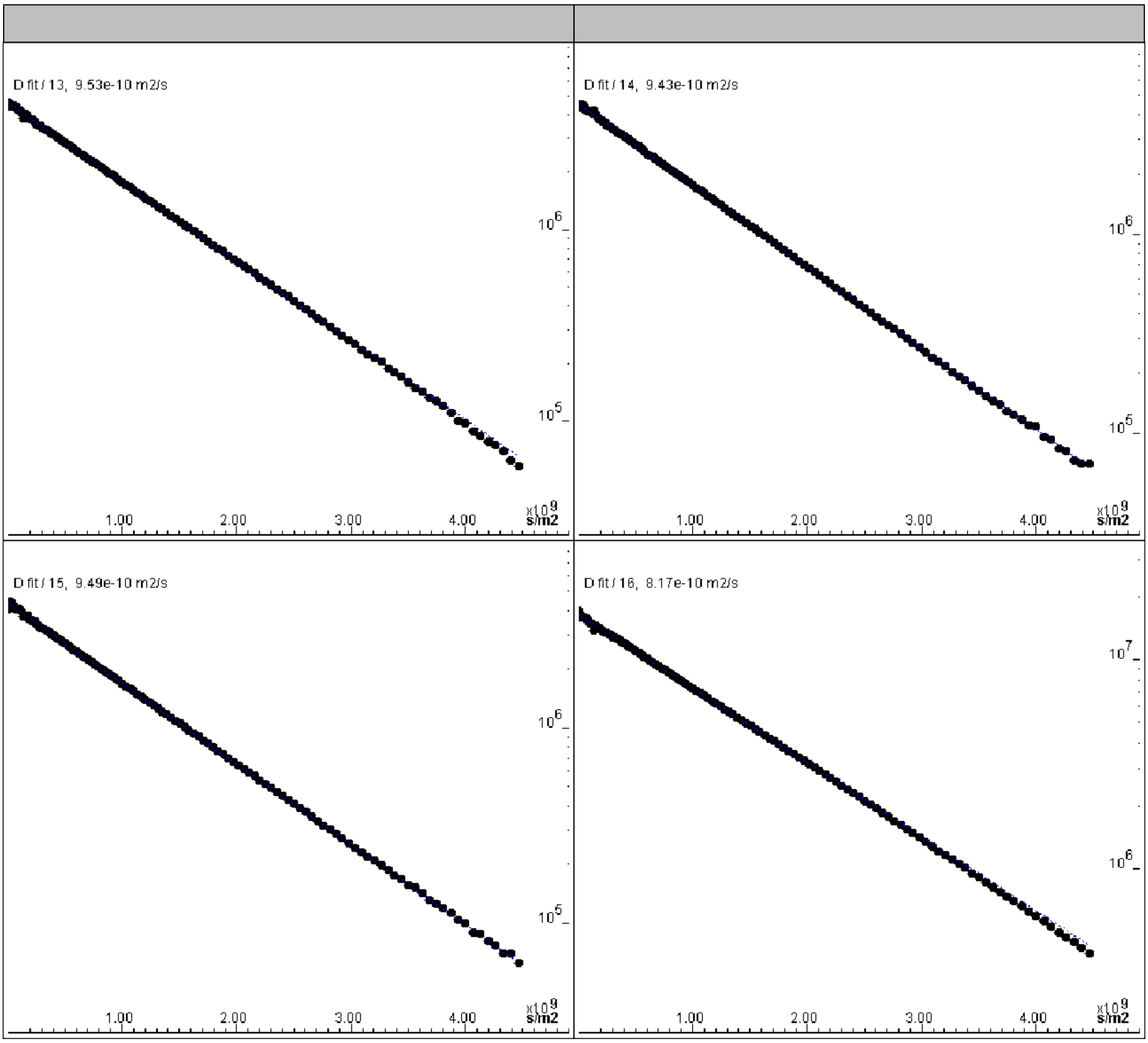
D fit / 13, 9.53e-10 m2/s
10^6
10^5
1.00
2.00
3.00
4.00
x10^9
s/m2
D fit / 14, 9.43e-10 m2/s
10^6
10^5
1.00
2.00
3.00
4.00
x10^9
s/m2
D fit / 15, 9.49e-10 m2/s
10^6
10^5
1.00
2.00
3.00
4.00
x10^9
s/m2
D fit / 16, 8.17e-10 m2/s
10^7
10^6
1.00
2.00
3.00
4.00
x10^9
s/m2

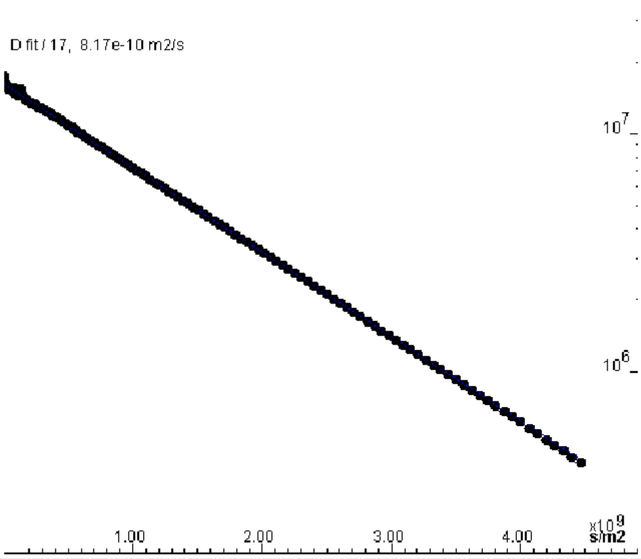
D fit / 17, 8.17e-10 m2/s
10^7
10^6
1.00
2.00
3.00
4.00
x10^9
s/m2

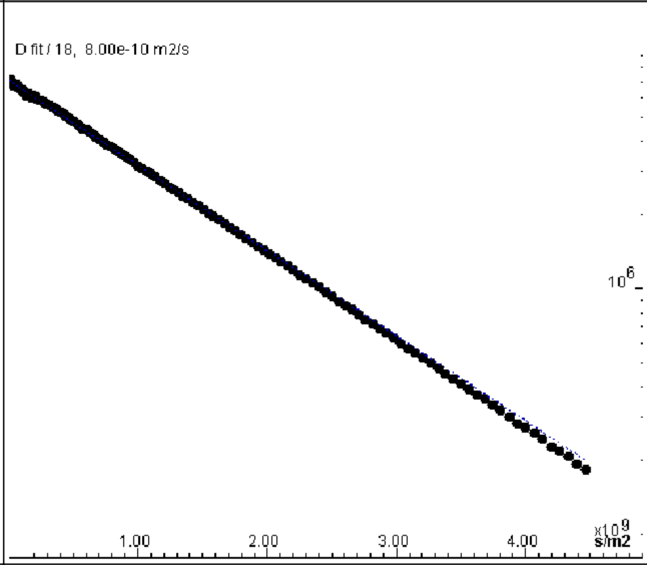
D fit / 18, 8.00e-10 m2/s
10^6
1.00
2.00
3.00
4.00
x10^9
s/m2

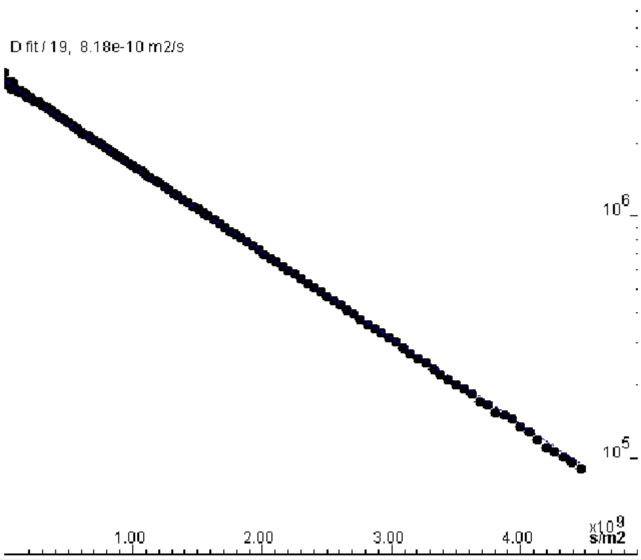
D fit / 19, 8.18e-10 m2/s
10^6
10^5
1.00
2.00
3.00
4.00
x10^9
s/m2

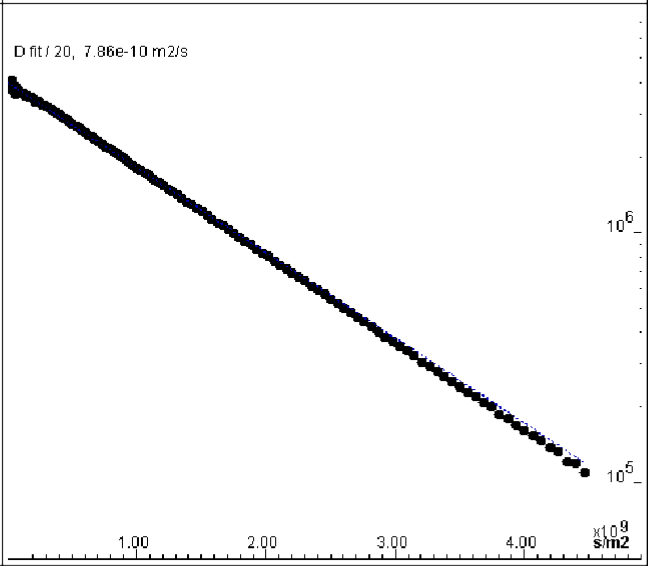
D fit / 20, 7.86e-10 m2/s
10^6
10^5
1.00
2.00
3.00
4.00
x10^9
s/m2

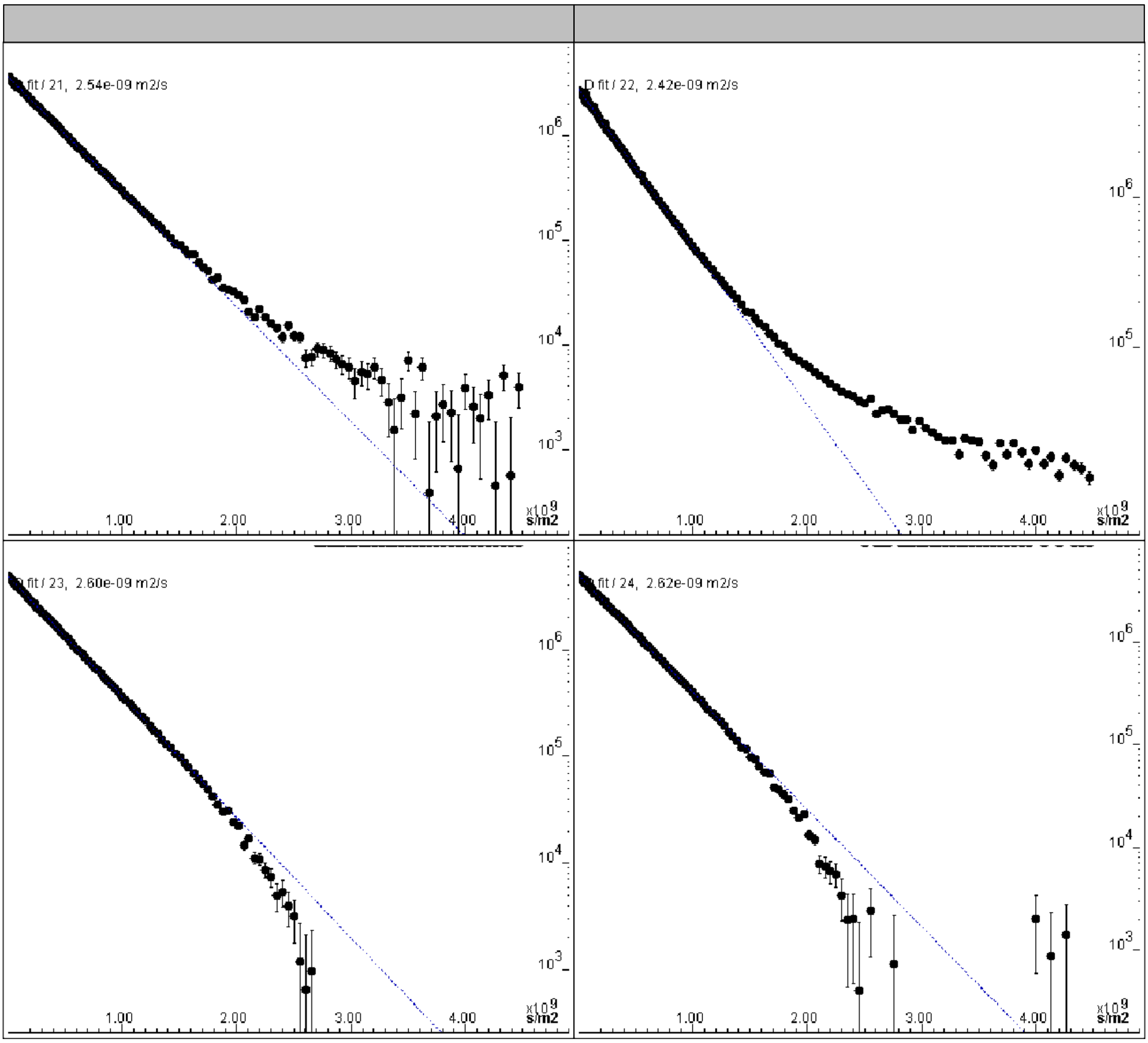
fit / 21, 2.54e-09 m2/s
D fit / 22, 2.42e-09 m2/s
fit / 23, 2.60e-09 m2/s
fit / 24, 2.62e-09 m2/s
1.00
2.00
3.00
4.00
x10^9
s/m2
10^3
10^4
10^5
10^6

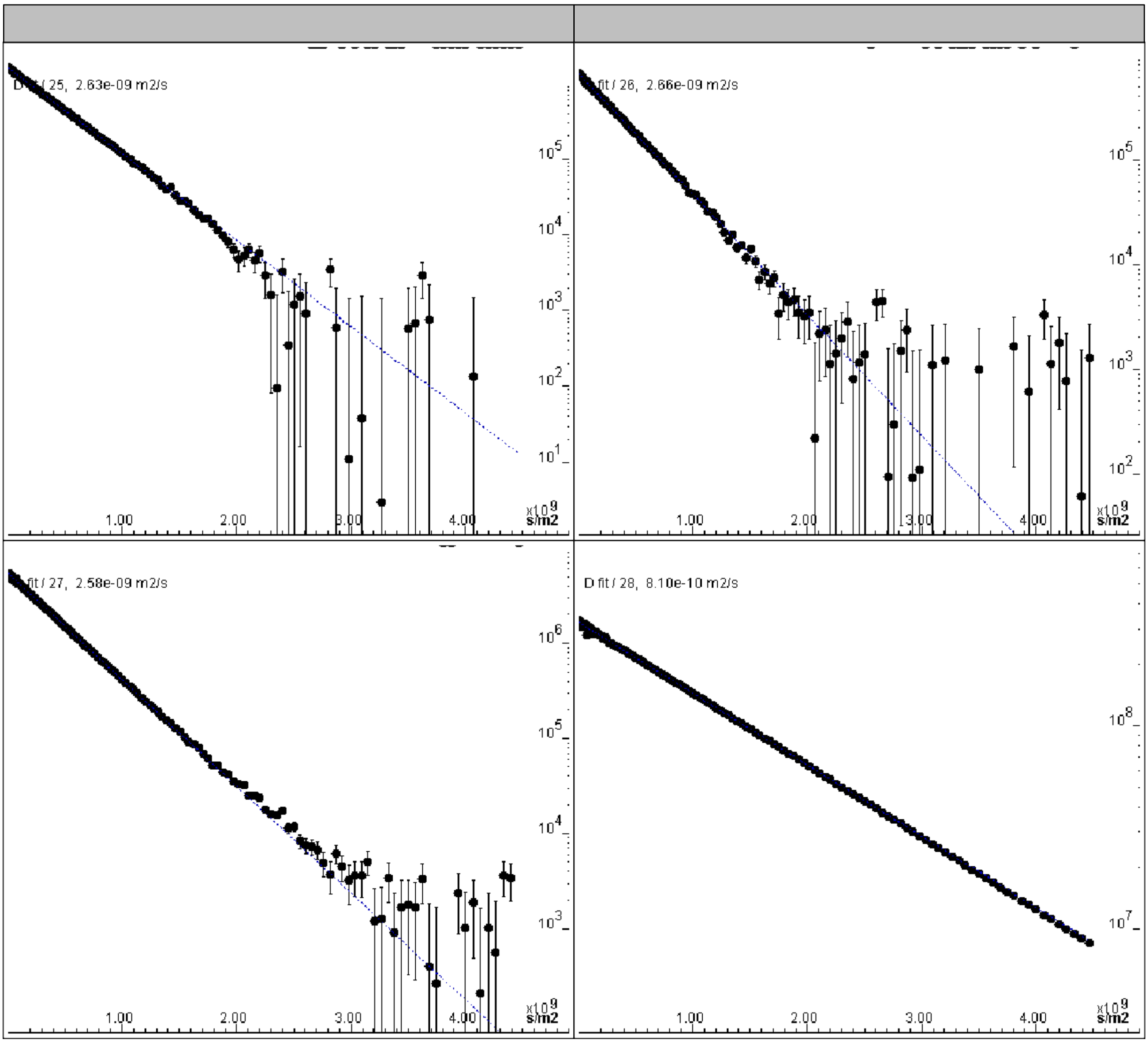

D fit / 25, 2.63e-09 m2/s
fit / 26, 2.66e-09 m2/s
fit / 27, 2.58e-09 m2/s
D fit / 28, 8.10e-10 m2/s
1.00
2.00
3.00
4.00
x10^9
s/m2

X-ray diffraction data

Supporting Table 3. Crystal data and structural refinement for **Na1D** and **Na2D**.

Identification code	**Na1D**	**Na2D**
CCDC number	**2288161**	**2288162**
Empirical formula	$C_{30}H_{38}N_3NaOSSi_2$	$C_{38}H_{35}N_6NaSSi_2$
Formula weight	567.86	686.95
Temperature/K	100.00	100.00
Crystal system	monoclinic	monoclinic
Space group	$P2_1/n$	$P2_1/c$
a/Å<br>b/Å<br>c/Å<br>α/°<br>β/°<br>γ/°	11.0634(4)<br>18.5248(7)<br>16.7331(7)<br>90<br>103.568(2)<br>90	14.9815(3)<br>13.4030(3)<br>20.4053(4)<br>90<br>92.8040(10)<br>90
Volume/Å^3	3333.7(2)	4092.42(15)
Z	4	4
ρ_{calc}g/cm^3	1.131	1.115
μ/mm^{-1}	0.207	1.615
F(000)	1208.0	1440.0
Crystal size/mm^3	0.11 × 0.07 × 0.05	0.10 × 0.05 × 0.04
Radiation	MoKα (λ = 0.71073)	CuKα (λ = 1.54178)
2Θ range for data collection/°	4.582 to 52	5.906 to 140.29
Index ranges	-13 ≤ h ≤ 13, -22 ≤ k ≤ 22, -18 ≤ l ≤ 20	-17 ≤ h ≤ 18, -16 ≤ k ≤ 15, -24 ≤ l ≤ 23
Reflections collected	30978	62124
Independent reflections	6514 [R_{int} = 0.0266, R_{sigma} = 0.0201]	7763 [R_{int} = 0.0724, R_{sigma} = 0.0417]
Data/restraints/parameters	6514/166/387	7763/251/565
Goodness-of-fit on F^2	1.049	1.085
Final R indexes [I>=2σ (I)]	R_1 = 0.0453, wR_2 = 0.1302	R_1 = 0.0819, wR_2 = 0.2130
Final R indexes [all data]	R_1 = 0.0505, wR_2 = 0.1353	R_1 = 0.1087, wR_2 = 0.2307
Largest diff. peak/hole / e Å^{-3}	0.91/-0.60	0.43/-0.52

(a) $R_1 = \sum||F_o|-|F_c||/\sum|F_o|$; $wR_2 = [\sum[w(F_o^2-F_c^2)^2/\sum[(wF_o^2)^2]]^{1/2}$; $w = 1/[\sigma^2(F_o^2)+(aP)^2+bP]$, where $P = (F_o^2+2F_c^2)/3$

(b) GooF = $S = [[\sum w(F_o^2-F_c^2)^2]/(m-n)]^{1/2}$, where m = number of reflexes and n = number of parameters

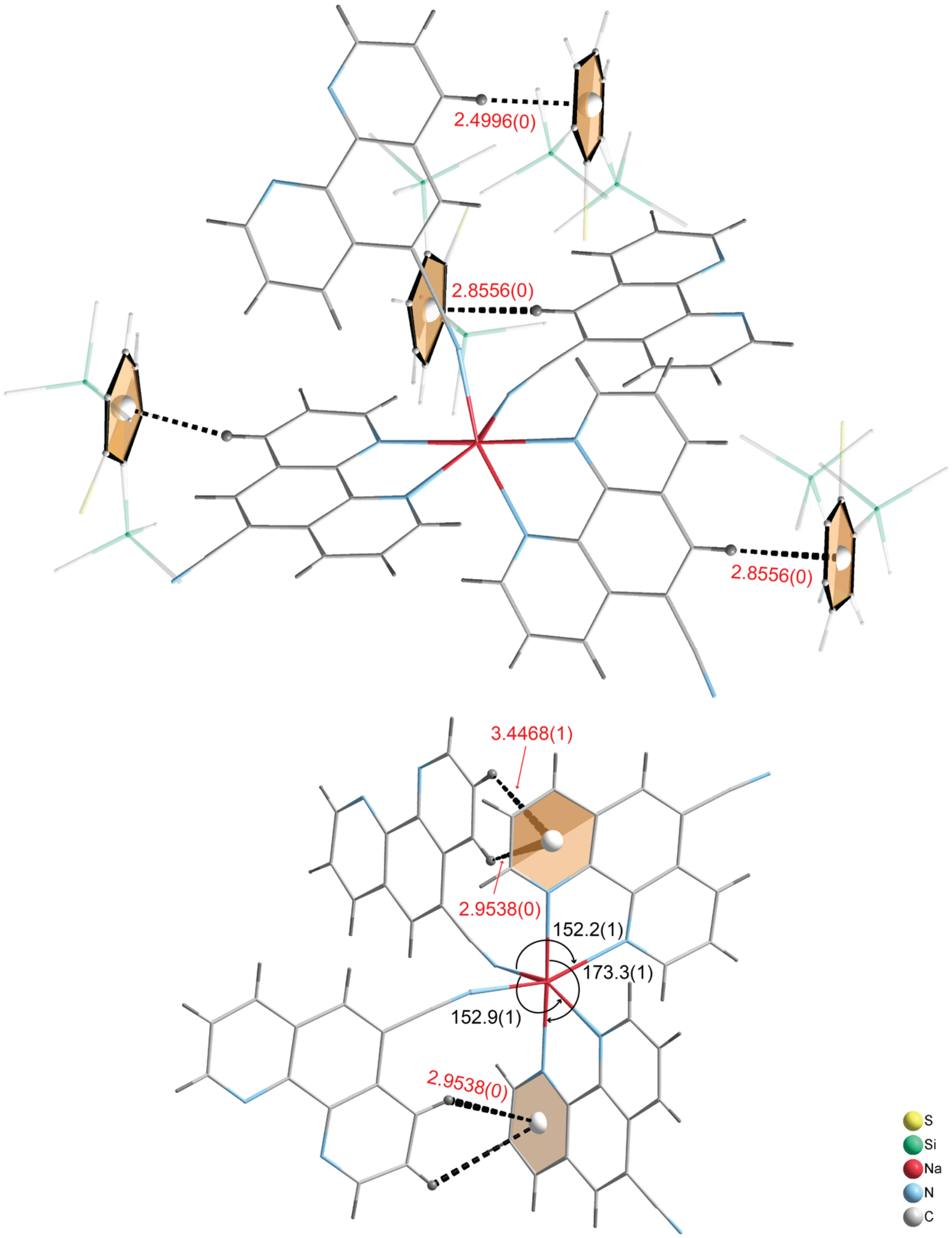

Supplementary Fig. S30. intermolecular (top) and intramolecular (bottom) non-covalent π···*H*–C interaction found for **Na2D**.

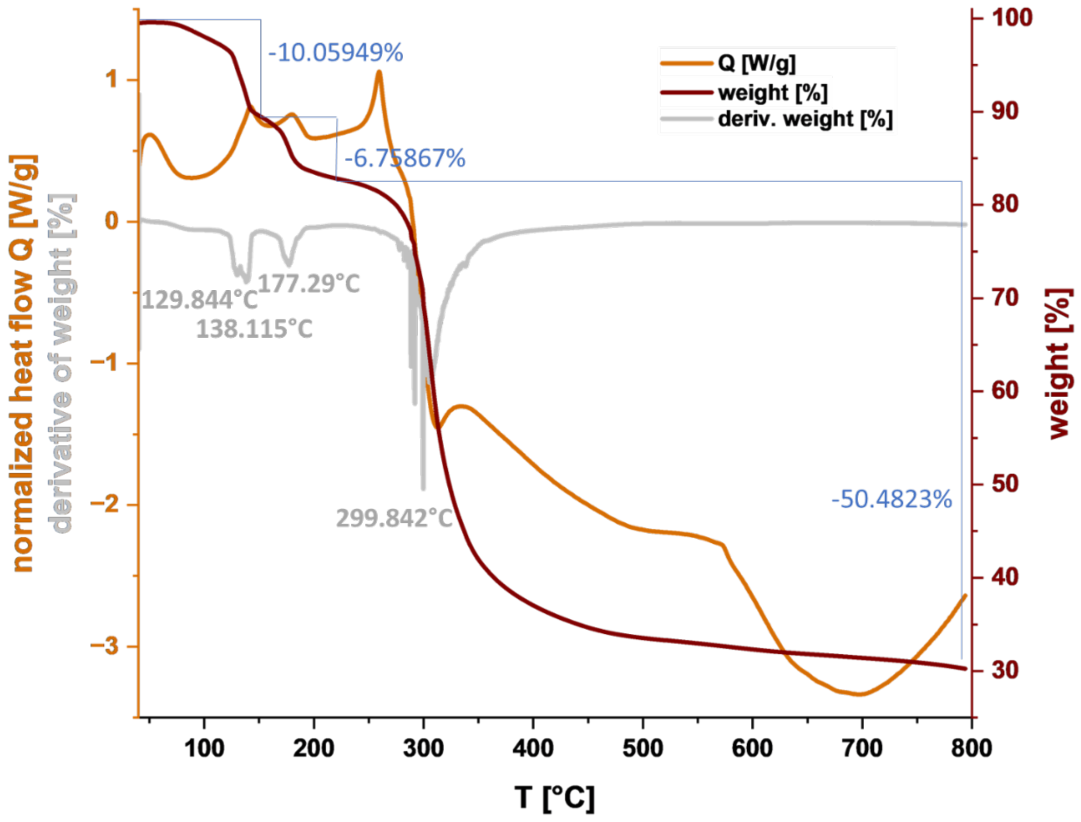

Supplementary Fig. S31. TGA results of **Na1D**.

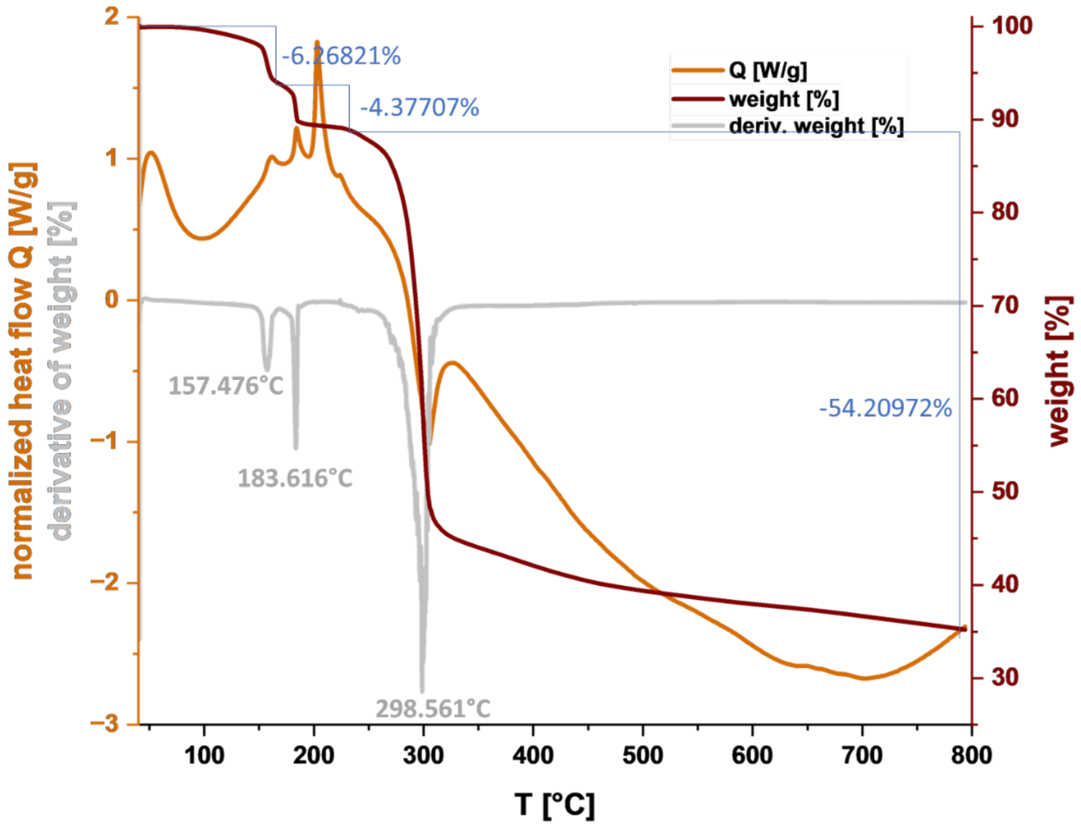

Supplementary Fig. S32. TGA results of **Na2D**.

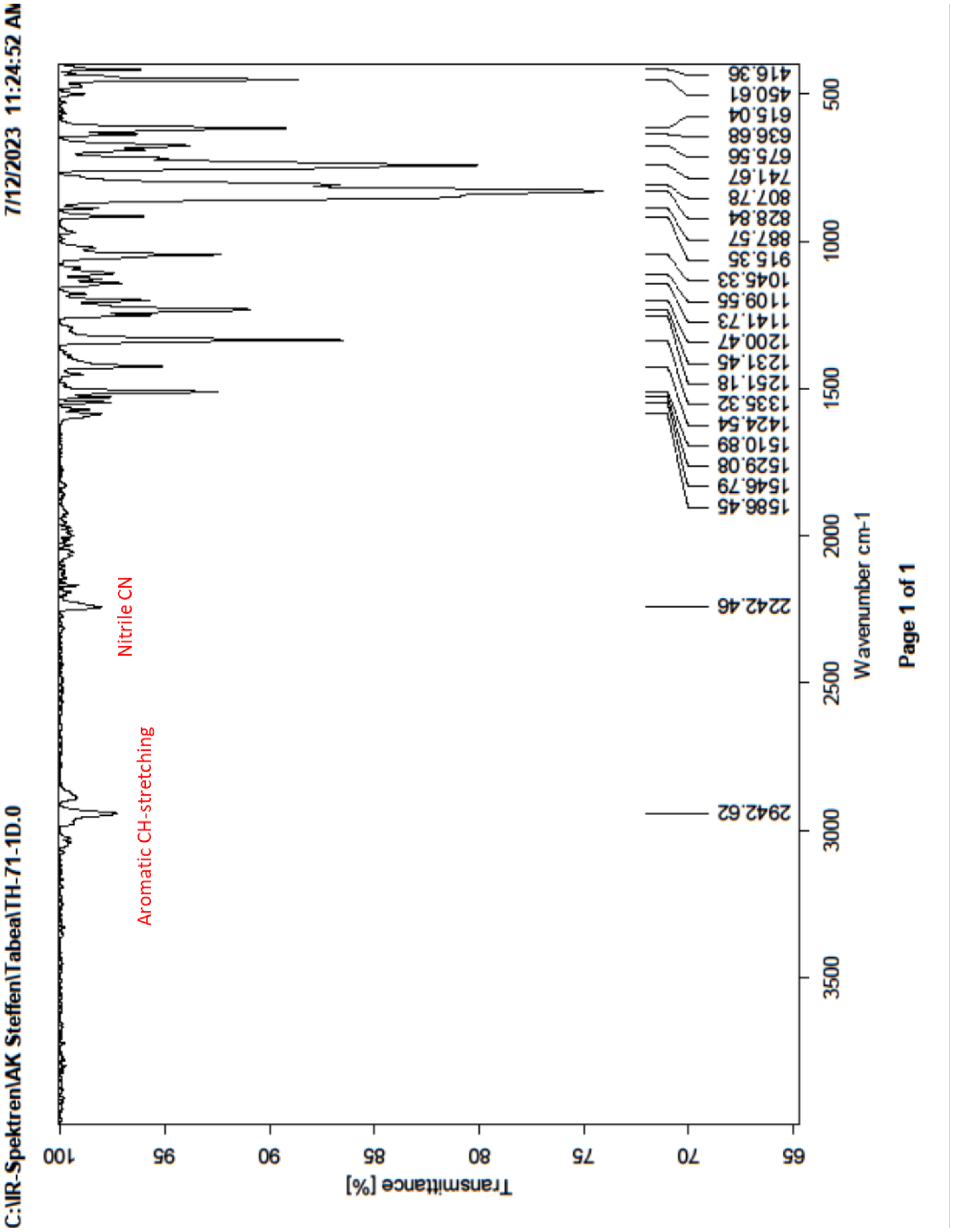

Supplementary Fig. 33. IR spectrum of **Na1D**.

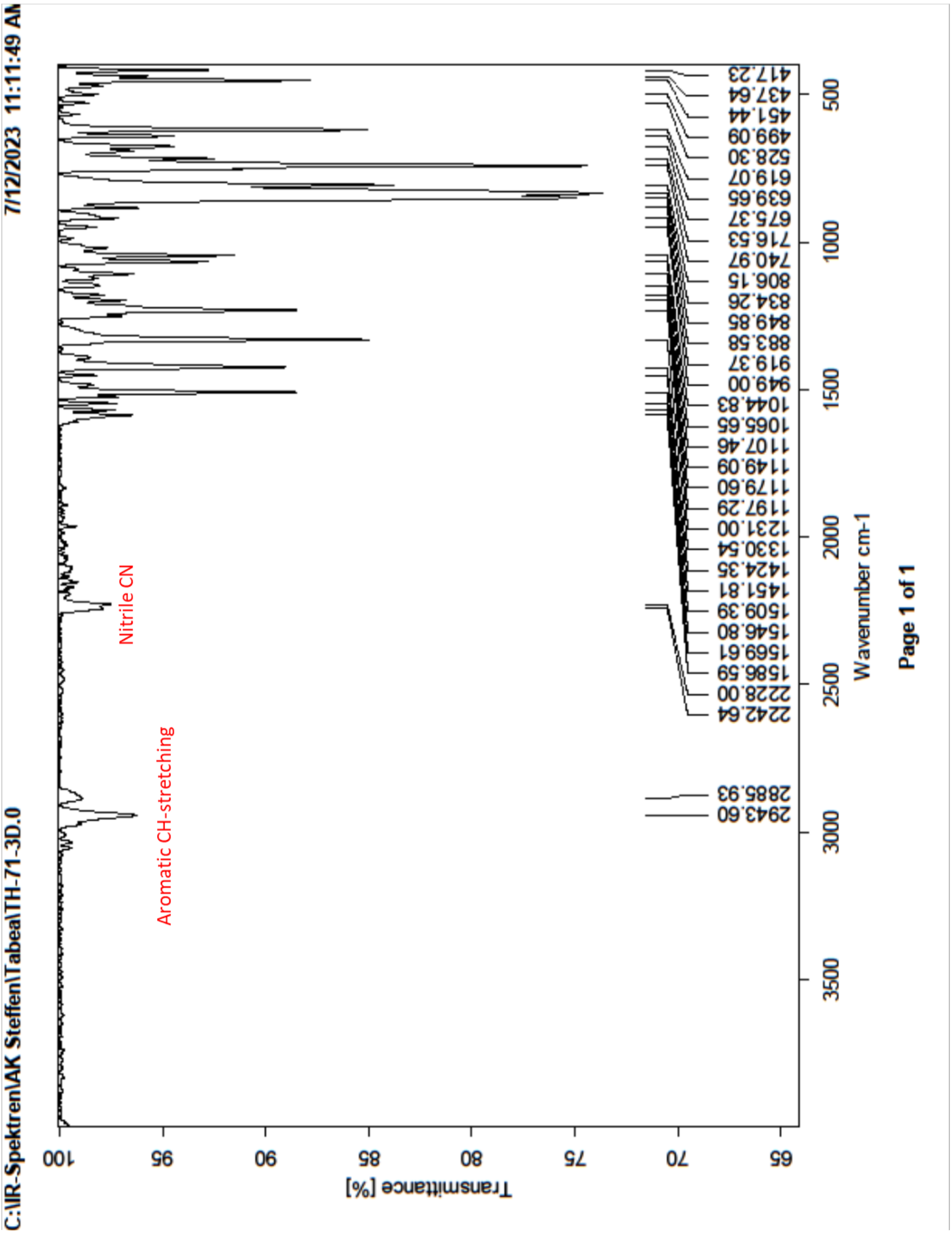

Supplementary Fig. 34. IR spectrum of **Na2D**.

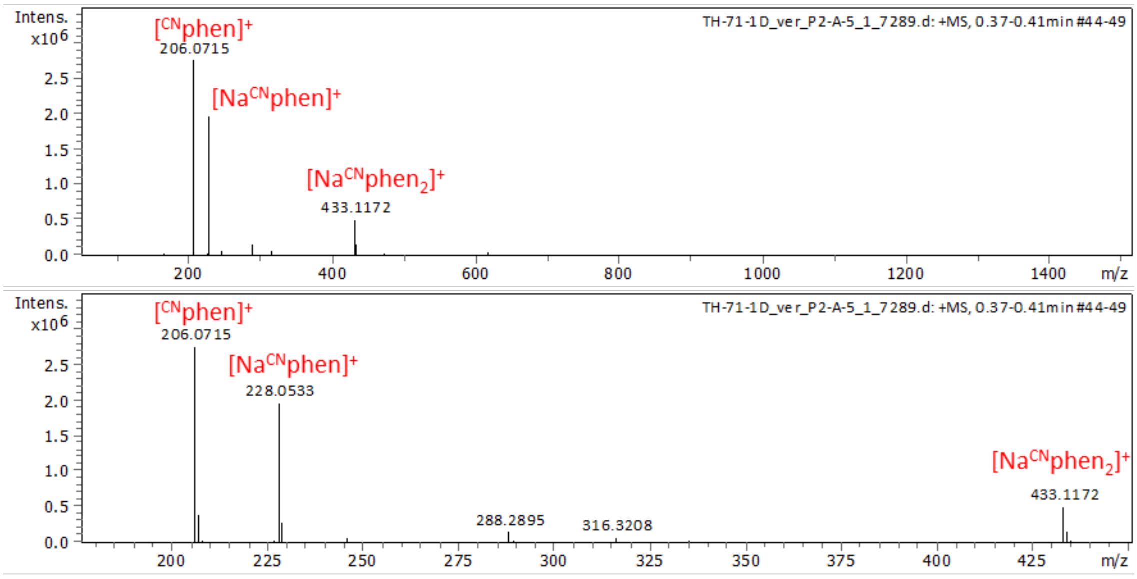

Supplementary Fig. 35. ESI-MS data in positive mode of **Na1D**. Full spectrum (top), zoom (bottom)

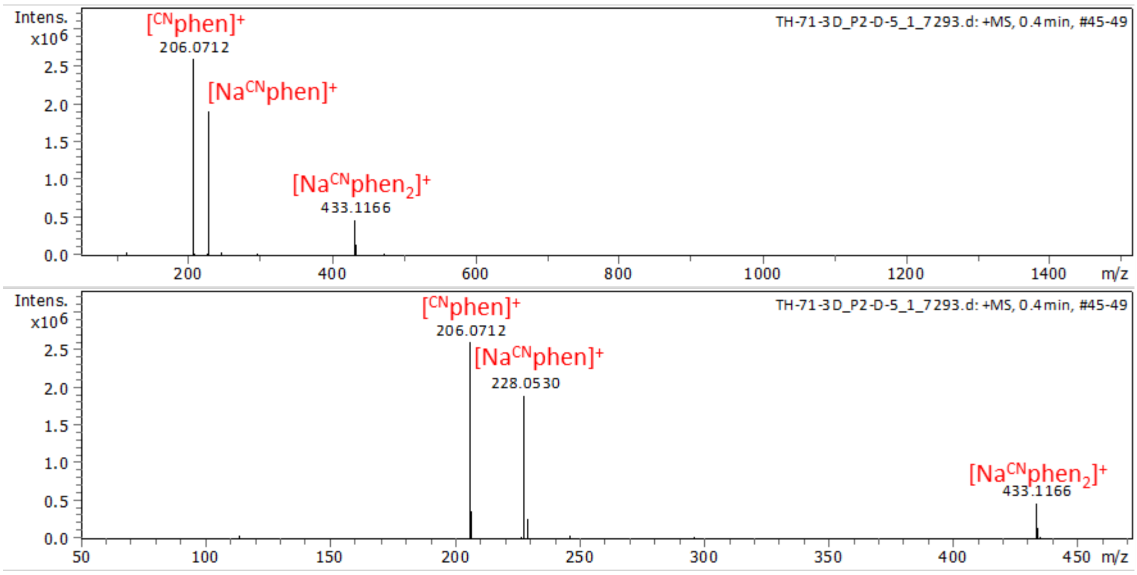

Supplementary Fig. 36. ESI-MS data in positive mode of **Na2D**. Full spectrum (top), zoom (bottom)

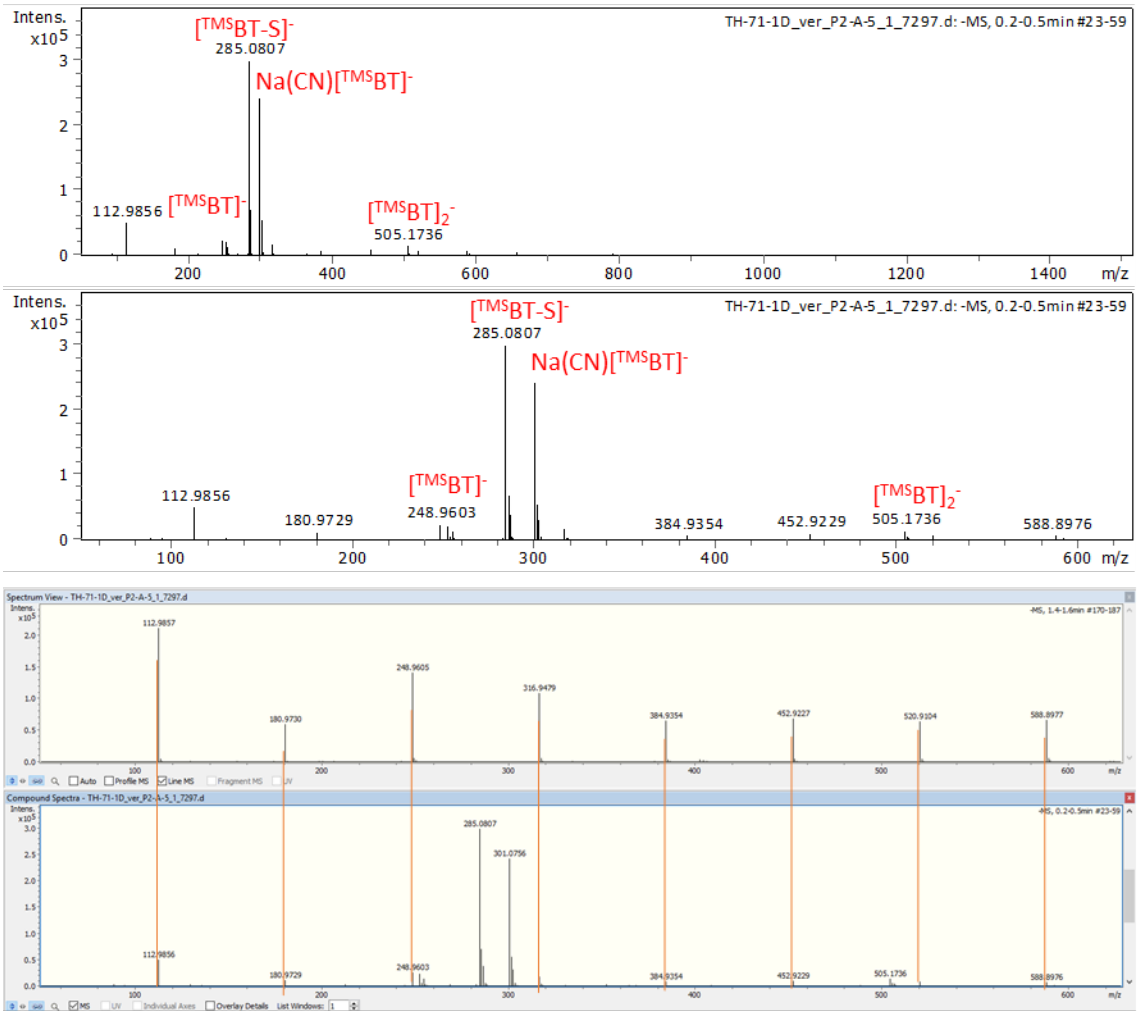

Supplementary Fig. 37. ESI-MS data in negative mode of **Na1D**. Full spectrum (top), zoom (middle), full spectrum vs. calibration curve (bottom).

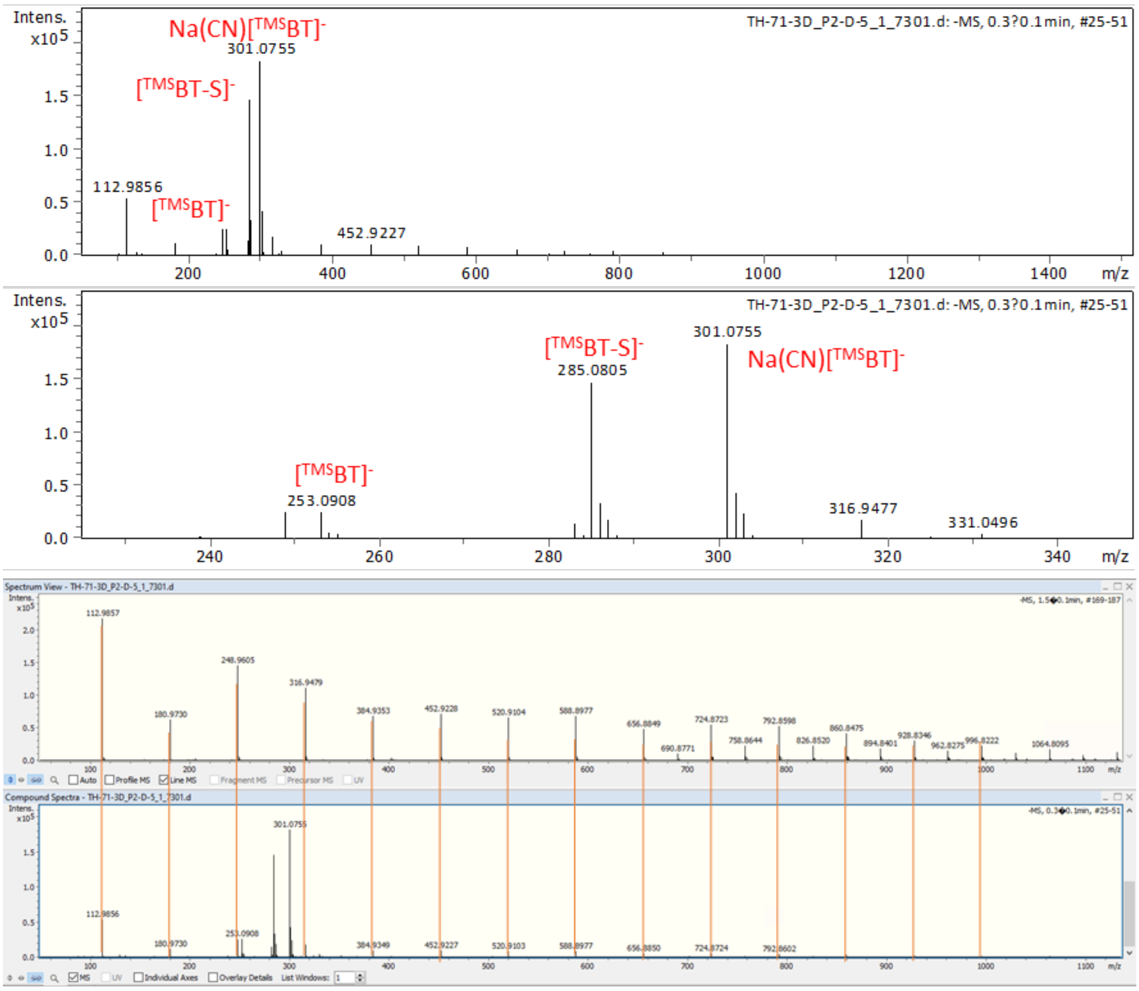

Supplementary Fig. 38. ESI-MS data in negative mode of **Na2D**. Full spectrum (top), zoom (middle), full spectrum vs. calibration curve (bottom).

Supporting Table 4. ESI-MS data of **Na1D** and **Na2D** with suggested assignments and their theoretical [m/z] distributions.

mode	**Na1D** [m/z]	**Na2D** [m/z]	Assigned compound	[m/z] theor. Mass/Charge	Fraction	Intensity
Positive mode	206.0715	206.0712	$[^{CN}phen]^+$ $[C_{13}H_8N_3]^+$	206.07182	0.8588939	100.00
				207.07182	0.1313124	15.29
				208.07182	0.0093677	1.09
				209.07182	0.0004138	0.05
				210.07182	0.0000211	0.00
	228.0533	228.053	$[Na^{CN}phen]^+$ $[NaC_{13}H_7N_3]^+$	228.05376	0.8590227	100.00
				229.05376	0.1312032	15.27
				230.05376	0.0093485	1.09
				231.05376	0.0004123	0.05
				232.05376	0.0000210	0.00
	433.1172	433.1166	$[Na(^{CN}phen)_2]^+$ $[Na(C_{13}H_7N_3)_2]^+$	433.11776	0.7379199	100.00
				434.11776	0.2254130	30.55
				435.11776	0.0332776	4.51
				436.11776	0.0031630	0.43
				437.11776	0.0002176	0.03
				438.11776	0.0000187	0.00
Negative mode	253.0908	253.0908	$[^{TMS}BT]^-$ $[S(C_6H_3(Si(CH_3)_3)_2)]^-$	253.09024	0.7074779	100.00
				254.09024	0.1715636	24.25
				255.09024	0.0977949	13.82
				256.09024	0.0177569	2.51
				257.09024	0.0046864	0.66
				258.09024	0.0006286	0.09
				259.09024	0.0000924	0.01
				260.09024	0.0000088	0.00
	285.0807	285.0805	$[^{TMS}BT\text{-}S]^-$ $[SS(C_6H_3(Si(CH_3)_3)_2]^-$	285.06232	0.6716088	100.00
				286.06232	0.1682421	25.05
				287.06232	0.1244914	18.54
				288.06232	0.0249599	3.72
				289.06232	0.0089207	1.33
				290.06232	0.0014307	0.21
				291.06232	0.0003137	0.05
				292.06232	0.0000397	0.01
				293.06232	0.0000056	0.00
	301.0756	301.0755	$Na(CN)[^{TMS}BT]^-$ $[(S(C_6H_2(Si(CH_3)_3)_2))NaCN]^-$	301.07526	0.6974370	100.00
				302.07526	0.1791433	25.69
				303.07526	0.0988615	14.17
				304.07526	0.0188955	2.71
				305.07526	0.0048770	0.70
				306.07526	0.0006868	0.10

				307.07526	0.0001004	0.01
				308.07526	0.0000100	0.00
	505.1736		[TMSBT]$_2^-$	505.17266	0.5006001	100.00
			[(S(C_6H_3(Si(CH_3)$_3$)$_2$))-	506.17266	0.2427162	48.49
			(S(C_6H_2(Si(CH_3)$_3$)$_2$))]$^-$	507.17266	0.1677936	33.52
				508.17266	0.0586626	11.72
				509.17266	0.0222773	4.45
				510.17266	0.0059710	1.19
				511.17266	0.0015775	0.32
				512.17266	0.0003378	0.07
				513.17266	0.0000668	0.01
				514.17266	0.0000115	0.00
				515.17266	0.0000017	0.00

Photophysical data

UV/Vis data

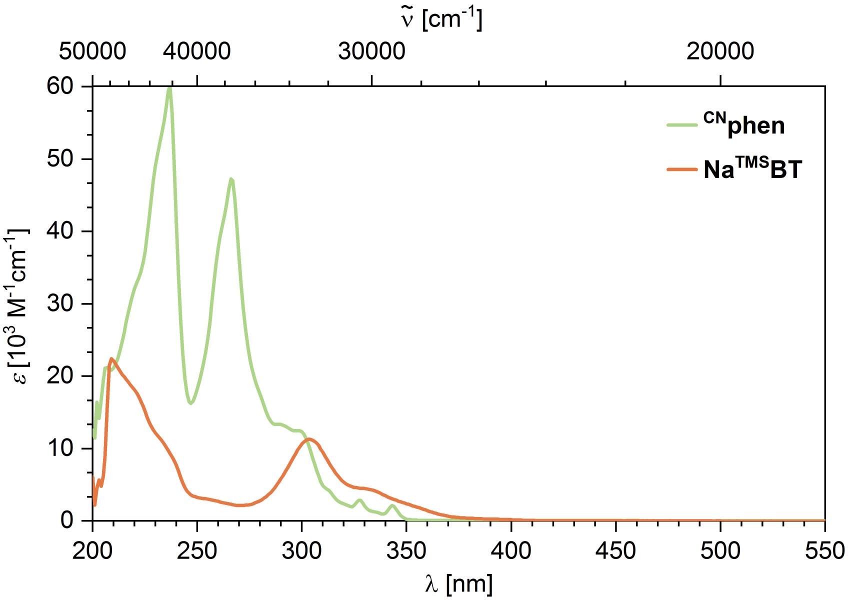

Supplementary Fig. 39. Steady-state UV-Vis profile of ^{CN}phen and Na^{TMS}BT molecules.

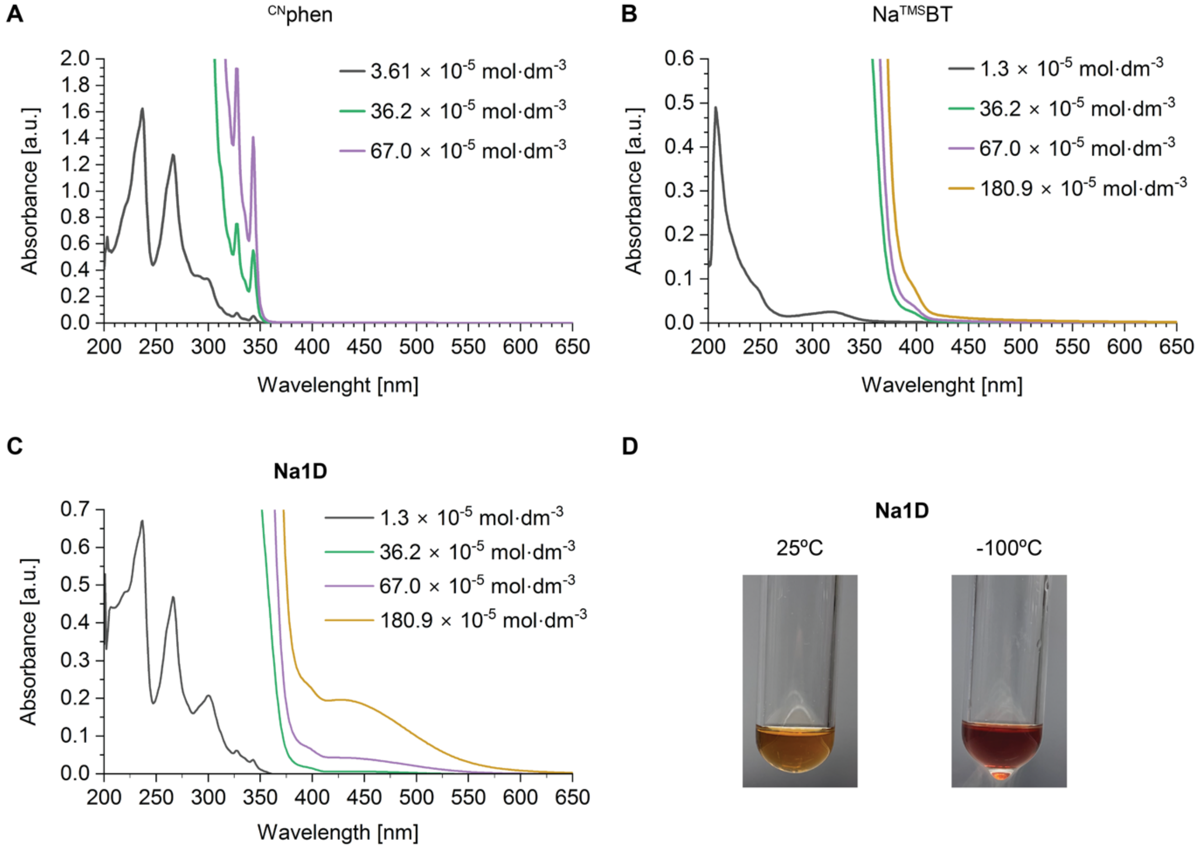

Supplementary Fig. 40. **A-C**, concentration-dependent absorption of ^{CN}phen, Na^{TMS}BT, and **Na1D**. **D**, Color change of **Na1D** solution upon cooling of the system.

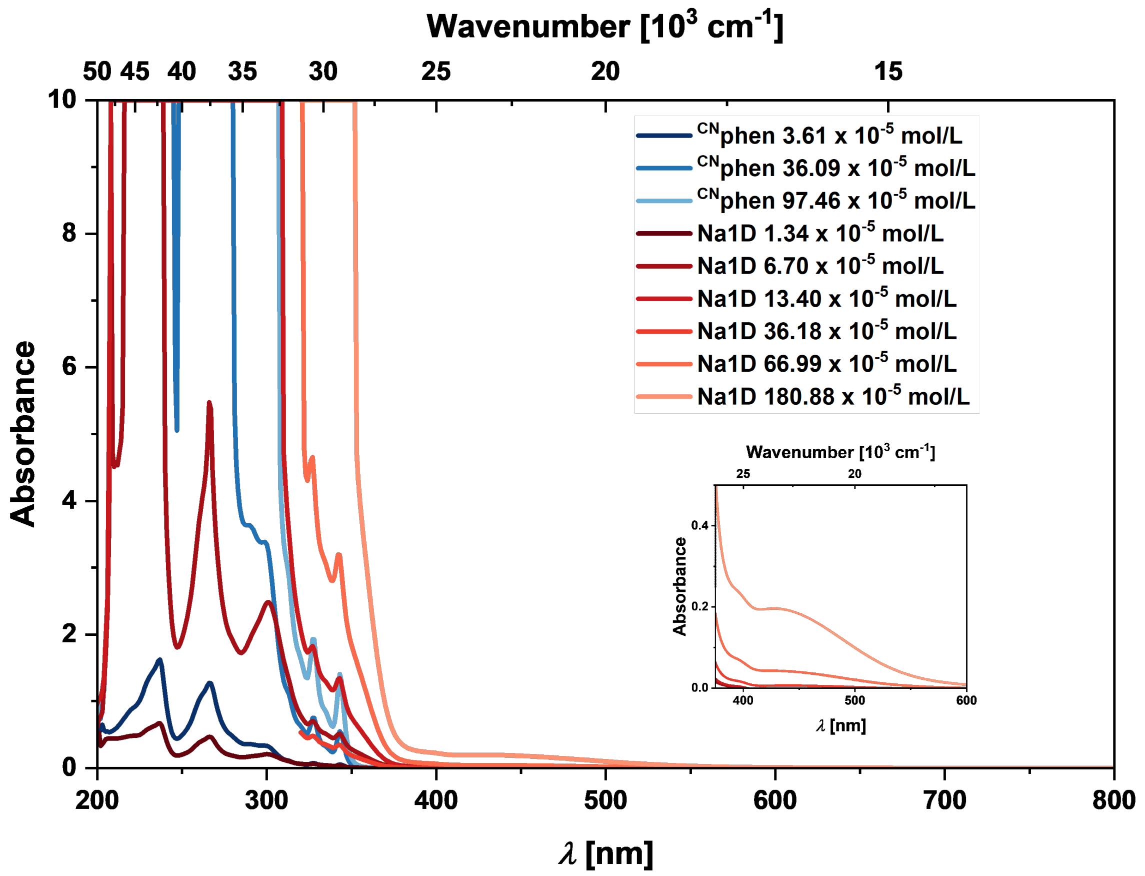

Supplementary Fig. 41. Absorbance of **Na1D** in THF at concentrations 0.134 µmol/L, 0.67 µmol/L, 1.34 µmol/L, 3.618 µmol/L, 6.699 µmol/L and 18.088 µmol/L (dark to light red) and absorbance of ^{CN}phen in THF at concentrations 0.361 µmol/L, 3.609 µmol/L and 9.746 µmol/L (dark to light blue).

Emission and excitation spectra

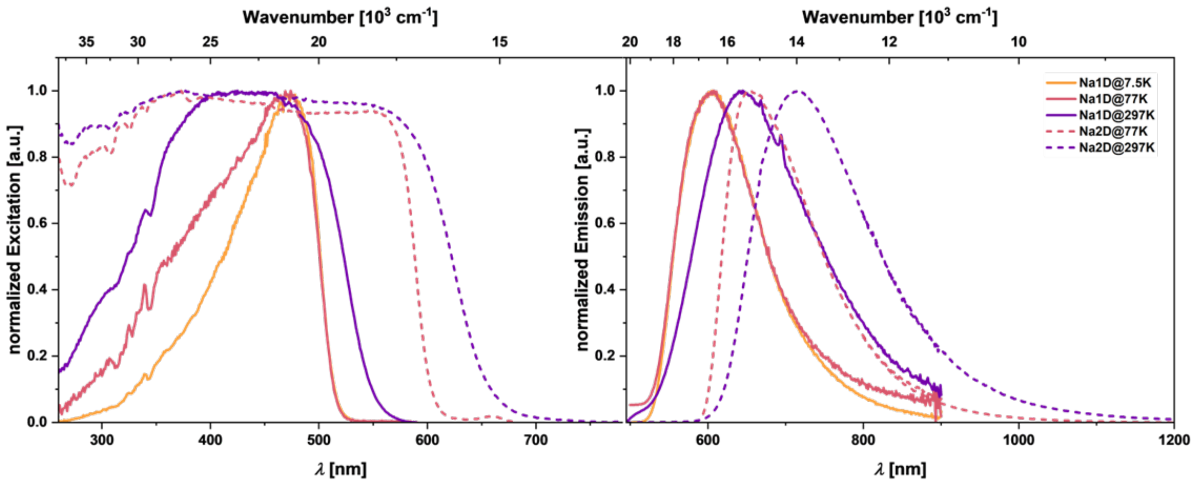

Supplementary Fig. 42. Normalized excitation spectra (left) and emission spectra (right) of crystalline **Na1D** (solid) and powder of **Na2D** (dashed) at 7.5 (orange), 77 (red) and 297 K (purple).

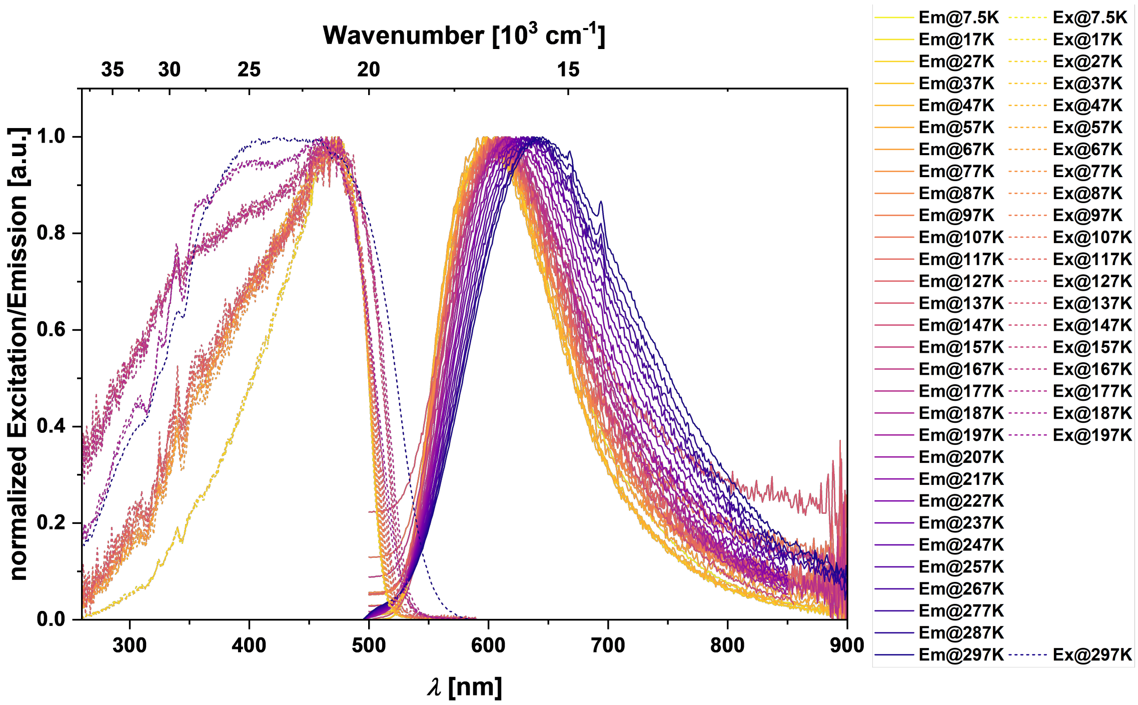

Supplementary Fig. 43. Normalized excitation (dashed, emission at 610 nm) and emission (solid, excitation at 470 nm) spectra of crystalline **Na1D** at temperatures between 7.5 (yellow) and 297 K (purple).

Time-resolved photophysical data

Supporting Table 6. Details of temperature-dependent lifetime measurement of **Na1D** in solid state.

T [K]	τ_1 [ns]	τ_1^{cont} [%]	τ_2 [ns]	τ_2^{cont} [%]	τ_3 [ns]	τ_3^{cont} [%]	τ_4 [ns]	τ_4^{cont} [%]	<τ> [ns]	χ^2
297	114	0.61	386	0.39					221	1.20
287	111	0.63	405	0.37					221	1.10
277	134	0.62	443	0.38					252	1.20
267	150	0.61	470	0.39					276	1.19
257	166	0.57	497	0.43					308	1.17
247	196	0.58	549	0.42					344	1.10
237	226	0.53	589	0.47					399	1.11
227	238	0.45	626	0.55					451	1.06
217	280	0.41	691	0.59					521	1.11
207	265	0.30	729	0.70					591	1.15
197	330	0.29	815	0.71					674	1.12
187	348	0.26	886	0.74					744	1.10
177	386	0.12	1013	0.88					936	1.01
167	333	0.10	1083	0.90					1007	1.10
157	292	0.12	1173	0.88					1066	1.08
147	877	0.63	1580	0.37					1138	1.14
137	1028	0.64	1993	0.36					1374	1.10
127	1140	0.64	2246	0.36					1537	1.13
117	1241	0.61	2473	0.39					1717	1.13
107	1173	0.45	2469	0.55					1883	1.12
97	834	0.29	2539	0.71					2045	1.27
87	417	0.27	2773	0.73					2143	1.24
77	1526	0.30	3564	0.70					2956	1.13
67	806	0.34	4217	0.66					3069	1.18
57	3281	0.58	7571	0.42					5086	1.28
47	3731	0.64	12269	0.36					6808	1.28
37	6284	0.84	23448	0.16					9034	1.30
27	13340	0.86	56253	0.12	170995	0.02	217	0.00	22561	1.11
17	43990	0.62	277933	0.29	1058826	0.08	186	0.01	236520	1.19
7	55998	0.46	372447	0.40	1380846	0.13	89	0.01	435453	1.23

[a] Decay traces were recorded using 450 nm VPL (297 - 37 K) or μs flash lamp (27 - 7 K) excitation source at emission 600 nm.

Supporting Table 7. Details of temperature-dependent lifetime measurement of **Na2D** in solid state.

T [K]	τ_1 [ns]	τ_1^{cont} [%]	τ_2 [ns]	τ_2^{cont} [%]	τ_3 [ns]	τ_3^{cont} [%]	τ_4 [ns]	τ_4^{cont} [%]	<τ> [ns]	χ^2
297 [a]	1	0.59	17	0.18	114	0.14	562	0.094	73	1.07
297	14	0.55	47	0.45					29	4.89
247	14	0.19	71	0.81					60	0.43
187	99	0.96	582	0.04					118	1.26
137	62	0.43	198	0.49	493	0.07			161	1.05
77	25	0.17	189	0.65	695	0.18			251	1.04
57	131	0.23	398	0.69	1443	0.08			423	1.02
47	145	0.31	533	0.65	2377	0.03			475	1.04
37	214	0.25	822	0.72	4453	0.02			758	1.11
27	895	0.70	2167	0.28	10411	0.01			1395	1.17
22	1114	0.65	3138	0.33	13385	0.02			2055	1.24
17	2400	0.75	8320	0.23	33707	0.02			4490	1.25
7	10644	0.27	109818	0.48	273716	0.25			124694	1.07

[a] Decay trace was recorded using high energy 365 nm VPLED excitation source at emission 700 nm.
[b] All other traces were recorded using 450 nm VPL excitation source at emission 700 - 640 nm.

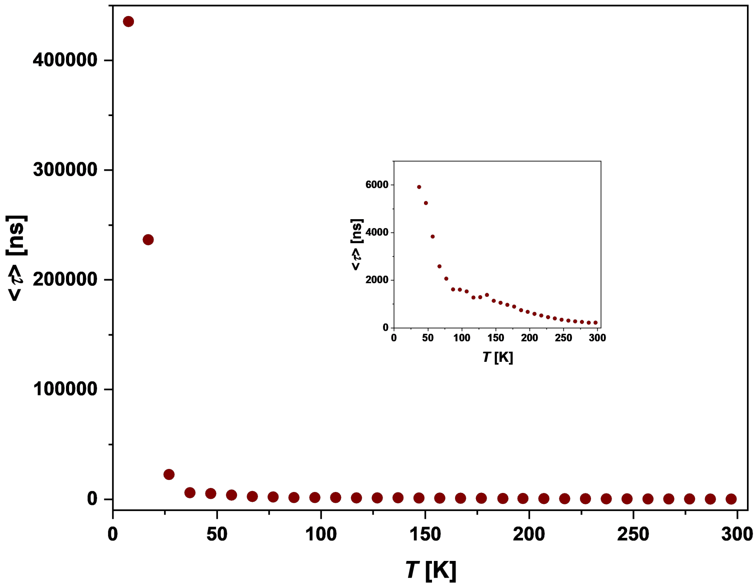

Supplementary Fig. 44. Emission lifetime of crystalline **Na1D** at temperatures between 7 and 297 K.

Additional photophysical data

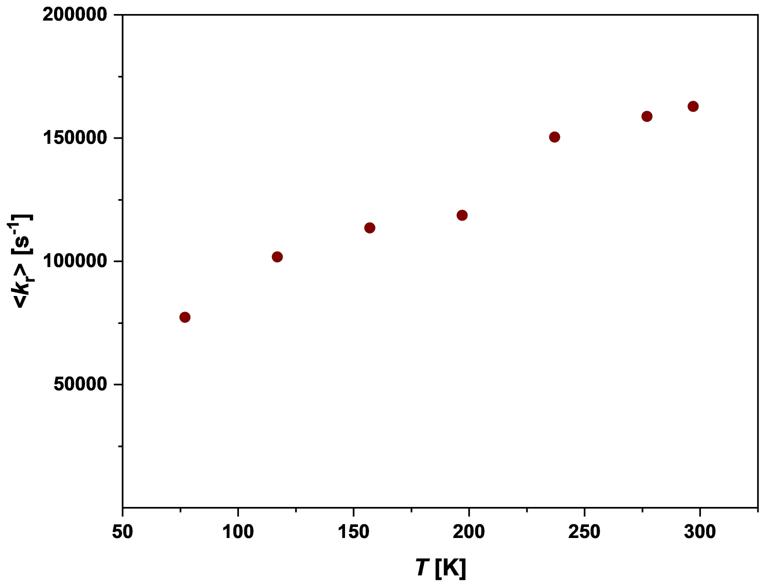

Supplementary Fig. 39. Radiative rate constants **Na1D** at different temperatures between 7 and 297 K

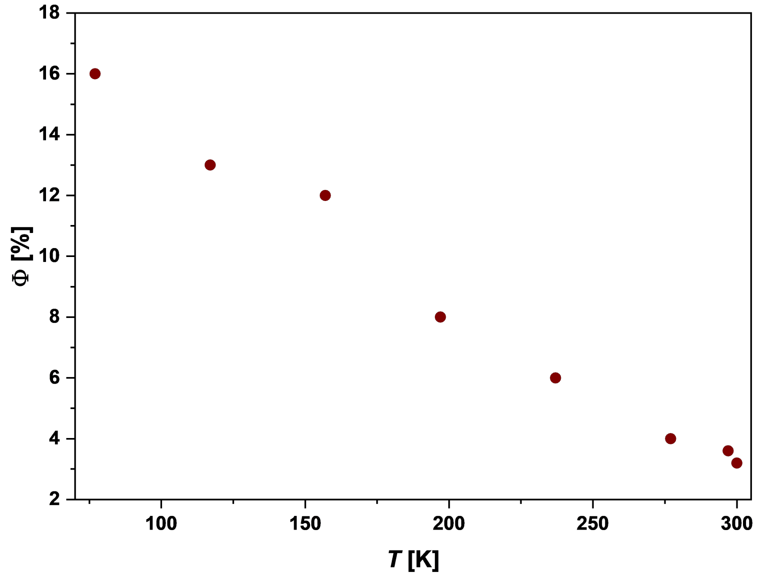

Supplementary Fig. 40. PLQY of crystalline Na1D between 77 K and 297 K.

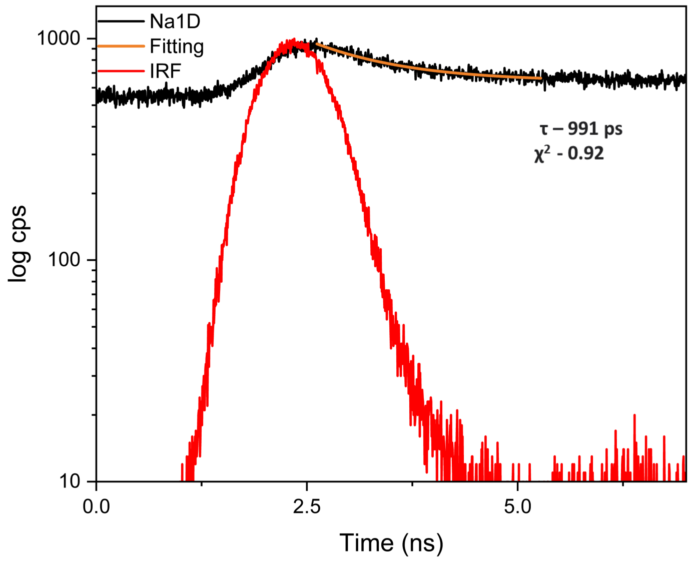

Supplementary Fig. 41. Prompt fluorescence lifetime of Na1D at 297 K.

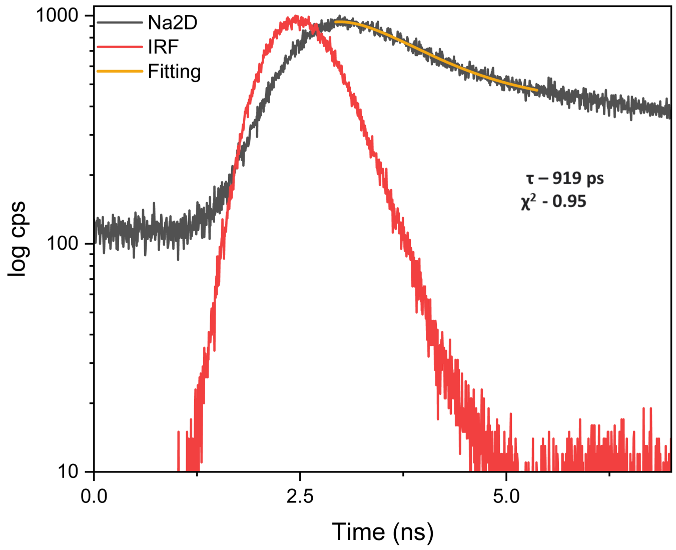

Supplementary Fig. 42. Prompt fluorescence lifetime of Na2D at 297 K.

Photoisomerization of (*E*)-stilbene

Supporting Table 8. (*E*)-stilbene→(*Z*)-stilbene photoisomerization with 470 nm irradiation.

**T [h]**	**c (Na1D) [mol/L]**	**c (Na2D) [mol/L]**	**conversion to (Z)-stilbene [%]**
0	1.4	0	0.1
2	1.4	0	1.0
6	1.4	0	11.6
12	1.4	0	49.9
0	0	1.4	0.1
2	0	1.4	1.5
6	0	1.4	11.4
12	0	1.4	67.7
12	0	0	0.8

Supporting Table 9. Control (*E*)-stilbene→(*Z*)-stilbene photoisomerization with ^{CN}phen and Na^{TMS}BT with 470 nm irradiation.

**T [h]**	**c (^{CN}phen) [mol/L]**	**c (Na^{TMS}BT) [mol/L]**	**conversion to (Z)-stilbene [%]**
12	1.4	0	2.2
12	0	1.4	19.4

DFT calculations

Difference densities

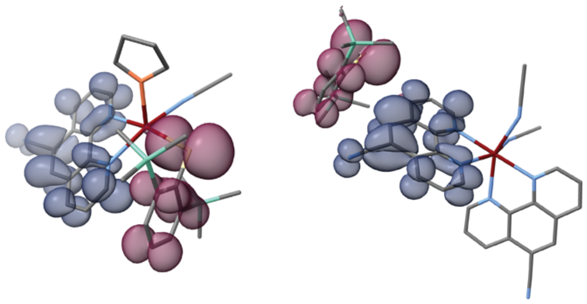

'Na1D' 'Na2D'

Supporting Fig. 49. TD-DFT difference densities. TD-DFT difference densities of the $S_0 \rightarrow T_1$ excitations of monomeric units isolated from X-ray diffraction data of **Na1D** and **Na2D**. Areas losing electron density are depicted in purple, and areas gaining in blue.

TD-DFT excitation data

Supporting Table 10. Singlet and triplet excitations of **Na1D**.

Excited state no.	singlets			triplets	
	E [eV]	λ [nm]	$f \times 10^{-3}$	E [eV]	λ [nm]
1	1.73	717.6	0.11	1.73	718.3
2	1.89	654.5	0.01	1.89	654.5
3	1.94	638.3	0.2	1.94	639.3
4	2.11	587.7	0.14	2.11	587.9
5	3.06	405.0	0.1	3.06	405.4
6	3.22	385.4	0.01	3.06	405.0
7	3.56	348.0	6.33	3.22	385.4
8	3.71	334.1	0.02	3.23	384.3
9	3.79	327.5	0.7	3.31	374.8
10	3.81	325.7	1.16	3.55	349.5
11	3.83	323.4	1.42	3.63	341.6
12	3.85	322.3	0.27	3.66	338.5
13	3.88	319.2	0.04	3.71	334.2
14	4.07	304.6	0.45	3.73	332.6
15	4.10	302.2	0.08	3.75	331.0
16	4.22	293.7	0.06	3.77	328.5
17	4.28	289.4	0.01	3.84	322.7
18	4.30	288.3	1.01	3.88	319.5
19	4.31	287.4	2.17	3.97	312.2
20	4.33	286.3	0.42	4.07	304.6
21	4.34	285.9	15.35	4.11	302.0
22	4.43	279.7	0.75	4.23	293.4
23	4.46	278.1	5.5	4.28	289.4
24	4.48	276.9	0.16	4.31	287.9
25	4.51	274.7	0.01	4.35	285.3
26	4.56	272.1	6.47	4.39	282.7
27	4.58	270.5	1.23	4.44	279.2
28	4.63	267.8	0.35	4.45	278.9
29	4.65	266.5	2.02	4.51	274.8
30	4.66	266.1	0.01	4.54	273.0
31	4.71	263.2	6.27	4.55	272.5

32	4.75	260.8	2.05	4.61	269.0
33	4.76	260.4	14.16	4.63	267.5
34	4.82	257.5	1.61	4.64	267.2
35	4.82	257.1	0.12	4.66	266.3
36	4.83	256.6	0.03	4.66	265.9
37	4.87	254.5	0.02	4.67	265.6
38	4.89	253.8	0.02	4.71	263.5
39	4.91	252.4	0.82	4.74	261.4
40	4.91	252.3	23.59	4.81	257.7
41	4.95	250.5	0.01	4.82	257.1
42	4.99	248.6	0.26	4.83	256.7
43	4.99	248.5	121.77	4.87	254.5
44	5.05	245.5	0.88	4.89	253.8
45	5.06	245.1	0.02	4.89	253.4
46	5.07	244.4	0.05	4.91	252.5
47	5.14	241.2	0.11	4.94	250.8
48	5.17	239.6	0.19	4.98	249.1
49	5.28	234.6	0.75	4.99	248.7
50	5.32	233.0	0.19	1.73	245.3

Supporting Table 11. Singlet and triplet excitations of **Na2D**.

Excited state no.	singlets			triplets	
	E [eV]	λ [nm]	ƒ×10^{-3}	E [eV]	λ [nm]
1	1.31	948.3	13.59	1.28	971.6
2	1.38	899.6	0.02	1.38	899.6
3	1.53	809.8	0.46	1.53	812.0
4	1.60	777.2	0.00	1.60	777.2
5	1.78	694.7	0.40	1.78	695.2
6	1.87	663.8	7.22	1.85	671.2
7	2.00	619.2	0.07	2.00	619.3
8	2.09	594.2	1.67	2.08	595.7
9	2.74	452.1	0.17	2.74	452.8
10	2.76	448.9	0.09	2.76	449.1
11	2.97	417.7	0.02	2.89	429.4
12	2.99	414.8	0.05	2.93	422.8
13	3.25	380.9	11.10	2.96	419.4
14	3.40	364.3	1.06	2.97	417.7
15	3.44	360.5	3.87	2.99	414.8
16	3.46	358.7	8.75	3.29	377.2
17	3.49	355.0	32.84	3.36	368.7
18	3.55	349.4	0.01	3.43	361.5
19	3.57	347.4	0.39	3.44	360.1
20	3.66	338.4	0.05	3.45	359.2
21	3.92	316.2	0.20	3.55	349.4
22	3.93	315.4	1.48	3.55	348.8
23	3.96	313.3	0.01	3.66	338.5
24	3.99	310.4	0.55	3.69	335.7
25	4.01	309.1	0.01	3.73	332.4
26	4.03	307.8	2.82	3.74	331.8
27	4.07	304.9	1.69	3.75	330.2
28	4.08	304.0	0.31	3.86	321.6
29	4.12	300.6	13.32	3.91	317.4
30	4.18	296.7	0.04	3.92	316.5
31	4.18	296.4	58.54	3.92	316.3
32	4.23	293.1	10.17	3.96	313.3

33	4.25	291.7	0.43	3.99	310.7
34	4.26	290.7	76.09	4.01	309.2
35	4.27	290.3	46.14	4.02	308.5
36	4.28	290.0	236.61	4.03	307.5
37	4.29	288.8	184.80	4.06	305.2
38	4.30	288.5	58.26	4.07	304.4
39	4.32	287.0	4.55	4.10	302.2
40	4.32	286.9	6.92	4.15	298.5
41	4.34	285.7	0.01	4.18	296.7
42	4.36	284.5	4.11	4.25	291.8
43	4.37	283.5	20.27	4.25	291.6
44	4.37	283.4	1.60	4.27	290.5
45	4.38	282.8	0.72	4.27	290.1
46	4.41	281.4	0.09	4.30	288.3
47	4.42	280.5	0.08	4.32	287.2
48	4.44	279.4	2.03	4.32	286.8
49	4.49	275.9	0.17	4.34	285.7
50	4.50	275.5	0.47	4.35	284.8

XYZ coordinates

Na1D

16	5.584390000	17.053607000	5.191332000
14	7.678345000	16.094030000	7.600675000
14	6.247376000	16.587656000	1.989062000
11	3.788206000	14.909510000	5.227869000
8	2.900531000	15.382159000	7.333107000
7	2.435988000	12.852267000	4.994955000
7	5.137407000	12.875992000	4.635350000
7	1.663148000	7.138090000	3.651749000
6	7.121890000	16.333338000	4.748184000
6	8.046418000	15.927434000	5.761457000
6	2.302750000	10.508236000	4.373655000
6	7.451705000	16.149576000	3.370620000
6	3.031061000	11.698501000	4.593027000
6	4.369849000	9.328175000	3.795282000
1	4.896498000	8.419844000	3.496157000
6	5.125713000	10.525479000	4.001299000
6	9.248740000	15.327574000	5.359621000
1	9.959914000	14.995182000	6.121264000
6	4.471046000	11.711662000	4.403092000
6	0.911254000	10.532313000	4.571692000
1	0.317051000	9.631314000	4.404579000
6	9.579968000	15.141041000	4.025813000
1	10.527932000	14.674065000	3.744616000
6	6.523513000	10.555313000	3.829440000

1	7.052455000	9.652200000	3.515636000
6	8.676693000	15.560658000	3.048312000
1	8.941296000	15.408061000	1.998316000
6	2.281910000	8.096411000	3.786789000
6	3.025550000	9.318535000	3.975569000
6	1.128681000	12.831515000	5.190677000
1	0.675847000	13.772953000	5.521727000
6	6.454203000	12.862293000	4.472811000
1	6.973927000	13.803848000	4.666793000
6	7.184249000	11.733724000	4.070808000
1	8.266846000	11.816387000	3.957514000
6	5.877928000	18.418068000	1.881487000
1	5.608469000	18.806642000	2.873860000
1	5.039606000	18.607114000	1.189137000
1	6.753747000	18.979487000	1.513549000
6	0.323518000	11.703123000	4.986790000
1	-0.752450000	11.765877000	5.159651000
6	7.056664000	16.086912000	0.364207000
1	8.018635000	16.599902000	0.197406000
1	6.390531000	16.350130000	-0.475562000
1	7.239655000	15.000141000	0.313633000
6	4.645067000	15.618969000	2.082918000
1	4.817559000	14.604751000	2.479552000
1	4.207507000	15.514888000	1.074772000
1	3.907061000	16.119010000	2.725120000

6	9.226096000	15.576982000	8.536371000
1	9.493859000	14.524132000	8.346446000
1	9.056079000	15.683512000	9.621745000
1	10.097655000	16.200024000	8.273884000
6	6.326478000	14.909965000	8.118649000
1	5.360720000	15.181136000	7.669868000
1	6.207784000	14.906513000	9.216229000
1	6.574151000	13.881864000	7.803758000
6	2.952045000	16.708432000	9.278164000
1	2.454694000	17.558685000	9.765557000
1	4.012799000	16.714926000	9.579043000
6	2.841960000	16.747310000	7.773600000
1	1.879275000	17.189968000	7.452860000
1	3.655835000	17.292376000	7.272606000
6	7.276140000	17.839870000	8.129948000
1	8.099494000	18.525521000	7.864664000
1	7.144216000	17.884984000	9.225581000
1	6.362424000	18.199025000	7.639011000
6	2.517011000	14.536087000	8.425369000
1	3.330715000	13.801249000	8.564429000
1	1.618786000	13.963142000	8.134404000
6	2.307623000	15.370784000	9.593435000
1	2.708019000	14.919375000	10.514232000
1	1.229656000	15.526612000	9.772771000
7	1.902428000	16.400610000	4.487084000

7	1.129320000	22.114383000	3.142315000
7	-1.572099000	22.138080000	3.501914000
6	1.262669000	19.770528000	3.764257000
6	0.534302000	20.960699000	3.544559000
6	-0.804376000	18.590529000	4.342953000
1	-1.330801000	17.681790000	4.642034000
6	-1.560295000	19.787741000	4.136607000
6	-0.905684000	20.973845000	3.734490000
6	2.654163000	19.794616000	3.566213000
1	3.255751000	18.898607000	3.742267000
6	-2.958096000	19.817557000	4.308459000
1	-3.488243000	18.914791000	4.621535000
6	1.283621000	17.358865000	4.351783000
6	0.539923000	18.580903000	4.162669000
6	2.436629000	22.093639000	2.946599000
1	2.897329000	23.031418000	2.612296000
6	-2.888895000	22.124364000	3.664456000
1	-3.416147000	23.066089000	3.469459000
6	-3.618888000	20.995871000	4.066768000
1	-4.702812000	21.068923000	4.181001000
6	3.241845000	20.965340000	3.150794000
1	4.318175000	21.019596000	2.976733000

Na2D

16	11.109964000	5.310345000	9.713773000
14	9.037773000	4.503826000	12.235996000
14	12.315334000	2.974063000	7.671752000
6	10.203254000	1.107895000	10.564914000
6	10.694580000	3.593029000	10.015573000
6	9.855946000	3.276287000	11.109901000
6	11.207152000	2.592162000	9.155125000
6	10.893273000	1.279903000	9.415501000
6	9.570134000	1.888991000	11.317260000
6	7.995887000	3.566193000	13.482333000
6	13.990972000	3.639357000	8.201713000
6	11.510187000	4.043431000	6.363496000
6	10.263338000	5.523179000	13.222211000
6	7.871350000	5.609640000	11.284009000
6	12.688000000	1.333198000	6.816971000
1	11.253852000	0.435795000	8.829365000
1	10.047622000	0.032579000	10.795166000
1	8.957935000	1.546960000	12.151187000
1	7.208204000	2.964673000	12.998331000
1	8.600289000	2.888352000	14.108964000
1	7.496892000	4.283302000	14.156957000
1	13.910342000	4.663997000	8.590284000
1	14.422067000	3.003690000	8.992707000
1	14.690215000	3.637229000	7.347226000

1	10.617493000	3.538325000	5.956010000
1	11.198468000	5.008622000	6.787402000
1	12.207292000	4.222868000	5.526590000
1	10.914456000	4.870434000	13.829571000
1	10.901963000	6.102397000	12.540246000
1	9.741364000	6.213179000	13.907615000
1	8.418113000	6.131233000	10.484840000
1	7.068210000	5.011018000	10.820468000
1	7.396977000	6.359234000	11.941401000
1	13.229868000	0.631727000	7.473700000
1	11.771398000	0.828086000	6.467837000
1	13.322544000	1.517016000	5.932792000
7	16.930765000	6.173396000	16.695397000
6	16.224120000	7.057936000	16.807823000
11	17.934487000	4.261067000	15.307762000
7	19.719071000	4.196400000	16.996304000
7	19.474972000	2.322057000	15.036991000
7	24.194145000	-0.850898000	16.535850000
6	20.660328000	3.228750000	16.926648000
6	19.857201000	5.107440000	17.968816000
6	21.749703000	3.146682000	17.809116000
6	20.496131000	2.209176000	15.874608000
6	22.689464000	2.066458000	17.708845000
6	20.939682000	5.119184000	18.869662000
6	21.447178000	1.155754000	15.788615000

6	22.534455000	1.108506000	16.744466000
6	23.470722000	0.006833000	16.654379000
6	21.883736000	4.139471000	18.804078000
6	19.332348000	1.402964000	14.095046000
6	21.262205000	0.193803000	14.763092000
6	20.184716000	0.325450000	13.913254000
1	23.518858000	2.016703000	18.416434000
1	18.469047000	1.529832000	13.431265000
1	20.001675000	-0.392413000	13.111918000
1	21.965354000	-0.635459000	14.658226000
1	19.079532000	5.874809000	18.021917000
1	20.992808000	5.908463000	19.622861000
1	22.729104000	4.105003000	19.495534000
7	16.179466000	4.381245000	13.560137000
7	16.240971000	2.506767000	15.515993000
7	19.253047000	5.851442000	14.031509000
6	15.177523000	3.472490000	13.584245000
6	14.141185000	3.407144000	12.658922000
6	16.146765000	5.276936000	12.576079000
7	11.534914000	-0.527153000	13.872118000
6	15.250777000	2.425312000	14.657920000
6	15.109025000	5.334910000	11.618195000
6	14.242444000	1.454900000	14.690157000
6	14.104686000	4.427501000	11.630074000
6	19.976434000	6.709295000	13.913630000

6	13.128458000	2.383109000	12.719671000
6	13.212596000	1.427108000	13.693515000
6	16.299639000	1.562839000	16.459270000
6	14.292725000	0.502901000	15.712916000
6	12.241520000	0.357503000	13.760362000
6	15.339050000	0.587021000	16.617866000
1	12.329151000	2.379395000	11.976889000
1	16.967491000	6.000812000	12.546643000
1	15.134834000	6.125102000	10.863302000
1	13.268216000	4.477927000	10.922246000
1	17.148556000	1.619474000	17.149583000
1	15.427502000	-0.132019000	17.435091000
1	13.530730000	-0.272865000	15.801362000
6	15.267612000	8.255239000	16.960001000
1	14.882651000	8.522258000	15.966535000
1	14.441734000	7.962466000	17.622091000
1	15.823975000	9.097493000	17.392744000
6	20.963761000	7.880150000	13.752739000
1	21.355462000	8.145272000	14.743975000
1	21.781442000	7.561848000	13.092336000
1	20.429199000	8.733057000	13.313516000

References

1 Armarego, W. L. F. *Purification of laboratory chemicals*. Eighth edition. edn, (Elsevier, 2017).

2 Fulmer, G. R. *et al.* NMR Chemical Shifts of Trace Impurities: Common Laboratory Solvents, Organics, and Gases in Deuterated Solvents Relevant to the Organometallic Chemist. *Organometallics* **29**, 2176-2179 (2010).

3 Sheldrick, G. SHELXT - Integrated space-group and crystal-structure determination. *Acta Crystallographica Section A* **71**, 3-8 (2015).

4 Sheldrick, G. Crystal structure refinement with SHELXL. *Acta Crystallographica Section C* **71**, 3-8 (2015).

5 Dolomanov, O. V., Bourhis, L. J., Gildea, R. J., Howard, J. A. K. & Puschmann, H. OLEX2: a complete structure solution, refinement and analysis program. *J. Appl. Crystallogr.* **42**, 339-341 (2009).

6 Spek, A. PLATON SQUEEZE: a tool for the calculation of the disordered solvent contribution to the calculated structure factors. *Acta Crystallographica Section C* **71**, 9-18 (2015).

7 Neese, F. Software update: The ORCA program system—Version 5.0. *WIREs Computational Molecular Science* **12**, e1606 (2022).

8 Vosko, S. H., Wilk, L. & Nusair, M. Accurate spin-dependent electron liquid correlation energies for local spin density calculations: a critical analysis. *Can. J. Phys.* **58**, 1200-1211 (1980).

9 Lee, C., Yang, W. & Parr, R. G. Development of the Colle-Salvetti correlation-energy formula into a functional of the electron density. *Physical Review B* **37**, 785-789 (1988).

10 Becke, A. D. Density-functional thermochemistry. III. The role of exact exchange. *J. Chem. Phys.* **98**, 5648-5652 (1993).

11 Stephens, P. J., Devlin, F. J., Chabalowski, C. F. & Frisch, M. J. Ab Initio Calculation of Vibrational Absorption and Circular Dichroism Spectra Using Density Functional Force Fields. *The Journal of Physical Chemistry* **98**, 11623-11627 (1994).

12 Weigend, F. & Ahlrichs, R. Balanced basis sets of split valence, triple zeta valence and quadruple zeta valence quality for H to Rn: Design and assessment of accuracy. *Phys. Chem. Chem. Phys.* **7**, 3297-3305 (2005).

13 Grimme, S., Ehrlich, S. & Goerigk, L. Effect of the damping function in dispersion corrected density functional theory. *J. Comput. Chem.* **32**, 1456-1465 (2011).

14 Grimme, S., Antony, J., Ehrlich, S. & Krieg, H. A consistent and accurate ab initio parametrization of density functional dispersion correction (DFT-D) for the 94 elements H-Pu. *The Journal of Chemical Physics* **132** (2010).

15 Barone, V. & Cossi, M. Quantum Calculation of Molecular Energies and Energy Gradients in Solution by a Conductor Solvent Model. *The Journal of Physical Chemistry A* **102**, 1995-2001 (1998).

16 Meng, E. C. *et al.* UCSF ChimeraX: Tools for structure building and analysis. *Protein Sci.* **32**, e4792 (2023).

17 Creutz, S. E. & Peters, J. C. Diiron Bridged-Thiolate Complexes That Bind N2 at the FeIIFeII, FeIIFeI, and FeIFeI Redox States. *J. Am. Chem. Soc.* **137**, 7310-7313 (2015).

18 Shen, Y. & Sullivan, B. P. A Versatile Preparative Route to 5-Substituted-1,10-Phenanthroline Ligands via 1,10-Phenanthroline 5,6-Epoxide. *Inorg. Chem.* **34**, 6235-6236 (1995).

19 Krishnan, S., Kuhn, D. G. & Hamilton, G. A. Direct oxidation in high yield of some polycyclic aromatic compounds to arene oxides using hypochlorite and phase transfer catalysts. *J. Am. Chem. Soc.* **99**, 8121-8123 (1977).
